# The Lipid Asset Is Unbalanced in Peripheral Nerve Sheath Tumors

**DOI:** 10.3390/ijms23010061

**Published:** 2021-12-22

**Authors:** Ignazio G. Vetrano, Michele Dei Cas, Vittoria Nazzi, Marica Eoli, Niccolò Innocenti, Veronica Saletti, Antonella Potenza, Tatiana Carrozzini, Giuliana Pollaci, Gemma Gorla, Rita Paroni, Riccardo Ghidoni, Laura Gatti

**Affiliations:** 1 Department of Neurosurgery, Fondazione IRCCS Istituto Neurologico Carlo Besta, 20133 Milan, Italy; vittoria.nazzi@istituto-besta.it (V.N.); niccolo.innocenti@unimi.it (N.I.); 2 Department of Health Sciences, Università degli Studi di Milano, 20142 Milan, Italy; michele.deicas@unimi.it (M.D.C.); rita.paroni@unimi.it (R.P.); 3 Molecular Neuro-Oncology Unit, Fondazione IRCCS Istituto Neurologico Carlo Besta, 20133 Milan, Italy; marica.eoli@istituto-besta.it; 4 Developmental Neurology Unit, Fondazione IRCCS Istituto Neurologico Carlo Besta, 20133 Milan, Italy; veronica.saletti@istituto-besta.it; 5 Neurobiology Laboratory, Fondazione IRCCS Istituto Neurologico Carlo Besta, 20133 Milan, Italy; antonella.potenza@istituto-besta.it (A.P.); tatiana.carrozzini@istituto-besta.it (T.C.); giuliana.pollaci@istituto-besta.it (G.P.); gemma.gorla@istituto-besta.it (G.G.); laura.gatti@istituto-besta.it (L.G.); 6 Neurorehabilitation Department, IRCCS Istituti Clinici Scientifici Maugeri, 20138 Milan, Italy; riccardo.ghidoni@unimi.it

**Keywords:** ceramide, phospholipids, lipidomic, neurofibroma, plexiform neurofibroma, PNST, schwannoma, sphingolipids

## Abstract

Peripheral nerve sheath tumors (PNSTs) include schwannomas, neurofibromas (NFs), and plexiform neurofibromas (PNFs), among others. While they are benign tumors, according to their biological behavior, some have the potential for malignant degeneration, mainly PNFs. The specific factors contributing to the more aggressive behavior of some PNSTs compared to others are not precisely known. Considering that lipid homeostasis plays a crucial role in fibrotic/inflammatory processes and in several cancers, we hypothesized that the lipid asset was also unbalanced in this group of nerve tumors. Through untargeted lipidomics, NFs presented a significant increase in ceramide, phosphatidylcholine, and Vitamin A ester. PNFs displayed a marked decrease in 34 out of 50 lipid class analyzed. An increased level of ether- and oxidized-triacylglycerols was observed; phosphatidylcholines were reduced. After sphingolipidomic analysis, we observed six sphingolipid classes. Ceramide and dihydroceramides were statistically increased in NFs. All the glycosylated species appeared reduced in NFs, but increased in PNFs. Our findings suggested that different subtypes of PNSTs presented a specific modulation in the lipidic profile. The untargeted and targeted lipidomic approaches, which were not applied until now, contribute to better clarifying bioactive lipid roles in PNS natural history to highlight disease molecular features and pathogenesis.

## 1. Introduction

Benign peripheral nerve sheath tumors (PNSTs) collectively, constitute 10–12% of benign soft-tissue neoplasms [1]; they include mainly schwannomas, the most common tumors arising from peripheral nerves, and neurofibromas [2,3]. Schwannomas are usually capsulated, well-circumscribed masses originating from a single fascicle. These tumors grow between the fascicles of peripheral nerves and, histologically, have variable compact spindled areas and hypocellular microcystic areas, rich in macrophages and collagen fibers [2,4]. Neurofibromas (NF) can show different growth patterns; either well-demarcated intraneural or diffuse infiltrative. NFs can present two or more entering and exiting fascicles, usually larger than those seen in schwannomas. Their capsule is more adherent to the central mass of the tumor than schwannomas. They are constituted by Schwann cells and a non-neoplastic fibrous component that includes fibroblasts. Plexiform neurofibromas (PNFs) often involve numerous adjacent nerve fascicles or multiple components of a plexus. PNFs present irregularly thickened, distorted, tortuous structures and are composed of neoplastic Schwann cells, fibroblasts, mast cells, macrophages, endothelial cells, nerve cells, and other cell types with abundant collagen deposition. They have the highest growth potential in childhood and adolescence, while the growth tends to slow down in adulthood [5,6,7]. These tumors can cause severe morbidity, including pain, neurological dysfunction, and disfigurement. PNSTs mainly occur sporadically, but they represent a distinct tract of neurocutaneous disorders such as schwannomatosis or neurofibromatosis type 1 (NF1). The latter is due to constitutional loss-of-function mutations of the *NF1* gene that encodes neurofibromin, which exerts a tumor suppressor activity by downregulating the Ras signaling pathway [8,9]. Even though NF1 is a rare disease, it represents one of the most frequent autosomal dominant syndromes predisposing to cancerogenesis [10]. PNFs, observed in approximately 50% of individuals with NF1, are one of the disease’s hallmarks, along with NF or cutaneous and ophthalmic alterations [7].

The mainstay of PNST treatment is surgical removal [1,3,11,12,13,14,15,16], although this approach is not always feasible, as in the case of huge schwannomas or PNFs. Moreover, although the different subtypes of PNST are considered benign tumors according to the biological behavior, PNFs differ from NFs and schwannomas due to their propensity for malignant degeneration. PNFs can transform into malignant PNSTs (MPNSTs), aggressive sarcomas associated with high mortality, despite surgery or adjuvant therapies [3,4,12,17,18]. The cumulative lifetime risk of developing MPNSTs is around 8–13% in all NF1 patients, but not all PNFs have the same potential to progress towards malignancy [7,19]. The specific factors contributing to the more aggressive behavior of some PNFs compared to others (or compared to schwannomas and neurofibromas) are not precisely known because of the extreme heterogeneity of PNFs and differences among patients [20,21]. In addition to the intrinsic tumorigenic drive, a heterogeneous and often unclear microenvironment modulates tumor progression. Globally, the coexistence of multiple immune cells suggests a complex inflammatory microenvironment in some PNSTs [22].

Lipid homeostasis plays a crucial role in fibrotic/inflammatory processes and in several tumor types [23]. Changes in lipid metabolism can affect cell growth, proliferation, and differentiation [24]. More recently, the role of lipid modulation in tumor aggressiveness has also been suggested for solid tumors like breast, colorectal, and prostate cancers through the interaction of tumor cells with stromal cells and the surrounding microenvironment [25,26,27]. Similarly, lipid metabolism and metabolic interactions within the tumor microenvironment contribute to pancreatic tumor progression [28]. Recently, lipid profiling has also been applied in mesothelioma and non-small cell lung carcinoma [29,30]. Moreover, in notochordal-derived tumors as chordomas, long- and very-long-chain ceramide (Cer) levels were associated with cancer cell survival and tumor aggressiveness [23]. Considering the histopathological characteristics of PNSTs and the different behaviors of some of them compared to others, we hypothesized that the lipid asset could be unbalanced in this set of specific tumors. To understand the cross-talk between the lipid profile and the tumor characteristics, we evaluated, for the first time, the lipidomic profile of different PNSTs obtained from neurosurgical procedures in patients with sporadic tumors or associated with neurocutaneous diseases. We performed an untargeted lipidomic approach to uncover the potential classes of lipids altered in nerve tumor samples versus control ones. We then investigated, by a targeted sphingolipidomic approach, the profile of this class of bioactive lipids, since sphingolipids are known to take part in immune response and can initiate or sustain inflammation and cellular apoptosis in inherited, fibrotic, and progressive diseases [31]. The role of deregulated sphingolipid profiles has not yet been explored in PNSTs, and such tumors lack studies addressing a pathogenic role for the lipid asset.

## 2. Results

### 2.1. Clinical Features of the PNST Patients’ Cohort

**Figure 1:**
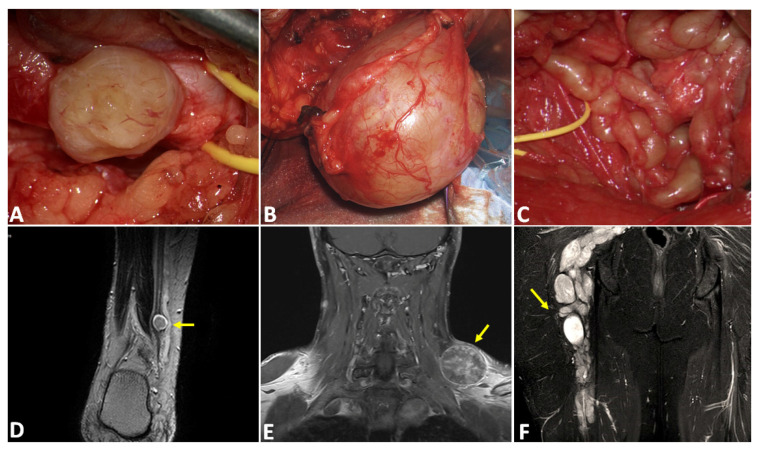
Differences among PNSTs at intraoperative visualization under microscopic view (**A**–**C**). Schwannomas (**A**) usually originate from a single fascicle and are easily distinguishable from the surrounding nerves. Neurofibromas (**B**) present two or more entering and exiting fascicles, and their capsules are more adherent to the tumor mass than schwannomas. Finally, plexiform neurofibromas (**C**) can greatly subvert the nerve architecture and involve more fascicles or an entire plexus. The lower panels (**D**–**F**) show the MRI of the tumors (yellow arrow) presented in the upper panels, respectively a schwannoma of the right sural nerve (**D**), a left brachial plexus neurofibroma (**E**), and a vast plexiform neurofibroma of the right femoral nerve (**F**).

The 44 total samples were obtained from a cohort of patients with a mean age of 49 years (SD ± 12.7, range 23–76 years); 53% were males and 47% females. Nine samples, in total, were represented by NF1 or schwannomatosis samples. All tumors depicted a clear contrast enhancement at preoperative MRI, even with different degrees of intensity and homogeneity (Figure 1).

### 2.2. Untargeted Lipidomics of PNSTs

**Figure 2:**
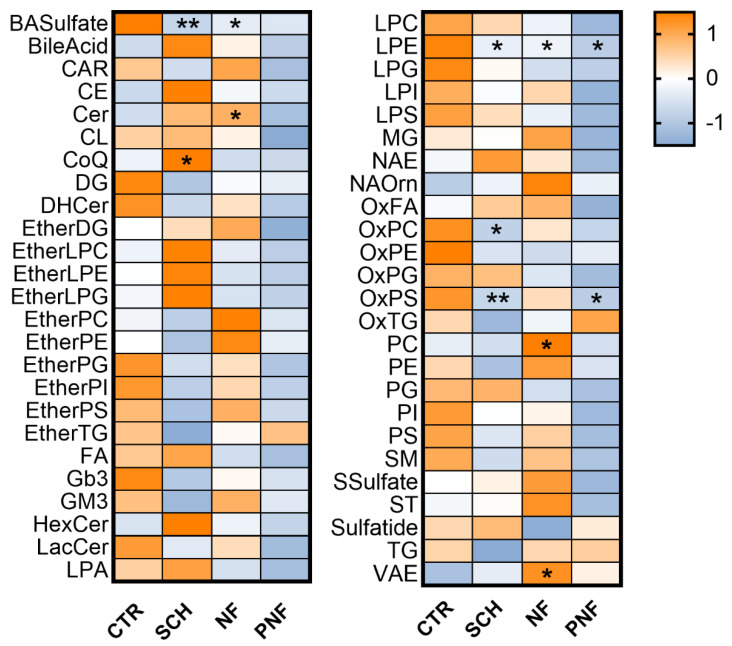
Heatmap of the principal lipid classes in schwannomas (SCH), neurofibromas (NF), and plexiform neurofibromas (PNFs) as compared to healthy nerves (CTR). The mass intensities are log-transformed and scaled to z-values for visualization. The color-scale differentiates values as high (orange), average (white), and low (blue). *p* values are schematized as follows: * < 0.05, ** < 0.01.

The untargeted lipidomic analysis was conducted on 26 samples derived from surgical treatment, comprising 12 schwannomas, 10 neurofibromas, and 4 PNFs. Statistical significance was evaluated by comparing the lipid profile between tumors and normal tissue specimens by one-way ANOVA with Bonferroni post hoc test. As compared to controls, schwannomas showed a marked increase of coenzyme Q10 (CoQ, *p* < 0.05), ether lysophospholipids, hexosylceramides (HexCer), whereas ether phospholipids, ether triglycerides, and sphingomyelins were reduced (Figure 2).

Neurofibromas presented a significant increase in Cer, phosphatidylcholine, and Vitamin A ester (VAE); on the other hand, oxidized phospholipids and triglycerides were decreased. For other lipids, the pattern was similar to that observed in schwannomas.

Noticeably, PNFs displayed a marked decrease in the 34/50 lipid class analyzed; on the contrary, an increased level of ether triacylglycerols (etherTG) and oxidized triacylglycerols (OxTG) was observed among triglycerides. Unlike neurofibromas, the VAE was not increased in PNFs compared to the control specimens. Phosphatidylcholines, which were significantly increased in NFs, were reduced in PNFs.

### 2.3. Targeted Sphingolipidomic Analysis of PNSTs

**Figure 3:**
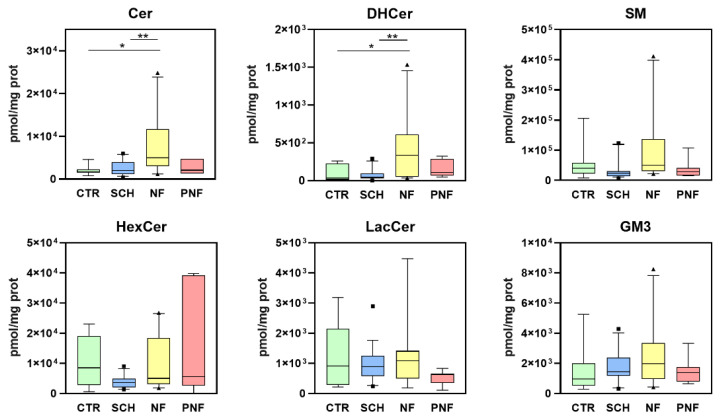
Targeted sphingolipidomic of PNST samples shows sphingolipid concentrations (pmol/mg prot) in schwannomas (SCH), neurofibromas (NF), plexiform neurofibromas (PNFs) as compared to healthy nerve tissues (CTR). The considered sphingolipids were ceramides (Cer), dihydroceramides (DHCer), sphingomyelins (SM), hexosylceramides (HexCer), lactosylceramides (LacCer), gangliosides (GM3). Boxes: 25th–75th percentiles; lines: 10th–90th percentiles; crossing lines: median values; separate points; outliers (square for SCH, triangle for SCH). *p* values: * < 0.05, ** < 0.01.

We evaluated the absolute sphingolipid concentrations among the different subtypes of PNST compared with healthy nerves by applying a targeted sphingolipidomic approach. The targeted analysis was conducted on 36 samples, comprising 19 schwannomas, 10 neurofibromas, and 7 PNFs. One-way ANOVA with Bonferroni post hoc test was applied to detect the statistical significance in sphingolipid concentrations among groups (* *p* < 0.05, ** *p* < 0.01). We observed six sphingolipid classes, namely Cer, dihydroceramide (DHCer), Sphingomyelin (SM), HexCer, lactosylceramide, and ganglioside (GM3). Among all these species, Cer and DHCer were statistically increased in NFs versus both control tissues and schwannomas (Figure 3). This result overlapped with what was observed by the lipidomic untargeted approach. The large individual variability and the relatively small number of analyzed samples did not show further potential alterations in the sphingolipid asset.

**Figure 4:**
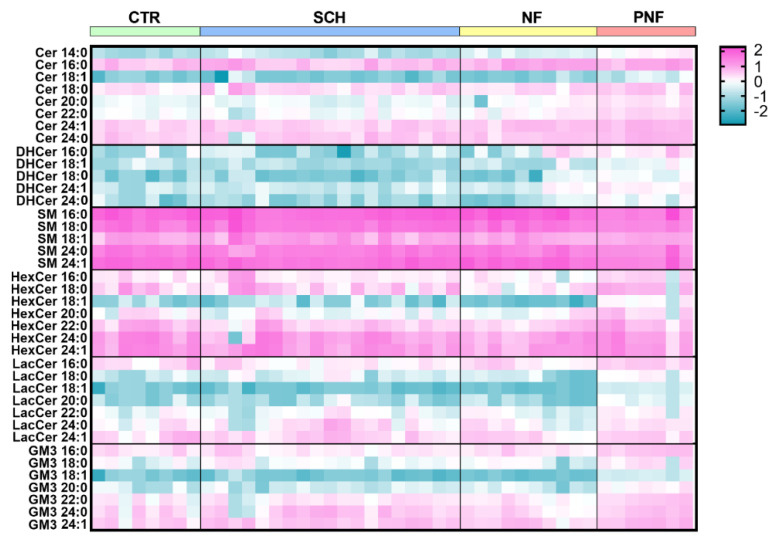
Heatmap of the main sphingolipid species in schwannomas (SCH), neurofibromas (NF), plexiform neurofibromas (PNFs) as compared to healthy nerves (CTR). The concentrations were log-transformed and scaled to z-values for visualization. The color-scale differentiates concentration values as high (purple), average (white), and low (blue).

Interesting evidence emerged when we explored the molecular species of sphingolipid classes by the heatmap shown in Figure 4. All the glycosylated species, namely HexCer, LacCer, and GM3, appeared reduced in NFs, but increased in PNFs versus controls, and this was more evident for those containing 18:1 fatty acid.

## 3. Discussion

This study was the first lipidomic analysis in human PNST surgical specimens. Using this approach, we obtained a lipid profile assessment among different subtypes of PNSTs (comprising schwannomas, NFs, and PNFs) derived from neurosurgical procedures in patients with sporadic or neurocutaneous-related tumors to investigate the impact of lipid alterations on tumors features. Deregulation of lipid metabolism occurred in several tumors, while the stimulation of lipid synthesis, affecting both cholesterol and fatty acid, may have resulted from the activation of oncogenic pathways [24]. Numerous cancer driver genes have been shown to control cellular metabolism, leading to the conception that a better knowledge of the mechanisms of lipid metabolic reprogramming in cancer is expected to unravel amenable therapeutic targets. Sphingolipids participate in immune response and can initiate and/or sustain inflammation and cellular apoptosis in inherited, fibrotic, and progressive diseases. Pharmacological inhibition of ceramide synthesis reduced chronic inflammation damage of bronchial epithelial cells in cystic fibrosis [32,33]. Previous studies have been aimed at determining lipid specific biological role in regulating apoptosis and proliferation pathways, demonstrating that a delicate equilibrium exists between long-chain (i.e., Cer C16:0, Cer C18:0, Cer C20:0) and very-long-chain (Cer C24:1, Cer C24:0) ceramides [23,34].

Despite the limited sample size and large individual variability, untargeted lipidomic analysis shows different lipid profiles in the three investigated histotypes. Our findings suggested that a peculiar lipid profile (Supplementary Figure S1) characterized different benign tumors, although they share a common biological origin. This evidence led us to speculate the existence of specific alterations concerning the underpinning cellular pathological mechanisms. Despite the shared features of benign tumors, their growth mechanisms are different.

In schwannomas, the increased CoQ—a mitochondrial factor with potent antioxidant properties [35]—may be interpreted as a defense mechanism to counteract possible tumor cell damage due to oxidative events. In some familial schwannomatosis, alteration of CoQ and impaired production of reactive oxygen in Schwann cells (the main component of schwannomas) have been noted [36]. Among ether lysophospholipids, the primary member is the Platelet Activating Factor (PAF) [37], a potent effector of inflammation produced by basophils, which could be recruited at the tumor site. The enhanced level of ether lysophospholipids in schwannomas agreed with an inflammatory hallmark present in these tumors, as previously reported for vestibular schwannomas [38,39]. Pro-inflammatory cytokines, including TGF-β1, TNF-α, IL-1β, and IL-6, may be secreted by activated leukocytes, fibroblasts, and Schwann cells [39]. Injuries of peripheral nerves determine the inflammatory response of Schwann cells, exhibiting cytokine upregulation [40]. Moreover, Schwann cells present striking plasticity, which leads them to play a crucial repair function in peripheral nerve alterations [41].

Furthermore, when analyzed by untargeted lipidomics, HexCer appeared elevated in these tumors, but its level did not differ significantly from healthy control nerves when a more specific targeted sphingolipidomic approach was applied. Other lipid species were reduced in schwannomas as compared to control nerve tissues. These included ether phospholipids, a particular class of phospholipids called plasmalogens, in which the glycerol backbone has an ether or vinyl-ether bond at the sn-1 position sphingomyelin [42]. Both plasmalogens and sphingomyelin are present in the brain, and myelin is particularly enriched [43,44]. Thus, their reduction can be associated with a local demyelination process that creates a possible disturbance in physiological nerve development, considering the schwannoma cytoarchitecture [45]. Regarding the low levels of ether triglycerides, this class is present in lipid droplets, but its precise function has not been seriously addressed. A decrease of ether lipid in neurodegenerative diseases and metabolic disorders, as well as schwannomas, strengthens the interest in this relatively unexplored class of lipids [46].

In neurofibromas, the lipid asset is characterized by a significant increase of Cer, compared to both controls and the other classes of PNST, as clearly evidenced by untargeted and targeted analyses. Cer, a key sphingolipid mediator, is associated with an inflammatory trait present in neurofibromas. Moreover, it is generally assumed to exert antiproliferative responses, such as cell growth inhibition, apoptosis induction, senescence, endoplasmic reticulum stress responses and/or autophagy [47,48].

Interestingly, even if most solid cancers express low Cer levels—supporting the high proliferation rate and the lack of the apoptotic trait—other studies have shown that Cer levels are increased in some tumors and that de novo-generated Cer may have distinct and opposing roles in the promotion/suppression of tumors [49]. The high Cer level observed in neurofibroma may reflect a less pronounced attitude towards tumor progression. The untargeted lipidomic of NFs showed an increase of VAE, which is the form of intracellular storage of retinol. Retinyl esters are hydrolyzed to release retinol, which is oxidized to retinaldehyde and retinoic acid. The latter promotes differentiation and is known to increase the levels of Cer [50]. Such a mechanism could therefore affect the Cer increase in neurofibromas. Moreover, Vitamin A is a strong antioxidant agent, which can justify the decrease of oxidized- phospholipids and triglycerides found in these tumors.

PNFs diverged dramatically from other included PNSTs due to their lower lipid content as compared to controls and other tumor subtypes (see Figure 2). This general feature did not appear to be restricted to specific classes. We hypothesized a general decrease of cellular components concurrently to increased extracellular components and collagen deposition. There was no evidence for antioxidant agents in PNFs, as opposed to other PNSTs, as confirmed by the observed increase of oxidized triglycerides. The oxidative trait of PNFs may therefore favor the more aggressive behavior of PNFs in comparison with other PNSTs. To better clarify this aspect, an ongoing study aims to collect MPNST samples arising from PNFs, to increase knowledge of the role of the lipid asset and correlate it with the tumor progression.

The disbalance in the lipid asset found in these classes of tumors could be partially explained by changes in the activity of related enzymes. In particular, when referring to the sphingolipid profile, the possible enzymes involved were those regulating Cer content, either in its de novo biosynthesis (serine palmitoylCoa transferase, Cer desaturase, Cer synthase) or in its pivotal role in the complex network of the sphingolipid turnover (glucosyceramidase, Cer glucosyltransferase, ceramidase, sphingomyelin synthase, sphingomyelinase, sphingosine kinases). All these enzymes were present in different forms, a further difficulty to consider. In collecting new PNST samples, future efforts will also focus on the activity assessment of some enzymes, or at least on determining their gene expression or protein level. Clearly, we cannot expect to generate, in the near future, a complete picture of the enzyme-related changes in the lipid asset of these tumors or their limited occurrence, but we are well aware that this is the correct direction to follow.

**Figure 5:**
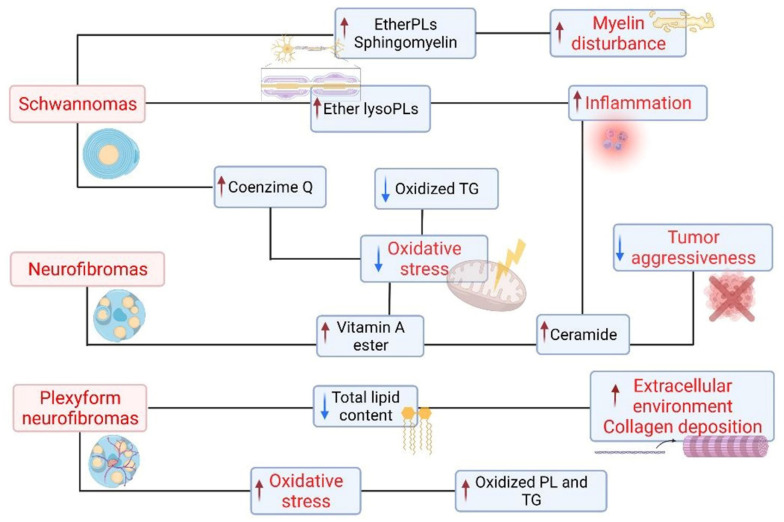
Schematic map representing the main lipid factors altered among the subset of PNST analyzed and the putative associated biological effects.

A scheme summarizing the lipid asset and cellular biological responses in PNSTs is reported in Figure 5.

Our study had some limitations. First, the sample size of PNST patients could suffer from selection bias since strict control matching among sporadic and neurocutaneous disease-related tumors was not fully respected. Thus, our findings should be verified in a larger cohort and other biological samples.

## 4. Materials and Methods

### 4.1. Patient and Surgical Protocol

The tumor samples were obtained, after informed consent, from consecutive patients admitted at the Peripheral Nerve Service of our Institution for suspected PNST from January 2020 to June 2021. Privacy procedures were applied to protect patients’ and healthy controls’ identities. Indications to tumor removal were: increasing volume, the onset of pain or new neurological signs, or radiological and clinical characteristics suspicious for atypical transformation. The unrelated control tissues were represented by nerves samples obtained from sural nerve biopsies (performed for different suspected diagnoses) or from the institutional biobank. Histopathological analysis was performed in each case by the neuropathology group of our Institute, based on the 2016 WHO Classification [51]. All patients were submitted to preoperative contrast MRI of the nerves interested. Neurosurgical procedures were carried out under general anesthesia and microscopic view. According to our institutional protocol for peripheral nerve surgery and the literature recommendations [9,10,38], intraoperative neurophysiological monitoring was performed when indicated to identify functioning fascicles and localize the safest entry point inside the tumor capsule. Immediately after excision, all tumor samples for lipidomic were cryopreserved and stored in liquid nitrogen at the Neurobiology laboratory according to standard operating procedures [52].

### 4.2. Lipidomic Analysis on Tumors and Control Tissues

The schwannomas, NFs, PNFs, and control tissues were subjected to both an untargeted lipidomic profile and a targeted analysis to determine the absolute concentrations of sphingolipids. Targeted analysis was conducted on the first set of samples; the targeted one comprised some of the specimens’ residual from the targeted study, with the addition of newly collected samples Targeted and untargeted analyses were normalized for the samples’ protein content, evaluated by Bradford assay.

The chemicals acetonitrile, 2-propanol, methanol, chloroform, formic acid, ammo-nium acetate, ammonium formate and dibutylhydroxytoluene (BHT) were purchased from Sigma-Aldrich (St. Louis, MO, USA). All aqueous solutions were prepared using purified water at a Milli-Q grade (Burlington, MA, USA).

#### 4.2.1. Untargeted Lipidomics

Total lipid extraction was completed on schwannomas, neurofibromas, PNF samples and control tissues by a modified Bligh & Dyer method [33]. The extracts were analyzed, in both positive and negative electrospray ionization, by LC-MS/MS consisting of a Shimadzu UPLC coupled with a Triple TOF 6600 Sciex (Concord, ON, CA) equipped with Turbo Spray IonDrive. Spectra were contemporarily acquired by both full-mass scans from *m*/*z* 200–1500 and top-20 data-dependent acquisition from *m*/*z* 50–1500. The separation was achieved by an Acquity CSH C18 column 1.7 μm 2.1 × 100 mm (Waters, MA, USA) using mobile phase (A) water/acetonitrile (60:40) and a mobile phase (B) 2-propanol/acetonitrile (90:10) both containing 10 mM ammonium acetate and 0.1% formic acid. The flow rate was 0.4 mL/min, and the column temperature was 55 °C. Raw data were processed using MS-DIAL (ver. 4.70, RIKEN Center for Sustainable Resource Science, Yokohama, Japan, http://prime.psc.riken.jp/compms/msdial/main.html, accessed on 1 December 2020), peak-identified by comparison with LipidBlast library, and then normalized [33]. The lipidomic analysis included the following lipid classes, each comprising different lipid species: sterols (cholesterol and cholesterol esters), acylglycerols (mono-, di-, tri-acylglycerols), phospholipids (PC, PE, PI, lyso-PC, lyso-PE, SM), sphingolipids (Cer, DhCer, HexCer, LacCer, Gb3, and GM3), free fatty acids (long- and very long-chain, saturated, monounsaturated, polyunsaturated), lipid ethers (cardiolipin and plasmalogens). The complete list of lipids identified can be found in the Supplementary Tables S1 and S2.

#### 4.2.2. Targeted Sphingolipidomics

Sphingolipids were extracted from schwannomas, neurofibromas, PNF samples and control tissues by using a monophasic extraction coupled with alkaline methanolysis [36]. The extract was analyzed by LC-MS/MS consisting of a UPLC (Dionex 3000 UltiMate, Thermo Fisher Scientific, Waltham, MA, USA) connected to a 3200 QTRAP Sciex (Concord, Toronto, ON, Canada). Separation was attained on an Acquity BEH C8 1.7 μm, 2.1 × 100 mm (Waters, Franklin, MA, USA), equipped with a precolumn by mixing eluent A (water + 2 mM ammonium formate + 0.2% formic acid) and eluent B (methanol + 1 mM ammonium formate + 0.2% formic acid). The flow rate was 0.3 mL/min, and the column temperature was 30 °C. Multiple reaction monitoring (MRM) was used to quantify the concentrations of each sphingolipid (DHCer, Cer, HexCer, LacCer, GM3, SM with fatty acids from C16 to C24) considered in this study [53]. The complete list of sphingolipids monitored can be found in the Supplementary Table S3.

### 4.3. Statistical Analysis

Graphs and statistical analyses were prepared with GraphPad Prism 7.0 (GraphPad Software, Inc., La Jolla, CA, USA). Univariate statistical analysis was performed using one-way ANOVA with Bonferroni post hoc test to compare lipid concentrations across different groups. A *p* < 0.05 was considered statistically significant. Lipid concentrations were log-transformed, without zero correction, and scaled to z-values for visualization to make features more comparable.

## 5. Conclusions

Our findings suggested that different subtypes of PNSTs present peculiar lipid profiles. The untargeted and targeted lipidomic approaches, which were not applied until now, contributed to better clarifying bioactive lipids’ role in PNS natural history to highlight diseases’ molecular features and pathogenesis. Further studies will aim to identify specific lipid signatures among different PNSTs, with the final goal of identifying possible targets for precision medicine.

## Data Availability

Data supporting reported results can be found in publicly archived datasets generated during the study at the Fondazione IRCCS Istituto Neurologico Carlo. Besta (https://zenodo.org/communities/besta/?page=1&size=20; accessed on 15 November 2021).

## Supplementary Materials

The following supporting information can be downloaded at: https://www.mdpi.com/article/10.3390/ijms23010061/s1.

## Abbreviations

BASulfate: colic acids sulfate; BileAcid, colic acid; Car, Acylcarnitine; CE, cholesterol ester; Cer, ceramide; CL, cardiolipines, chol, cholesterol; CoQ, coenzyme Q10; DHCer, dihydroceramide; FA, free fatty acid; Gb3, globotriaosylceramide; GM3, ganglioside; HexCer, hexosylceramides; LacCer, lactosylceramide; LPA, lysophosphatidylacid; LPC, lysophosphatidylcholine; LPE, lysophosphatidylethanolamine; LPG, lysophosphatidylglicerol; LPG, lysophosphatidylinositole; LPS, lysophosphatidylserine; MG, monoacylglicerol; MPNST, malignant peripheral nerve sheath tumors; NAE, n-acylethanolamine; NAOrn, n-acylornithine; NF1, neurofibromatosis type 1; NF, neurofibromas; Ox, oxidized; PC, phosphatidylcholine; PE, phosphatidylethanolamine; PG, phosphatidylglicerol; PI, phosphatidylinositole; PNF, plexiform neurofibromas; PNST, peripheral nerve sheath tumors; PS, phosphatidylserine; Sch, schwannomas; SM, sphingomyelin; SSulfate, sterol sulfate; ST, sterol; TG, triacylglicerol; VAE, vitamin A fatty acid ester.
