# Supplementary material for: The Lipid Asset Is Unbalanced in Peripheral Nerve Sheath Tumors
Source: Int J Mol Sci. 2021 Dec 22;23(1):61. doi: 10.3390/ijms23010061 (PMC8744637; doi:10.3390/ijms23010061)
Supplement: Supplementary file 1 [file ijms-23-00061-s001.zip › ijms-1512004-supplementary.pdf]

International Journal of *Molecular Sciences*

MDPI

Supplementary materials

The Lipid Asset is Unbalanced in Peripheral Nerve Sheath Tumors

Ignazio G. Vetrano[1,†,*], Michele Dei Cas[2,†], Vittoria Nazzi[1], Marica Eoli[3], Niccolò Innocenti[1], Veronica Saletti[4], Antonella Potenza[5], Tatiana Carrozzini[5], Giuliana Pollaci[5], Gemma Gorla[5], Rita Paroni[2], Riccardo Ghidoni[6], and Laura Gatti[5]

[1] Department of Neurosurgery, Fondazione IRCCS Istituto Neurologico Carlo Besta, 20133 Milan, Italy; ignazio.vetrano@istituto-besta.it; vittoria.nazzi@istituto-besta.it; niccolo.innocenti@unimi.it
[2] Department of Health Sciences, Università degli Studi di Milano, 20142, Milan, Italy; michele.deicas@unimi.it; rita.paroni@unimi.it
[3] Molecular Neuro-Oncology Unit, Fondazione IRCCS Istituto Neurologico Carlo Besta, 20133 Milan, Italy; marica.eoli@istituto-besta.it
[4] Developmental Neurology Unit, Fondazione IRCCS Istituto Neurologico Carlo Besta, 20133 Milan, Italy; veronica.saletti@istituto-besta.it
[5] Neurobiology Laboratory, Fondazione IRCCS Istituto Neurologico Carlo Besta, 20133 Milan, Italy; laura.gatti@istituto-besta.it; antonella.potenza@istituto-besta.it; tatiana.carrozzini@istituto-besta.it; giuliana.pollaci@istituto-besta.it; g.gorla10@campus.unimb.it
[6] Neurorehabilitation Department, IRCCS Istituti Clinici Scientifici Maugeri, 20138 Milan, Italy; riccardo.ghidoni@unimi.it

* Correspondence: ignazio.vetrano@istituto-besta.it
† Contributed equally

Index of contents.

Figure S1. Principal component analysis for the **(A)** untargeted lipidomics and **(B)** targeted sphingolipidomics. Multivariate analysis was performed by the free available software MetaboAnalyst 5.0. Data were checked for integrity, filtered by interquartile range, generalized log-transformed, and auto-scaled prior to the multivariate analysis.
Table S1. List of all the single lipid species identified using an untargeted lipidomics approach across each histotype. Each lipid was identified with monoisotopic molecular adducts in positive electrospray ionization (m/z), name, and mass intensities scaled for protein content in each group.
Table S2. List of all the single lipid species identified using an untargeted lipidomics approach across each histotype. Each lipid was identified with monoisotopic molecular adducts in negative electrospray ionization (m/z), name, and mass intensities scaled for protein content in each group.
Table S3. List of all the sphingolipids species identified using a targeted approach across each histotype. Each sphingolipid was identified with multiple reaction monitoring mass transition (MRM) in positive electrospray ionization, name, and concentration (pmol/mg protein) in each group.

Figure S1. Principal component analysis for the **(A)** untargeted lipidomics and **(B)** targeted sphingolipidomics. Multivariate analysis was performed by the free available software MetaboAnalyst 5.0 (ver. 5.0, McGill Data Center and Compute Canada, Montréal, Canada, https://www.metaboanalyst.ca, accessed on 18 December 2021). Data were checked for integrity, filtered by interquartile range, generalized log-transformed, and auto-scaled prior to the multivariate analysis.

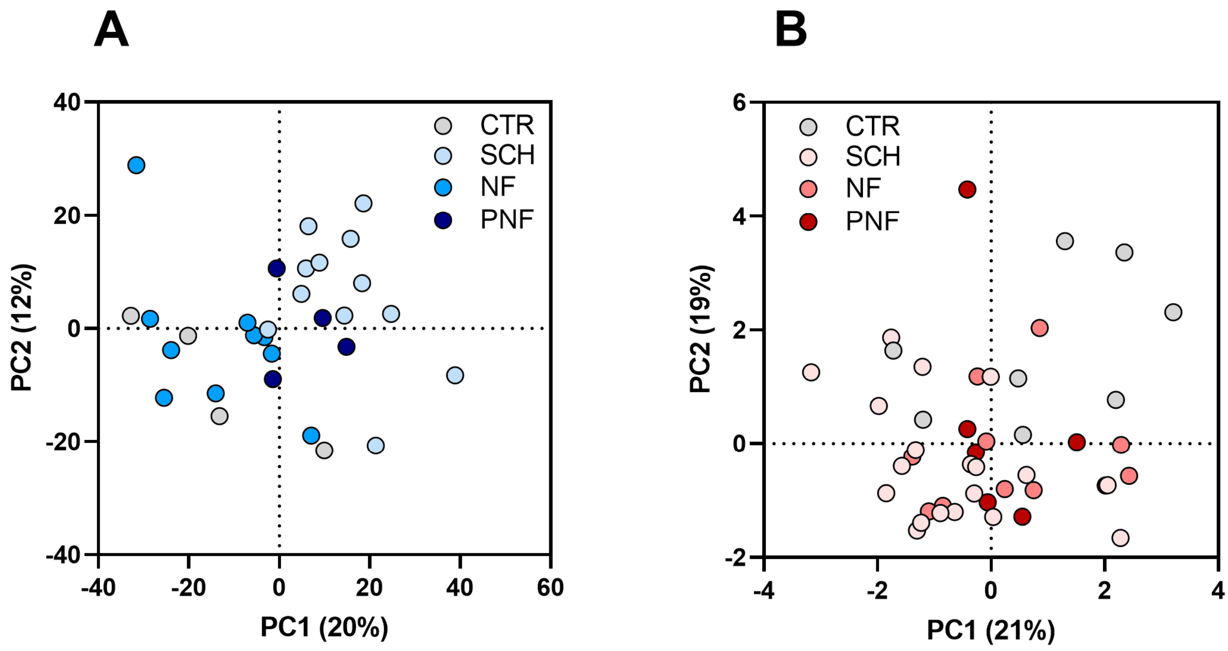

Table S1. List of all the single lipid species identified using an untargeted lipidomics approach across each histotype. Each lipid was identified with monoisotopic molecular adducts in positive electrospray ionization (*m*/*z*), name, and mass intensities scaled for protein content in each group.

**Mass**	**Metabolite name**	**CTR**		**SCH**		**NF**		**PNF**	
m/z		*mean*	*dev*	*mean*	*dev*	*mean*	*dev*	*mean*	*dev*
372.31039	CAR 14:0	3580.64	2116.52	2330.05	1275.36	4827.49	4045.71	1453.51	643.71
396.31424	CAR 16:2	50.90	40.83	80.76	105.40	138.28	108.77	75.86	81.21
394.29416	CAR 16:3	211.08	222.76	131.09	163.10	314.89	285.73	39.50	18.27
414.35721	CAR 17:0	1497.17	650.51	694.22	357.49	1028.75	1302.43	1135.00	356.97
426.35919	CAR 18:1	94.25	101.06	55.76	44.38	180.46	201.85	28.35	15.54
442.39233	CAR 19:0	139.82	55.89	93.18	53.94	171.70	251.86	98.19	37.86
432.31818	CAR 19:5	193.62	245.18	59.31	33.25	73.68	67.73	61.57	11.75
456.40451	CAR 20:0	612.80	936.78	168.66	122.21	979.63	1764.55	122.48	104.02
446.32483	CAR 20:5	52.07	28.16	34.31	33.92	63.39	40.16	14.01	3.47
484.43344	CAR 22:0	80.63	101.25	33.17	22.83	166.24	262.50	25.70	18.47
482.42075	CAR 22:1	482.70	811.88	139.18	96.89	338.44	206.84	83.28	67.05
478.38885	CAR 22:3	66.79	80.65	41.75	42.80	86.71	51.92	15.27	7.73
512.46558	CAR 24:0	165.65	161.24	90.42	89.63	206.84	184.60	48.38	26.55
510.45575	CAR 24:1	178.35	199.72	107.29	95.69	218.12	190.15	58.59	49.49
508.44031	CAR 24:2	48.60	62.21	27.18	18.51	57.63	38.16	11.94	7.61
504.40573	CAR 24:4	88.89	108.95	70.32	40.26	168.88	137.57	32.56	16.46
540.5014	CAR 26:0	121.75	74.46	85.20	64.27	261.96	228.90	49.27	15.65
538.48688	CAR 26:1	112.75	89.70	69.96	58.32	105.09	90.72	32.34	18.95
668.63452	CE 18:1	16.22	24.56	41.24	48.63	46.15	94.65	23.24	16.93
666.61658	CE 18:2	461.94	782.29	531.17	286.64	445.01	691.45	523.63	498.65
664.60181	CE 18:3	20.74	36.86	48.37	38.68	23.87	48.66	22.58	20.86
694.65649	CE 20:2	2.87	5.27	22.87	44.23	15.96	46.17	1.44	0.88
692.63562	CE 20:3	40.59	79.46	227.74	451.47	126.32	367.62	26.07	24.29
688.6059	CE 20:5	64.45	124.49	218.02	317.54	70.35	187.64	130.37	201.66

**Mass** *m/z*	**Metabolite name**	**CTR**		**SCH**		**NF**		**PNF**	
		mean	*dev*	*mean*	*dev*	*mean*	*dev*	*mean*	*dev*
722.68573	CE 22:2	6.17	10.33	17.19	45.10	6.51	19.48	0.57	0.31
718.65479	CE 22:4	37.05	72.62	325.82	703.74	126.45	389.09	5.31	6.00
714.60785	CE 22:6	3.46	3.52	30.20	62.54	4.20	8.07	14.86	23.09
748.70032	CE 24:3	2.68	4.87	5.38	7.42	4.43	13.03	0.49	0.46
744.66724	CE 24:5	34.77	68.56	29.15	46.41	13.66	39.36	1.47	1.42
772.69507	CE 26:5	54.72	107.31	9.63	10.53	4.35	11.53	1.03	1.08
388.30649	Cer 21:1;4O	122.76	99.11	111.18	82.92	254.75	205.34	50.86	27.94
368.31384	Cer 22:1;3O|Cer 14:1;2O/8:0;O	432.98	432.37	259.18	134.80	683.16	653.73	185.94	154.20
408.30661	Cer 24:3;4O	12982.24	13767.16	6333.14	3800.74	22649.61	23190.90	4747.00	3092.82
440.37344	Cer 26:1;4O	432.01	451.08	442.73	909.14	146.37	155.79	160.64	43.86
436.33759	Cer 26:3;4O	359.87	150.10	277.48	214.93	272.98	207.35	328.78	182.10
464.37399	Cer 28:3;4O	204.30	176.65	182.93	166.47	280.72	202.01	116.93	29.60
476.37625	Cer 29:4;4O	25.14	19.64	101.53	241.96	57.48	118.36	9.22	1.20
464.44751	Cer 30:1;2O|Cer 18:1;2O/12:0	30.76	21.22	3921.16	11588.35	117.52	113.33	25.02	8.06
478.46606	Cer 31:1;2O|Cer 17:1;2O/14:0	23.51	10.87	74.65	101.33	239.78	227.74	47.81	25.32
498.35022	Cer 31:7;4O	219.46	334.76	8.76	6.42	89.54	181.11	8.97	9.58
528.50061	Cer 32:0;3O|Cer 16:0;2O/16:0;O	5.08	3.83	6.66	12.27	153.57	440.67	2.97	1.23
510.49121	Cer 32:1;2O|Cer 16:1;2O/16:0	102.79	66.84	110.87	88.96	900.33	1259.02	86.89	32.94
492.47229	Cer 32:1;2O|Cer 18:1;2O/14:0	200.26	150.13	219.44	172.10	1817.55	2602.98	153.81	62.31
508.47073	Cer 32:1;3O|Cer 18:1;2O/14:0;O	8.13	5.39	8.65	11.47	174.79	455.32	7.98	2.32
508.47504	Cer 32:1;3O|Cer 19:0;2O/13:1;O	3.64	2.77	5.09	10.67	87.98	246.82	2.03	0.52
490.46143	Cer 32:2;2O|Cer 18:2;2O/14:0	38.36	26.40	120.08	156.74	143.82	85.40	48.14	19.25
542.51837	Cer 33:0;3O|Cer 16:0;2O/17:0;O	0.67	0.89	0.19	0.28	86.23	241.10	0.30	0.21
558.51184	Cer 33:0;4O|Cer 17:0;3O/16:0;(2OH)	2.13	1.02	3.14	6.18	91.27	260.29	1.56	0.95
522.49011	Cer 33:1;3O|Cer 18:0;2O/15:1;O	2.76	1.55	3.22	4.93	251.30	763.92	1.72	0.47

Mass *m/z*	Metabolite name	CTR		SCH		NF		PNF	
		mean	*dev*	*mean*	*dev*	*mean*	*dev*	*mean*	*dev*
536.47113	Cer 33:2;4O	236.25	275.49	7.60	5.51	94.86	103.45	17.96	26.11
518.45166	Cer 33:3;3O | Cer 18:2;2O/15:1;O	27.00	10.89	41.92	58.20	92.46	64.99	33.18	11.32
540.53217	Cer 34:0;2O | Cer 18:0;2O/16:0	2.78	1.46	77.35	177.10	10.19	13.15	2.50	2.81
520.50708	Cer 34:1;2O | Cer 18:1;2O/16:0	290.27	306.19	46.30	39.67	198.13	178.56	65.39	17.95
554.51318	Cer 34:1;3O | Cer 18:1;2O/16:0;O	14.35	10.59	22.45	36.83	133.44	228.30	28.15	22.19
536.50372	Cer 34:1;3O | Cer 19:0;2O/15:1;O	30.43	28.42	31.40	39.50	1892.13	5680.23	25.36	12.94
552.49774	Cer 34:1;4O | Cer 18:1;3O/16:0;(2OH)	4.63	3.05	7.19	13.01	207.96	617.69	2.39	0.71
518.49335	Cer 34:2;2O | Cer 18:2;2O/16:0	31.88	26.14	238.35	406.52	48.99	41.53	15.68	12.88
534.48529	Cer 34:2;3O | Cer 18:2;2O/16:0;O	2.05	1.42	1.89	2.97	55.03	155.31	1.25	0.33
568.49231	Cer 34:2;4O | Cer 19:1;3O/15:1;(2OH)	1.94	1.21	1.23	1.37	55.89	161.50	1.50	0.94
560.42419	Cer 34:6;4O	156.93	136.40	163.07	273.59	81.44	73.89	78.58	16.29
566.51501	Cer 35:2;3O | Cer 19:0;2O/16:2;O	4.58	2.63	5.62	9.85	130.06	382.26	3.42	1.89
552.40881	Cer 35:8;4O	877.82	897.77	229.56	151.88	261.41	287.54	325.50	94.08
566.55939	Cer 36:0;3O | Cer 18:0;2O/18:0;O	17.51	5.92	37.59	68.10	38.91	37.53	5.32	2.75
548.53607	Cer 36:1;2O | Cer 18:1;2O/18:0	36.00	53.97	72.81	93.86	58.93	65.83	31.24	21.36
546.52325	Cer 36:2;2O | Cer 18:2;2O/18:0	22.49	28.57	55.63	95.42	18.35	12.63	13.76	9.25
562.53174	Cer 36:2;3O | Cer 16:1;2O/20:1;O	0.90	1.27	4.61	14.21	1.49	1.63	58.56	116.15
562.51776	Cer 36:2;3O | Cer 18:2;2O/18:0;O	1.95	0.49	18.14	37.80	12.21	12.83	2.35	1.04
576.49585	Cer 36:3;4O	71.90	34.28	82.71	56.42	601.38	975.60	202.88	144.31
574.48407	Cer 36:4;4O	5.50	3.98	4.36	3.34	46.55	117.31	4.30	1.61
570.45508	Cer 36:6;4O	174.91	126.53	168.73	302.37	1877.63	3105.12	71.31	28.86
568.44653	Cer 36:7;4O	164.18	124.38	51.20	25.45	73.50	72.94	65.29	7.23
568.42792	Cer 36:8;3O | Cer 12:2;2O/24:6;O	4.93	2.84	93.95	122.43	144.90	191.36	58.79	74.46
562.55872	Cer 37:1;2O | Cer 16:1;2O/21:0	8.73	1.52	36.30	36.46	91.53	80.82	13.11	6.61
600.46442	Cer 37:7;4O	68.15	54.59	51.24	64.44	404.45	482.98	27.13	7.85

Mass m/z	Metabolite name	CTR		SCH		NF		PNF	
		mean	dev	mean	dev	mean	dev	mean	dev
578.58679	Cer 38:0;2O I Cer 18:0;2O/20:0	894.90	731.01	144.83	208.57	343.42	272.09	185.76	312.85
576.57239	Cer 38:1;2O I Cer 16:1;2O/22:0	502.97	466.49	493.97	342.73	2395.58	2356.23	266.27	247.01
572.53265	Cer 38:3;2O I Cer 19:0;2O/19:3	1.68	1.41	1.62	1.96	110.62	329.02	0.89	0.33
598.49377	Cer 38:6;4O	137.45	227.22	48.35	107.72	85.55	104.01	23.01	31.41
614.47083	Cer 38:7;4O	216.07	245.79	536.44	1344.05	118.75	97.12	110.26	22.96
640.59265	Cer 39:1;4O I Cer 18:0;3O/21:1;(2OH)	395.49	267.03	78.05	48.38	536.07	967.80	180.60	202.09
622.59058	Cer 39:2;3O I Cer 18:1;2O/21:1;O	112.79	52.32	85.65	70.54	154.20	167.86	43.55	19.13
616.53387	Cer 39:4;4O	45.16	26.77	35.95	24.79	44.09	51.94	33.76	15.95
636.59137	Cer 40:2;3O I Cer 18:1;2O/22:1;O	262.25	129.07	52.49	24.73	387.62	525.53	105.33	65.52
616.56598	Cer 40:3;3O I Cer 18:3;2O/22:0;O	28.85	14.75	23.61	15.73	112.76	57.15	26.13	30.60
634.6134	Cer 41:1;3O I Cer 18:1;2O/23:0;O	747.41	1025.90	134.55	156.21	336.05	527.71	191.58	200.35
650.60797	Cer 41:2;3O I Cer 18:2;2O/23:0;O	11.62	8.94	12.89	16.85	121.39	338.34	11.22	7.43
630.58789	Cer 41:3;3O I Cer 17:0;2O/24:3;O	104.84	58.93	89.80	42.67	113.23	99.29	52.05	27.46
684.65295	Cer 42:0;4O I Cer 18:0;3O/24:0;(2OH)	6.58	2.46	5.82	2.16	553.88	1724.02	11.10	9.41
648.62854	Cer 42:1;3O I Cer 18:1;2O/24:0;O	23.76	34.01	6.27	7.71	5.37	6.75	17.80	23.35
630.61896	Cer 42:2;2O I Cer 18:1;2O/24:1	3.90	1.07	33.09	51.36	2.99	2.31	10.17	11.60
626.58594	Cer 42:4;2O I Cer 18:2;2O/24:2	104.75	77.31	160.64	154.02	227.91	144.85	52.00	29.63
622.55249	Cer 42:6;2O I Cer 18:1;2O/24:5	94.49	60.11	22.06	15.05	153.74	292.43	54.93	63.40
698.66895	Cer 43:0;4O	10.42	3.93	7.46	6.79	497.02	1556.26	4.16	2.19
646.64911	Cer 43:1;2O I Cer 18:1;2O/25:0	34.18	18.90	187.43	156.83	142.63	283.70	35.49	22.08
662.64801	Cer 43:1;3O I Cer 20:0;2O/23:1;O	8.79	10.73	5.44	2.45	454.77	1402.38	10.46	8.15
696.68829	Cer 44:0;3O I Cer 19:0;2O/25:0;O	9.34	3.75	9.61	4.34	1402.88	4401.29	6.80	2.16
676.66168	Cer 44:1;3O I Cer 18:1;2O/26:0;O	17.55	16.24	18.30	16.08	172.24	503.43	17.39	15.62
658.64709	Cer 44:2;2O I Cer 18:1;2O/26:1	19.92	16.62	89.42	119.67	30.14	33.28	10.39	7.66
688.6377	Cer 44:3;4O	11.46	11.10	33.46	49.38	16.09	12.30	10.13	5.67
654.61719	Cer 44:4;2O I Cer 18:2;2O/26:2	52.54	25.56	82.87	124.37	35.83	23.04	54.47	88.62

**Mass** *m/z*	**Metabolite name**	**CTR**		**SCH**		**NF**		**PNF**	
		mean	*dev*	*mean*	*dev*	*mean*	*dev*	*mean*	*dev*
684.57697	Cer 44:5;4O|Cer 18:0;3O/26:5;(2OH)	69.91	79.68	8.77	8.40	26.30	26.93	44.42	62.17
666.58105	Cer 44:6;3O|Cer 18:1;2O/26:5;O	465.75	519.47	47.40	48.87	80.07	55.14	49.12	29.32
680.57953	Cer 45:6;3O|Cer 19:0;2O/26:6;O	47.98	43.41	148.52	257.13	39.70	34.14	15.04	13.94
710.60785	Cer 46:6;4O|Cer 18:1;2O/28:5;2O	284.31	385.86	562.12	810.06	392.64	419.01	217.21	149.09
778.63806	Cer 50:9;4O|Cer 18:2;2O/32:7;2O	27.05	38.44	19.71	35.84	25.64	27.97	9.57	8.99
814.74841	Cer 53:3;4O	81.48	99.59	45.53	96.44	120.03	222.93	64.49	86.17
924.7915	Cer 62:11;4O|Cer 18:2;2O/44:9;2O	776.64	915.59	35.39	41.47	200.78	404.54	277.98	532.70
863.69226	CoQ10	517.15	642.19	2776.64	3608.78	356.20	295.69	335.95	464.30
395.27692	DG 18:0	139.80	60.59	41.42	52.67	142.47	120.26	65.35	96.49
409.27856	DG 19:0	112.19	62.63	49.02	40.09	293.53	288.87	48.43	37.32
437.31955	DG 21:0	44.67	37.80	157.15	411.53	29.78	18.83	22.44	3.90
435.30896	DG 21:1	113.23	90.74	51.49	35.42	67.30	45.82	57.29	13.15
465.34558	DG 23:0	302.28	146.21	114.54	75.67	1220.12	1307.25	156.76	169.81
463.32043	DG 23:1	54.13	37.22	10.61	11.05	52.76	115.33	165.23	316.80
461.32413	DG 23:2	370.55	467.39	31.90	31.39	671.49	1853.26	214.04	401.96
459.30319	DG 23:3	44.25	36.45	94.74	214.04	35.60	21.57	23.06	7.85
457.28665	DG 23:4	2191.77	2321.82	583.53	294.68	912.14	1053.69	931.32	243.65
479.37543	DG 24:0	260.82	239.71	78.33	102.98	101.43	72.14	67.38	34.40
475.3461	DG 24:2	144.90	157.58	42.68	25.36	48.01	40.38	39.84	7.66
473.31876	DG 24:3	12534.82	12594.37	29072.14	69859.84	4910.56	5777.60	5318.55	1264.45
471.29395	DG 24:4	141.09	138.24	37.10	15.25	44.31	26.87	49.09	10.10
491.36917	DG 25:1	50.65	21.40	39.97	28.66	114.98	77.11	85.03	65.24
487.33441	DG 25:3	1104.86	1113.03	7697.61	20630.14	460.12	441.32	450.85	108.61
485.31921	DG 25:4	1005.53	1034.21	346.01	284.74	376.50	453.70	420.53	117.71
503.37045	DG 26:2	420.82	324.15	30.50	25.77	65.37	51.10	469.64	898.30
501.35501	DG 26:3	6715.14	12216.44	95.76	110.69	731.89	1556.55	886.77	1009.04

**Mass** *m/z*	**Metabolite name**	**CTR**		**SCH**		**NF**		**PNF**	
		mean	*dev*	*mean*	*dev*	*mean*	*dev*	*mean*	*dev*
499.34137	DG 26:4	1421.55	1234.31	482.51	436.42	462.92	574.32	577.18	155.70
497.3237	DG 26:5	447.20	721.31	26.36	32.23	100.43	170.64	137.27	109.90
521.40369	DG 27:0	92.68	90.28	156.84	349.16	167.06	241.90	13.72	5.90
519.40436	DG 27:1	23.88	9.85	23.70	15.84	89.61	70.09	59.83	57.15
517.39435	DG 27:2	163.90	151.20	41.73	36.05	111.59	115.35	88.64	60.32
512.43097	DG 27:2 | DG 12:0_15:2	752.12	932.28	46.79	37.56	1139.20	3085.78	383.50	714.33
515.36621	DG 27:3	356.93	364.64	4797.90	13645.37	146.67	169.61	163.24	31.70
513.34796	DG 27:4	683.22	697.52	491.16	869.33	247.90	281.91	271.47	36.04
511.34354	DG 27:5	234.45	267.51	64.63	62.92	85.59	72.23	62.39	20.04
535.42731	DG 28:0	693.23	709.00	152.44	98.57	229.98	284.05	247.98	162.86
533.41638	DG 28:1	34857.88	36411.13	9124.66	5648.94	13122.51	17012.50	14428.43	8553.22
531.40906	DG 28:2	656026.44	682950.38	162590.10	101980.07	266593.82	344977.69	290423.34	164801.14
529.38989	DG 28:3	162.63	181.41	37.47	26.44	70.93	64.20	71.88	55.26
527.36572	DG 28:4	37.75	34.79	328.46	982.65	17.37	14.45	15.43	2.14
525.35687	DG 28:5	136.41	118.20	40.30	19.72	52.47	50.88	55.35	13.80
547.41608	DG 29:1	106.23	86.84	44.90	32.31	54.80	46.37	52.30	9.97
543.39838	DG 29:3	1377.52	1581.21	7442.94	18112.00	633.17	746.24	800.24	219.82
541.38739	DG 29:4	6626.12	11563.44	211.55	226.10	919.54	1813.20	1575.69	1464.94
563.45996	DG 30:0	18.78	13.33	90.55	245.80	23.18	15.17	13.38	4.52
554.48145	DG 30:2 | DG 14:1_16:1	54.47	43.16	0.60	0.64	57.66	161.92	205.68	410.31
555.39154	DG 30:4	1271.46	692.74	602.09	293.80	732.39	704.63	710.74	230.75
553.38953	DG 30:5	23513.41	10461.05	11589.14	5743.55	12541.36	13742.09	13961.48	5594.18
551.37482	DG 30:6	886.98	725.70	144.39	176.98	377.99	743.94	388.83	358.54
549.35803	DG 30:7	50.16	32.66	19.41	27.37	20.98	25.88	37.47	20.50
577.4845	DG 31:0	18.03	9.35	29.85	22.76	130.10	161.34	54.59	20.94
569.42664	DG 31:4	16.43	9.75	91.20	175.10	34.04	21.99	12.99	8.68

Mass *m/z*	Metabolite name	CTR		SCH		NF		PNF	
		mean	*dev*	*mean*	*dev*	*mean*	*dev*	*mean*	*dev*
567.41626	DG 31:5	21.37	11.59	53.75	90.62	34.91	15.53	14.13	4.81
565.3772	DG 31:6	853.08	984.61	204.66	288.69	42333.43	70305.73	1207.87	1564.99
563.39246	DG 31:7	37.07	14.93	49.37	63.32	65.46	34.44	27.61	15.93
591.49738	DG 32:0	24.64	20.02	2.96	1.94	11.35	18.55	348.55	693.89
589.46362	DG 32:1	159.38	108.91	69.26	38.80	83.46	63.83	78.30	28.21
584.52686	DG 32:1 | DG 16:0_16:1	4196.81	3986.35	92.55	64.65	3559.75	9448.67	10806.48	21454.31
582.50732	DG 32:2 | DG 16:1_16:1	85.13	66.79	8.14	5.05	193.50	535.60	752.24	1494.62
583.4281	DG 32:4	140.12	57.31	5916.38	14587.33	85.30	108.97	121.82	41.71
577.38379	DG 32:7	101.11	90.80	1509.02	4329.55	51.89	45.17	46.72	10.57
605.50366	DG 33:0	1057.42	654.87	429.05	204.82	577.01	636.03	581.26	186.09
598.53894	DG 33:1 | DG 15:0_18:1	68.41	36.56	29.19	16.07	178.02	232.71	173.99	280.17
601.49762	DG 33:2	85.32	42.40	42.62	30.00	80.12	39.76	36.49	8.42
591.39893	DG 33:7	68.47	54.60	624.56	1597.01	37.59	28.77	41.13	5.65
589.38843	DG 33:8	62.51	37.13	28.22	15.00	95.53	69.60	29.85	9.37
619.52905	DG 34:0	24.63	20.53	1.41	1.21	9.04	19.04	292.53	584.07
617.51697	DG 34:1	283.17	158.23	208.46	230.80	733.79	797.02	246.84	178.71
613.48816	DG 34:3	6.09	4.71	49.01	112.10	5.68	3.51	3.62	1.07
608.52698	DG 34:3 | DG 16:0_18:3	30.39	15.99	8.71	5.76	126.83	318.41	65.50	118.07
609.44397	DG 34:5	3.34	2.30	266.02	773.90	6.74	5.84	1.64	0.29
601.40436	DG 34:9	72.84	56.51	866.52	2451.13	41.22	43.99	41.02	7.83
624.55573	DG 35:2 | DG 17:0_18:2	62.49	42.93	16.09	7.40	112.88	161.83	80.26	118.26
627.48993	DG 35:3	48.18	25.03	665.68	1558.06	45.86	51.81	56.84	20.25
625.49292	DG 35:4	330.07	174.64	165.67	136.97	420.55	343.31	125.95	80.87
623.44891	DG 35:5	119.26	70.60	46.58	56.20	119.44	87.68	38.70	31.02
619.44348	DG 35:7	10.90	6.50	57.63	118.26	12.02	7.05	5.01	2.32

**Mass**	**Metabolite name**	**CTR**		**SCH**		**NF**		**PNF**	
m/z		*mean*	*dev*	*mean*	*dev*	*mean*	*dev*	*mean*	*dev*
645.54333	DG 36:1	267.21	229.88	229.78	261.90	279.94	136.31	129.17	117.10
638.56677	DG 36:2 I DG 18:1_18:1	7872.16	5528.99	2119.12	1730.98	12879.26	20238.31	5592.89	7338.26
634.53613	DG 36:4 I DG 16:0_20:4	3025.48	2137.81	1262.26	1206.92	6268.87	7383.30	1977.52	1712.86
641.40271	DG 37:10	75.35	33.17	36.90	13.18	198.10	171.20	51.48	19.96
652.5871	DG 37:2 I DG 18:1_19:1	25.93	18.95	10.69	6.19	36.27	39.21	16.20	6.29
653.50616	DG 37:4	9.28	6.19	39.75	90.41	22.57	17.82	7.47	2.94
648.55176	DG 37:4 I DG 17:0_20:4	92.57	66.35	53.76	41.36	167.76	144.91	45.54	17.89
647.46149	DG 37:7	19.21	9.15	416.48	1056.42	7.42	11.68	10.63	2.21
645.46466	DG 37:8	109.72	54.48	51.56	67.23	111.97	72.93	38.26	32.02
675.57935	DG 38:0	2929.66	3235.76	1831.45	2592.01	1253.65	805.36	1073.63	689.17
673.59668	DG 38:1	77.25	145.34	156.61	160.67	77.37	91.04	92.00	73.19
671.57129	DG 38:2	187.37	369.39	344.18	174.22	87.46	88.89	322.12	265.71
667.52118	DG 38:4	29.98	14.42	126.26	286.89	72.64	56.23	21.80	7.46
665.51367	DG 38:5	59.71	14.95	26.74	28.52	185.40	184.21	48.32	39.64
663.48547	DG 38:6	112.62	96.24	299.99	815.99	53.51	66.19	53.64	17.05
661.49231	DG 38:7	12.56	13.53	171.99	517.66	21.24	17.80	90.25	173.43
659.46442	DG 38:8	67.98	61.99	1095.81	2743.48	51.75	82.27	38.96	11.06
685.57153	DG 39:2	85.27	49.86	44.45	19.98	230.78	225.71	63.67	23.47
679.52087	DG 39:5	38.41	40.20	55.86	94.91	36.40	27.98	8.21	2.29
681.45923	DG 40:11	90.48	67.74	33.00	23.63	37.00	35.20	32.36	2.53
695.56042	DG 40:4	10.51	7.99	23.98	41.06	13.90	8.08	5.76	3.74
693.54126	DG 40:5	23.82	21.51	11.09	9.99	13.33	7.41	5.56	4.06
691.52814	DG 40:6	14.24	11.22	35.49	76.83	9.23	5.92	3.43	1.96
686.56677	DG 40:6 I DG 16:0_24:6	0.84	0.54	9.86	22.55	2.97	2.40	0.56	0.12
686.57452	DG 40:6 I DG 18:1_22:5	124.85	71.26	39.99	27.44	164.51	135.58	49.66	24.54
689.49023	DG 40:7	97.94	57.69	43.34	58.49	88.55	54.02	35.72	34.49

Mass m/z	Metabolite name	CTR		SCH		NF		PNF	
		mean	dev	mean	dev	mean	dev	mean	dev
685.46753	DG 40:9	69.74	61.92	268.25	778.43	54.50	31.70	44.92	33.93
695.46448	DG 41:11	12.62	8.10	42.07	80.71	19.84	17.30	8.03	2.85
711.59216	DG 41:3	141.77	184.13	252.29	340.77	215.08	212.99	105.99	61.76
709.57281	DG 41:4	370.32	204.57	187.22	114.85	679.61	546.51	278.57	169.66
705.52997	DG 41:6	183.55	147.17	105.17	63.29	203.00	130.33	119.11	56.72
703.50537	DG 41:7	176.29	148.15	89.70	141.70	180.05	164.70	58.23	50.85
701.51776	DG 41:8	1.98	1.56	21.87	40.02	8.14	14.70	3.46	2.63
699.49799	DG 41:9	7.46	4.47	13.76	15.43	11.97	10.56	6.50	3.22
722.67108	DG 42:2丨DG 18:1_24:1	16.32	10.21	17.10	8.46	13.27	9.25	19.76	24.23
719.54993	DG 42:6	90.82	56.39	46.58	24.99	205.11	173.64	59.04	23.58
715.51917	DG 42:8	29.23	26.62	37.59	58.39	35.17	24.78	76.49	82.49
713.50067	DG 42:9	1087.99	1018.16	607.68	501.80	1086.42	727.63	567.28	317.47
745.66528	DG 43:0	48.08	44.52	68.04	154.06	193.46	246.68	18.82	7.02
725.51807	DG 43:10	168.64	147.20	81.26	130.29	116.12	84.44	49.40	45.21
735.56744	DG 43:5	28.75	31.09	19.58	26.63	1177.28	3592.89	17.08	6.15
731.54089	DG 43:7	2154.44	1595.39	4864.80	4760.96	7003.08	5559.20	2543.38	1271.20
727.5238	DG 43:9	53.59	43.90	123.32	156.71	176.05	182.17	43.55	30.87
757.66077	DG 44:1	110.37	75.81	153.81	329.27	183.47	197.87	42.91	25.06
739.52045	DG 44:10	1056.87	1144.94	626.67	670.52	658.09	288.29	468.93	280.35
737.51324	DG 44:11	50.54	25.85	193.74	225.61	285.73	300.12	134.71	121.34
735.49188	DG 44:12	3.58	3.11	88.44	283.67	2.72	2.31	1.31	0.55
753.63483	DG 44:3	15.39	12.35	461.95	1212.80	7.45	9.84	8.94	5.83
747.5899	DG 44:6	31.91	39.26	111.86	273.47	37.37	30.71	6.67	1.47
743.54974	DG 44:8	24.04	7.96	54.42	47.59	186.04	261.68	27.67	10.26
741.55023	DG 44:9	692.84	913.44	290.66	348.07	1331.06	2009.89	464.73	338.70
753.5343	DG 45:10	106.93	70.76	165.77	140.36	269.83	339.24	109.40	70.75
749.51227	DG 45:12	16.34	8.53	35.52	46.28	22.02	18.92	13.87	7.08

**Mass** *m/z*	**Metabolite name**	**CTR**		**SCH**		**NF**		**PNF**	
		mean	*dev*	*mean*	*dev*	*mean*	*dev*	*mean*	*dev*
757.56409	DG 45:8	8645.78	5408.90	9328.74	7183.15	18136.32	10046.38	8803.69	4377.69
755.56213	DG 45:9	15.08	11.85	26.75	28.68	51.31	88.75	10.89	4.38
767.5426	DG 46:10	63.32	23.68	52.53	58.02	226.30	264.68	25.32	5.84
765.53802	DG 46:11	248.10	131.35	135.33	87.38	421.98	276.61	113.10	44.11
763.51453	DG 46:12	124.59	59.65	136.46	100.49	270.51	180.80	116.75	81.98
771.57635	DG 46:8	175.74	77.43	248.22	227.19	1121.67	1457.61	215.43	78.18
769.58472	DG 46:9	52.92	50.77	35.72	37.35	146.26	250.78	37.38	19.67
781.5639	DG 47:10	34.43	19.37	60.88	54.13	62.44	60.04	23.48	10.54
779.55676	DG 47:11	141.15	106.30	93.41	79.09	165.50	97.32	60.57	23.31
775.547	DG 47:13	32.46	13.99	29.95	26.56	70.73	53.13	26.34	15.20
791.53046	DG 48:12	225.63	199.25	116.37	84.22	220.06	94.11	105.24	57.95
803.5448	DG 49:13	35.99	32.45	48.45	72.70	54.13	43.27	24.97	8.38
823.6059	DG 50:10	81.63	18.34	53.18	36.76	312.99	350.89	66.90	16.19
857.79816	DG 51:0	4.89	4.31	7.14	11.79	93.14	272.88	403.20	464.16
833.58264	DG 51:12	544.80	244.65	419.79	324.94	1112.23	559.82	233.63	100.23
841.66754	DG 51:8	60.86	52.09	37.36	27.84	554.39	986.13	54.82	19.85
845.62598	DG 52:13	101.57	71.12	36.04	15.40	115.09	71.78	41.22	14.25
642.53967	DG O-38:7 I DG O-16:1_22:6	71.20	80.59	85.04	55.72	110.36	79.30	38.28	38.24
834.60138	Hex2Cer 32:1;2O I Hex2Cer 18:1;2O/14:0	56.02	52.58	65.74	153.07	46.81	35.44	15.07	2.60
848.6142	Hex2Cer 33:1;2O I Hex2Cer 17:1;2O/16:0	123.09	111.42	67.77	89.71	163.25	126.56	37.00	19.80
862.62891	Hex2Cer 34:1;2O I Hex2Cer 18:1;2O/16:0	1559.06	2282.33	273.98	287.00	635.45	324.96	140.25	56.68
1024.67993	Hex3Cer 34:1;2O I Hex3Cer 18:1;2O/16:0	1080.90	1843.59	48.32	64.63	191.44	155.38	86.37	92.90
644.50946	HexCer 30:1;2O I HexCer 18:1;2O/12:0	14.12	10.72	721.24	2233.13	16.70	12.23	6.79	2.52
674.55328	HexCer 32:0;2O	12.75	7.57	28.00	49.44	14.56	8.13	16.73	12.88
672.54266	HexCer 32:1;2O I HexCer 18:1;2O/14:0	15.98	10.54	52.77	97.27	19.29	12.49	8.11	2.37
686.55945	HexCer 33:1;2O I HexCer 17:1;2O/16:0	23.72	14.56	54.18	75.77	38.06	21.98	18.99	9.21
702.58508	HexCer 34:0;2O I HexCer 18:0;2O/16:0	219.06	139.30	624.45	1111.75	428.79	430.78	107.10	48.92

Mass m/z	Metabolite name	CTR		SCH		NF		PNF	
		mean	dev	mean	dev	mean	dev	mean	dev
700.57001	HexCer 34:1;2O∣HexCer 18:1;2O/16:0	145.24	87.76	1183.51	2378.21	247.41	207.81	75.06	37.41
716.57648	HexCer 34:1;3O∣HexCer 16:1;2O/18:0;O	83.85	52.64	74.97	40.39	249.00	210.47	66.51	22.62
716.56763	HexCer 34:1;3O∣HexCer 18:1;2O/16:0;O	28.04	23.47	75.42	148.96	32.60	19.25	14.34	4.35
698.55591	HexCer 34:2;2O∣HexCer 18:2;2O/16:0	287.36	316.77	4647.78	8477.75	299.70	300.36	119.75	120.33
696.53522	HexCer 34:2;3O∣HexCer 17:1;2O/17:1;O	10.64	8.04	25.97	53.20	15.92	12.23	16.56	11.87
714.58069	HexCer 35:1;2O∣HexCer 17:1;2O/18:0	59.24	84.96	100.26	160.70	50.42	40.52	45.09	28.75
726.56506	HexCer 35:3;3O∣HexCer 18:2;2O/17:1;O	36.87	53.70	111.10	184.13	25.52	16.45	26.66	20.41
728.60583	HexCer 36:1;2O∣HexCer 18:1;2O/18:0	274.77	429.47	572.50	816.25	338.29	345.47	235.90	163.50
726.58563	HexCer 36:2;2O∣HexCer 18:2;2O/18:0	211.03	341.57	1342.59	2764.15	123.22	104.09	112.14	82.25
736.58295	HexCer 37:3;3O∣HexCer 18:2;2O/19:1;O	142.16	93.60	93.61	44.20	363.56	269.86	113.38	53.27
732.54559	HexCer 37:5;3O	406.20	259.07	913.00	903.32	1433.79	1146.59	505.47	224.86
756.63287	HexCer 38:1;2O∣HexCer 18:1;2O/20:0	189.91	236.31	192.76	328.63	450.44	645.77	301.39	370.97
746.56274	HexCer 38:5;3O	47.54	33.61	67.25	87.04	84.15	67.83	56.26	23.13
760.55713	HexCer 39:5;3O	212.50	252.87	87.15	51.34	262.34	117.28	115.29	43.94
796.651	HexCer 41:1;3O∣HexCer 18:1;2O/23:0;O	655.39	420.52	353.52	260.84	1601.08	1529.05	367.71	89.21
269.08798	Inosine	122.90	174.74	137.13	214.44	248.87	293.63	82.35	41.61
468.30945	LPC 14:0	355.24	346.74	344.12	263.13	224.11	160.37	180.38	80.22
482.32275	LPC 15:0	150.49	140.58	135.22	67.82	217.78	332.03	63.60	27.87
482.31622	LPC 15:0/0:0	290.76	295.33	153.87	277.00	71.64	38.24	44.09	27.23
480.31396	LPC 15:1/0:0	50.51	35.05	636.22	1722.05	63.75	41.53	48.83	54.49
476.27042	LPC 15:3	5800.06	4367.32	2194.88	3305.91	3585.59	8154.12	5554.47	4630.84
496.34119	LPC 16:0/0:0	45.30	46.69	628.92	1507.47	42.04	34.36	14.32	2.34
494.31833	LPC 16:1	3232.47	4361.05	2026.68	1160.60	2001.77	3469.00	987.13	471.26
494.32764	LPC 16:1/0:0	38.68	59.70	9535.45	29294.79	26.16	25.31	6.24	1.88
488.27655	LPC 16:4/0:0	12.51	4.35	998.28	2545.97	33.54	26.05	8.78	4.79

**Mass**	**Metabolite name**	**CTR**		**SCH**		**NF**		**PNF**	
m/z		*mean*	*dev*	*mean*	*dev*	*mean*	*dev*	*mean*	*dev*
510.35226	LPC 17:0	61.67	55.09	96.37	140.27	82.71	74.70	26.29	11.74
510.35229	LPC 17:0/0:0	742.41	1096.15	151.14	134.91	503.93	1039.58	136.20	60.54
508.34341	LPC 17:1	54.66	46.31	90.32	65.57	209.97	436.35	25.49	10.29
508.33676	LPC 17:1/0:0	148.92	142.91	116.32	59.75	208.95	276.38	65.90	35.66
524.37256	LPC 18:0	10679.44	11020.16	5891.67	5542.29	8860.81	9040.81	6262.94	4141.87
524.36725	LPC 18:0/0:0	9.79	6.65	1650.11	3874.51	28.52	20.40	9.48	2.69
522.35333	LPC 18:1	103.14	126.21	85.75	83.64	132.87	172.04	31.97	16.11
522.35913	LPC 18:1/0:0	91.36	103.46	251.51	396.67	86.34	65.03	46.79	29.10
520.33148	LPC 18:2	404.77	274.86	75.67	74.32	643.14	727.49	3219.58	6339.83
518.32281	LPC 18:3	691.77	674.19	1087.94	883.02	582.18	492.56	267.91	176.95
516.31165	LPC 18:4/0:0	158.70	197.48	80.40	35.96	92.71	79.46	37.37	11.94
538.39056	LPC 19:0	114.34	126.27	42.15	24.48	93.84	68.49	39.44	19.27
538.3877	LPC 19:0/0:0	414.33	450.93	175.20	124.01	287.10	257.80	171.34	104.24
528.31519	LPC 19:5	62.08	37.06	57.19	43.30	41.62	14.31	20.28	7.81
552.40033	LPC 20:0	760.27	725.92	246.27	232.68	926.39	1247.94	428.21	358.94
550.38251	LPC 20:1	2814.59	3348.47	664.03	516.96	981.17	839.03	832.78	867.68
550.38568	LPC 20:1/0:0	6500.63	8034.41	1562.43	1377.58	2934.85	2948.69	2219.46	2160.58
548.36823	LPC 20:2	3122.08	5155.00	611.98	543.42	762.98	723.76	497.38	465.59
546.35449	LPC 20:3/0:0	409.25	388.70	756.19	586.35	589.15	406.97	282.80	172.95
544.34241	LPC 20:4	8219.33	10355.18	10467.78	9475.95	7152.04	5515.83	4570.11	2583.25
542.32086	LPC 20:5	58.96	64.26	77.75	83.42	70.44	46.77	44.47	41.63
560.37024	LPC 21:3	89.85	107.62	42.44	42.40	54.35	41.68	24.88	10.13
580.43494	LPC 22:0/0:0	126.09	113.24	68.50	60.48	245.06	302.66	89.83	47.10
578.41772	LPC 22:1	294.19	272.89	98.92	71.32	221.79	159.50	164.31	135.24
576.4068	LPC 22:2	122.93	125.00	60.04	43.52	69.72	43.32	51.64	35.36
574.38586	LPC 22:3	122.50	153.19	46.32	29.50	52.74	21.80	29.56	13.45

**Mass** *m/z*	**Metabolite name**	**CTR**		**SCH**		**NF**		**PNF**	
		mean	*dev*	*mean*	*dev*	*mean*	*dev*	*mean*	*dev*
572.37372	LPC 22:4	272.25	350.62	156.41	116.76	105.99	68.05	58.74	41.89
570.35419	LPC 22:5	139.53	164.92	189.99	184.71	112.91	91.29	61.29	62.40
570.3576	LPC 22:5/0:0	165.43	209.84	201.64	197.47	103.66	72.33	62.52	65.95
568.34088	LPC 22:6	1000.26	1319.87	1228.37	968.80	876.56	737.58	466.20	294.54
608.46637	LPC 24:0/0:0	346.73	325.44	219.39	203.86	838.06	1457.97	230.89	133.33
606.45166	LPC 24:1	260.94	293.97	121.17	102.81	307.66	329.11	189.91	126.76
604.44269	LPC 24:2	30.01	25.77	24.24	14.68	30.14	17.88	25.23	15.07
600.40131	LPC 24:4/0:0	94.91	85.97	34.38	20.12	127.69	147.27	44.45	30.10
622.48212	LPC 25:0/0:0	70.60	95.13	24.33	18.19	75.32	108.33	23.04	14.58
636.49536	LPC 26:0	46.28	35.97	24.03	17.65	66.48	63.93	25.66	14.87
636.49683	LPC 26:0/0:0	222.29	287.26	93.38	134.35	329.02	489.15	100.44	66.69
634.47858	LPC 26:1/0:0	245.03	325.48	121.60	97.08	184.81	184.91	137.82	87.36
632.47113	LPC 26:2	33.01	19.35	35.37	31.37	35.18	25.12	27.90	18.88
624.38708	LPC 26:6	92.73	49.32	21.32	27.27	68.59	63.23	22.79	21.94
622.40265	LPC 26:7	644.50	740.49	73.73	46.13	214.77	226.69	154.92	90.12
664.53003	LPC 28:0	643.62	719.19	143.78	98.23	1142.86	2182.14	327.73	294.14
662.51178	LPC 28:1	119.71	125.01	64.68	46.94	201.55	168.32	91.44	83.88
652.44135	LPC 28:6	192.53	128.37	51.33	34.05	99.67	89.27	69.55	13.23
650.40057	LPC 28:7	256.54	345.53	29.47	16.55	80.43	53.03	55.79	20.18
454.32968	LPC O-14:0	47.11	31.99	2974.86	7341.56	36.43	31.03	18.68	5.35
496.37567	LPC O-17:0	140.27	117.31	6546.08	17170.68	124.04	76.25	50.32	34.38
494.35095	LPC O-17:1	258.80	416.95	5872.73	15207.37	101.00	81.42	32.42	17.40
504.34448	LPC O-18:3	119.09	168.66	4609.65	10711.31	81.33	64.70	25.34	8.56
502.32233	LPC O-18:4	321.84	432.83	407.92	780.62	216.70	165.08	69.03	34.97
538.42609	LPC O-20:0	105.65	80.55	3924.49	10968.16	138.43	97.10	61.62	31.22
534.39014	LPC O-20:2	73.43	85.01	3161.36	9236.98	78.36	46.19	35.42	16.05

**Mass** *m/z*	**Metabolite name**	**CTR**		**SCH**		**NF**		**PNF**	
		mean	*dev*	*mean*	*dev*	*mean*	*dev*	*mean*	*dev*
564.44	LPC O-22:1	58.26	58.23	253.50	672.00	40.70	26.91	24.84	6.48
588.44183	LPC O-24:3	27.08	16.73	972.47	2891.54	89.48	133.26	20.02	14.67
614.45856	LPC O-26:4	7.68	3.81	320.07	738.92	7.51	4.62	3.97	1.14
454.29282	LPE 16:0	557.95	712.50	117.69	76.99	172.87	107.77	110.84	102.24
480.31662	LPE 18:1	15559.37	17413.79	2226.16	2532.61	1804.51	1679.95	1238.24	857.78
478.29202	LPE 18:2	184.26	173.38	96.90	94.64	231.04	271.90	70.87	34.02
510.3577	LPE 20:0	426.70	570.94	16.73	12.44	74.40	108.47	41.08	36.86
508.34116	LPE 20:1	1620.52	1823.09	98.64	84.34	188.20	130.72	287.42	330.45
502.2941	LPE 20:4	2342.98	1769.75	1871.76	2576.17	1978.04	2396.23	967.08	723.02
538.38885	LPE 22:0	185.46	293.20	6.05	4.11	35.78	89.32	10.73	10.58
536.37012	LPE 22:1	253.45	278.29	17.05	9.60	44.73	33.49	39.18	41.12
530.32751	LPE 22:4	180.02	118.37	188.43	273.66	170.31	161.77	70.41	24.44
528.30994	LPE 22:5	96.57	57.19	85.56	77.03	83.72	48.52	31.62	15.39
526.29205	LPE 22:6	984.05	824.07	511.53	706.70	707.69	958.96	248.54	255.13
564.3999	LPE 24:1	224.64	250.63	18.53	16.69	41.54	47.14	23.38	18.92
558.35437	LPE 24:4	73.50	114.88	18.04	20.95	40.83	36.46	30.99	30.82
424.28323	LPE O-15:1	48.82	39.86	1111.10	2789.98	37.99	29.25	12.33	6.16
440.31055	LPE O-16:0	917.71	951.37	4492.83	10811.88	232.62	172.82	113.96	103.65
438.29813	LPE O-16:1	24.46	26.76	172.66	503.61	19.84	13.34	8.18	3.09
436.28104	LPE O-16:2	42.58	29.99	3071.51	7367.66	76.18	74.99	18.95	4.01
452.31323	LPE O-17:1	443.73	498.94	15499.00	41887.11	413.67	454.58	61.55	26.92
450.29495	LPE O-17:2	77.36	46.78	633.49	1421.62	139.10	145.06	38.33	14.43
468.33948	LPE O-18:0	417.52	477.16	2374.84	5698.31	108.98	82.74	41.78	35.42
466.33023	LPE O-18:1	22.18	23.44	233.11	707.52	16.35	11.38	5.67	1.96
464.31583	LPE O-18:2	531.94	828.89	651.96	1820.39	170.31	150.27	35.65	5.61
462.2962	LPE O-18:3	84.27	83.70	1787.99	4211.05	41.78	20.86	22.88	6.23
480.34451	LPE O-19:1	40.94	47.56	1307.97	4105.29	23.95	16.77	8.69	3.11

**Mass** *m/z*	**Metabolite name**	**CTR**		**SCH**		**NF**		**PNF**	
		mean	*dev*	*mean*	*dev*	*mean*	*dev*	*mean*	*dev*
494.36292	LPE O-20:1	86.52	104.74	5101.76	14994.16	98.25	96.42	16.79	7.22
492.34756	LPE O-20:2	247.81	362.94	6734.66	16616.96	86.11	66.59	28.78	19.32
506.35855	LPE O-21:2	27.10	27.64	203.21	484.47	16.87	9.87	7.27	1.30
522.39246	LPE O-22:1	22.81	23.93	1187.68	3588.55	24.53	21.93	5.79	0.99
520.37695	LPE O-22:2	39.89	46.71	5403.50	13955.38	37.00	28.22	10.40	2.88
518.36121	LPE O-22:3	25.03	20.87	1107.74	3201.90	14.94	8.75	9.79	2.05
516.34271	LPE O-22:4	14.94	11.87	202.49	571.23	17.75	11.70	7.12	2.90
550.41278	LPE O-24:1	10.63	7.96	178.63	454.17	9.64	6.05	3.70	0.87
548.40808	LPE O-24:2	61.02	63.52	4616.45	11808.05	52.43	36.87	16.83	3.16
546.39972	LPE O-24:3	2182.90	2243.46	3446.53	7528.28	878.80	1010.09	906.84	222.65
544.37262	LPE O-24:4	19.09	18.20	460.78	1122.18	20.90	15.81	7.62	1.40
574.4256	LPE O-26:3	62.23	68.12	55.02	123.41	27.07	18.76	20.94	3.93
572.41901	LPE O-26:4	80.46	81.22	49.59	77.37	45.66	33.48	38.36	5.24
348.31238	MG 16:0	68.26	51.24	52.33	44.15	264.95	269.62	57.71	41.38
342.285	MG 16:3	153.03	150.72	79.06	83.11	113.88	65.79	55.07	31.52
376.34103	MG 18:0	172.69	207.04	352.39	793.32	243.11	199.56	43.56	6.45
368.28043	MG 18:4	138.93	61.71	118.29	108.82	488.84	672.91	69.52	42.34
404.38086	MG 20:0	25.49	15.13	22.73	20.00	40.70	25.53	16.94	9.97
414.35855	MG 21:2	129.89	101.33	11.81	5.92	101.85	185.58	36.09	56.93
472.43539	MG 25:1	193.24	332.81	24.88	20.30	490.69	1115.36	12.89	2.55
480.40381	MG 26:4	10.32	7.53	152.14	446.60	10.08	10.30	6.09	5.33
612.50287	MG 36:8	110.33	178.09	36.04	65.13	77.18	81.25	20.06	32.37
258.2403	NAE 13:0	111.99	147.70	20.74	12.39	49.32	34.49	33.17	18.45
256.22607	NAE 13:1	121.82	119.23	82.87	47.58	207.95	202.78	47.95	34.73
270.24222	NAE 14:1	550.77	547.80	401.71	285.30	819.97	761.67	205.00	174.79
286.27179	NAE 15:0	50.96	48.43	35.38	46.65	112.62	113.26	18.91	4.07
278.20987	NAE 15:4	49.49	47.22	64.87	112.40	32.62	25.68	23.15	11.01

**Mass** *m/z*	**Metabolite name**	**CTR**		**SCH**		**NF**		**PNF**	
		mean	*dev*	*mean*	*dev*	*mean*	*dev*	*mean*	*dev*
300.28815	NAE 16:0	127.94	107.13	109.04	131.61	653.30	536.42	79.71	50.83
298.27588	NAE 16:1	128.52	103.50	74.18	52.13	156.52	93.11	50.71	13.65
314.30579	NAE 17:0	14.88	13.80	813.01	1885.97	10.36	9.74	2.20	1.13
312.29086	NAE 17:1	26.77	22.85	467.95	1344.87	16.78	16.11	5.55	1.77
328.32111	NAE 18:0	492.88	468.47	271.38	339.04	1200.55	2427.17	142.17	76.14
326.30566	NAE 18:1	172.53	100.63	274.59	279.62	1450.38	3563.91	74.77	41.99
324.29074	NAE 18:2	106.46	140.81	108.75	170.87	44.54	21.46	18.85	11.92
322.27328	NAE 18:3	1520.11	2067.95	882.22	848.94	610.18	353.49	212.62	128.87
320.25903	NAE 18:4	25.32	24.23	28.13	40.42	39.81	28.75	9.87	5.69
342.33621	NAE 19:0	455.18	444.31	216.70	175.63	390.22	439.59	241.55	149.68
340.32196	NAE 19:1	10.34	6.67	188.98	476.31	12.47	9.29	3.81	0.88
338.31052	NAE 19:2	32.55	35.81	1420.77	3524.94	31.96	26.46	8.59	2.91
334.27518	NAE 19:4	13.04	16.17	94.37	166.82	15.41	17.70	2.34	0.55
332.25458	NAE 19:5	189.01	299.66	17.89	11.42	72.37	51.84	21.71	19.40
354.33762	NAE 20:1	164.12	105.72	129.88	87.57	3679.02	6010.64	113.33	31.56
352.32364	NAE 20:2	61.74	53.53	39.33	16.08	91.91	59.68	32.29	17.76
350.30582	NAE 20:3	390.29	485.51	989.39	2316.73	76.86	51.37	45.02	21.60
348.28937	NAE 20:4	259.88	310.15	214.97	186.75	130.30	87.53	45.72	49.06
370.36798	NAE 21:0	44.03	35.30	58.30	57.42	76.76	48.78	27.54	12.28
368.35175	NAE 21:1	46.05	22.46	89.33	183.56	52.72	68.67	19.54	17.45
366.33844	NAE 21:2	12.49	7.44	651.31	2045.70	20.56	20.60	4.16	1.21
364.32074	NAE 21:3	83.89	82.04	4590.28	10541.84	21.07	9.81	8.32	5.32
362.30667	NAE 21:4	72.51	90.65	6534.29	15507.46	20.38	14.71	5.66	2.38
360.29111	NAE 21:5	175.97	217.74	22266.19	55913.08	74.65	53.81	21.14	11.09
384.39246	NAE 22:0	64.64	39.71	149.46	215.30	37.26	25.72	44.79	51.30
380.3364	NAE 22:2	178.54	104.78	101.68	155.95	575.92	439.57	920.08	1175.85

Mass *m/z*	Metabolite name	CTR		SCH		NF		PNF	
		mean	*dev*	*mean*	*dev*	*mean*	*dev*	*mean*	*dev*
374.31235	NAE 22:5	2654.30	2884.20	1668.49	1586.81	1307.51	1522.04	775.57	552.57
398.39868	NAE 23:0	264.99	174.28	111.34	70.60	196.84	199.84	91.97	13.83
396.38684	NAE 23:1	16.09	17.27	33.84	80.56	12.04	5.73	9.66	14.86
412.41681	NAE 24:0	96.04	82.43	25.19	27.25	260.77	416.18	98.43	122.72
404.33646	NAE 24:4	1173.14	2229.41	35.67	30.75	429.51	1136.94	30.62	18.54
400.31235	NAE 24:6	422.99	262.24	107.85	63.37	229.92	140.52	128.04	60.72
426.4292	NAE 25:0	208.48	106.33	96.30	69.69	146.53	128.36	73.11	7.65
438.42795	NAE 26:1	39.51	53.84	18.56	28.78	27.64	26.73	35.94	51.53
436.41138	NAE 26:2	9.52	7.46	6.11	6.27	3352.43	10429.23	8.46	2.70
434.40137	NAE 26:3	4.50	3.74	3.63	5.14	125.84	377.44	2.30	1.10
428.35379	NAE 26:6	1594.83	2175.93	6366.46	17159.32	440.21	486.99	217.74	39.84
454.45306	NAE 27:0	32.10	14.89	14.23	12.46	35.74	41.26	15.95	9.20
458.38199	NAE 28:5	94.74	73.55	37.48	20.42	80.32	117.27	63.46	61.81
456.36667	NAE 28:6	2112.34	2708.14	97.32	106.96	3727.96	10756.14	1257.96	2449.09
454.37354	NAE 28:7	123.84	114.95	90.42	124.37	644.83	1139.87	39.27	12.92
379.26273	NAOrn 16:4;O	114.21	136.49	141.94	270.43	243.08	229.06	140.01	154.80
496.30579	PC 15:0	79.38	88.12	388.51	1113.71	38.70	19.38	22.38	10.88
538.34869	PC 18:0	73.17	93.83	382.97	952.66	80.27	112.36	18.62	14.74
552.3689	PC 19:0	15.04	9.83	29.29	58.88	17.00	9.28	6.08	2.43
564.36554	PC 20:1	49.62	37.14	173.10	358.02	75.01	46.14	30.38	16.28
580.4007	PC 21:0	13.40	8.02	29.03	57.76	26.62	20.14	9.50	6.50
578.38104	PC 21:1	190.44	296.69	16.76	7.82	37.08	29.24	64.27	59.34
574.35181	PC 21:3	154.88	150.64	35.38	27.38	94.76	61.75	56.95	33.01
572.33295	PC 21:4	297.12	412.80	43.01	32.19	33.81	25.16	63.72	36.14
594.4173	PC 22:0	8.88	5.75	14.04	20.62	17.95	11.93	3.81	1.60
606.41168	PC 23:1	25.68	24.61	35.07	80.76	20.79	12.43	10.34	5.13
600.36609	PC 23:4	359.33	411.16	82.92	82.09	94.56	72.34	62.54	30.11

**Mass** *m/z*	**Metabolite name**	**CTR**		**SCH**		**NF**		**PNF**	
		mean	*dev*	*mean*	*dev*	*mean*	*dev*	*mean*	*dev*
622.44684	PC 24:0	28.05	22.91	21.86	24.22	37.33	14.78	9.50	5.93
614.38336	PC 24:4	529.79	572.91	77.67	56.75	277.11	150.73	74.29	36.93
636.45929	PC 25:0	112.33	89.09	33.29	18.49	44.22	36.04	35.63	21.59
650.47723	PC 26:0	62.83	88.98	26.09	23.28	60.97	48.81	54.69	48.32
650.47729	PC 26:0 | PC 10:0_16:0	68.69	59.80	55.42	60.96	124.76	86.12	46.84	21.13
648.46161	PC 26:1	12.45	6.94	17.84	30.91	18.19	11.35	6.86	3.48
678.50934	PC 28:0	20.20	13.12	7210.25	17378.27	21.45	14.63	15.79	13.67
678.50677	PC 28:0 | PC 14:0_14:0	6228.69	9407.41	3224.18	3379.89	4345.06	3600.14	5600.88	4720.62
676.48181	PC 28:1	102.85	80.26	60.44	60.56	89.24	97.47	47.19	16.77
674.46674	PC 28:2	57.81	55.06	226.36	419.19	112.50	156.24	45.98	37.80
670.44434	PC 28:4	12.09	10.74	13.56	20.90	7.51	4.74	2.51	1.48
692.52307	PC 29:0 | PC 14:0_15:0	7624.68	9513.90	4512.16	3431.96	12324.14	12043.71	6478.10	4142.41
688.49109	PC 29:2	10.63	5.10	332.89	1012.51	17.11	14.18	6.36	4.42
706.54016	PC 30:0 | PC 14:0_16:0	97510.60	69200.66	77405.81	48468.10	242286.08	189757.08	105195.24	48727.88
706.53986	PC 30:0 | PC 15:0_15:0	114.62	110.57	92.25	63.17	885.25	2085.58	123.12	58.84
704.5304	PC 30:1	477.33	381.32	277.96	178.22	530.89	351.10	318.25	174.45
704.52515	PC 30:1 | PC 14:0_16:1	2572.84	3336.72	3306.60	3438.04	3480.30	1926.63	5185.04	3404.63
702.51263	PC 30:2	42.53	48.68	42.06	43.51	58.34	41.16	51.30	37.88
702.5083	PC 30:2 | PC 14:0_16:2	28.70	29.00	35.58	37.00	64.88	89.70	30.41	20.23
698.45905	PC 30:4 | PC 15:2_15:2	159.73	178.15	80.64	189.84	34.08	61.29	175.40	271.69
720.56665	PC 31:0	174.65	244.63	59.50	83.63	799.60	1726.90	139.65	118.62
720.56879	PC 31:0 | PC 15:0_16:0	4348.02	3407.09	1101.12	1248.49	1918.82	2001.32	512.47	515.04
718.54565	PC 31:1	233.35	156.17	106.58	59.30	460.67	354.56	135.01	39.36
718.53973	PC 31:1 | PC 15:0_16:1	2761.40	3166.10	2380.30	2041.60	10549.52	20713.74	3941.25	2221.48
716.52673	PC 31:2	214.27	134.25	108.79	113.32	247.46	299.16	103.61	63.25
716.5238	PC 31:2 | PC 16:0_15:2	18.56	12.06	13.72	10.30	274.67	796.00	11.41	4.79
714.50183	PC 31:3	222.13	232.58	208.16	222.93	315.45	390.27	183.87	162.58

Mass *m/z*	Metabolite name	CTR		SCH		NF		PNF	
		mean	*dev*	*mean*	*dev*	*mean*	*dev*	*mean*	*dev*
734.56946	PC 32:0 I PC 16:0_16:0	125.02	102.34	66.84	53.60	265.93	346.57	162.98	162.52
730.53906	PC 32:2 I PC 16:0_16:2	6663.17	4834.63	14557.88	13822.66	24964.46	18525.17	8624.96	4581.95
730.54016	PC 32:2 I PC 16:1_16:1	15667.42	9767.57	20865.15	15842.97	41444.26	28016.40	17229.82	6159.24
728.52313	PC 32:3	208.29	178.57	239.20	275.88	150.82	92.96	64.35	45.61
728.5235	PC 32:3 I PC 14:0_18:3	46.49	31.87	56.32	50.81	105.92	134.77	41.40	31.41
728.52509	PC 32:3 I PC 16:0_16:3	204.09	173.19	244.36	283.80	142.89	85.72	62.29	38.66
726.51245	PC 32:4	59.30	64.23	91.12	106.64	68.13	37.13	68.67	69.55
726.49146	PC 32:4 I PC 16:2_16:2	50.88	55.77	27.15	49.80	27.45	44.86	66.26	98.82
746.5871	PC 33:1	70.24	45.82	80.77	80.67	120.68	82.96	58.56	21.70
744.55688	PC 33:2	242.25	189.40	323.82	246.62	973.69	1002.80	506.28	256.86
744.55157	PC 33:2 I PC 15:0_18:2	454.89	224.24	713.81	485.54	3952.94	4686.67	814.33	343.35
742.53998	PC 33:3	35.43	16.76	109.99	166.04	114.61	96.52	38.19	13.76
738.51715	PC 33:5	155.36	94.74	150.81	101.06	240.01	145.75	90.93	38.65
760.59106	PC 34:1 I PC 16:0_18:1	195.29	93.56	154.04	74.84	351.57	152.96	172.30	82.66
758.5589	PC 34:2	1806.37	1042.08	2299.38	1647.92	4507.73	2045.98	2876.87	1335.26
756.55237	PC 34:3	14.98	10.14	16.84	13.52	117.67	282.34	10.60	4.93
756.55719	PC 34:3 I PC 16:0_18:3	1160.48	406.74	1271.92	946.05	4333.23	3579.69	1567.36	1549.01
756.55029	PC 34:3 I PC 16:1_18:2	27053.39	16963.59	33578.70	25271.47	72891.53	34361.11	42297.72	20811.29
754.54596	PC 34:4	58.62	38.84	98.86	81.21	119.33	79.88	65.82	56.33
754.54333	PC 34:4 I PC 14:0_20:4	5421.16	5391.45	4391.09	3690.61	3932.77	2189.05	2973.10	1432.54
754.53772	PC 34:4 I PC 16:2_18:2	35.91	22.67	151.26	207.98	223.51	335.59	45.30	18.71
752.52228	PC 34:5	209.91	147.81	450.64	444.11	739.72	1071.54	259.35	170.23
752.52661	PC 34:5 I PC 16:0_18:5	252.59	181.53	466.64	429.85	735.39	1130.01	306.12	200.70
772.5777	PC 35:2 I PC 17:1_18:1	5377.71	2768.46	2287.82	1517.94	11871.07	11146.76	2580.70	946.41
770.55469	PC 35:3	104.51	37.28	114.81	68.32	589.11	522.94	97.15	25.28
770.56512	PC 35:3 I PC 17:1_18:2	380.46	160.60	674.23	660.93	3041.11	4305.34	502.58	181.20
768.55847	PC 35:4	44.31	24.12	35.06	28.67	111.18	219.50	23.64	10.51

**Mass** *m/z*	**Metabolite name**	**CTR**		**SCH**		**NF**		**PNF**	
		mean	*dev*	*mean*	*dev*	*mean*	*dev*	*mean*	*dev*
768.55469	PC 35:4 | PC 15:0_20:4	2062.90	1588.64	1270.88	871.20	4220.43	2750.64	1461.58	467.50
766.54376	PC 35:5 | PC 16:0_19:5	231.84	192.00	57.51	34.87	153.15	100.46	59.54	27.19
766.54053	PC 35:5 | PC 18:1_17:4	120.56	67.23	81.78	44.80	265.96	185.29	80.32	41.95
788.61981	PC 36:1	54.70	41.02	29.54	17.64	35.05	11.46	33.59	29.84
786.60205	PC 36:2	60.31	38.39	37.54	24.45	50.14	23.18	48.70	42.69
782.55377	PC 36:4	1762.55	1260.83	1705.74	1225.27	2649.54	1205.22	1473.15	1062.01
782.56934	PC 36:4 | PC 18:1_18:3	2313.91	926.09	2636.12	2106.71	8622.16	3931.41	2076.25	1379.16
782.57104	PC 36:4 | PC 18:2_18:2	11756.44	3503.44	22383.16	19653.43	60677.58	53893.81	18267.29	7221.14
780.55859	PC 36:5	101.29	54.33	165.84	154.38	179.04	150.75	55.33	21.31
780.54773	PC 36:5 | PC 16:0_20:5	29512.83	24641.05	11325.15	7585.71	40798.37	26495.47	17435.13	16816.32
780.55554	PC 36:5 | PC 16:1_20:4	31304.54	24094.84	27132.40	19901.69	46339.14	23452.98	22461.47	14588.27
780.55621	PC 36:5 | PC 18:2_18:3	170.21	94.97	441.47	574.27	564.00	725.13	110.48	34.45
778.53992	PC 36:6	422.52	213.43	565.56	424.25	1271.56	726.63	760.06	428.78
778.53912	PC 36:6 | PC 14:0_22:6	337.05	255.63	358.04	366.00	471.10	246.47	195.64	99.34
776.52258	PC 36:7	88.91	85.72	117.04	103.82	123.00	97.54	56.85	28.32
798.59998	PC 37:3	398.12	163.78	171.09	103.85	1047.26	1220.34	189.64	37.62
796.58649	PC 37:4 | PC 17:0_20:4	4601.06	2079.68	2193.29	1499.28	11093.34	12588.78	2366.01	410.44
794.57257	PC 37:5 | PC 17:1_20:4	367.98	179.92	255.79	169.48	813.20	583.84	229.72	62.22
792.53619	PC 37:6	73.26	72.95	32.51	17.76	167.60	221.01	32.15	5.46
792.55615	PC 37:6 | PC 15:0_22:6	976.53	946.13	396.72	341.92	1182.14	776.29	486.65	399.19
790.5498	PC 37:7	129.76	105.19	98.46	50.98	214.27	221.23	50.59	9.56
790.54773	PC 37:7 | PC 18:2_19:5	67.26	43.41	69.35	57.08	109.94	64.47	43.91	12.80
810.60461	PC 38:4	18.67	16.68	7.38	7.52	54.38	143.96	6.15	4.76
808.57196	PC 38:5 | PC 16:0_22:5	12235.00	13488.23	3274.07	2899.36	3735.40	3236.84	5825.38	2380.23
808.5769	PC 38:5 | PC 20:1_18:4	252.69	239.60	78.52	33.90	96.56	93.30	111.20	52.04
806.5766	PC 38:6	211.45	89.33	237.77	176.76	433.66	196.83	123.08	46.67
806.55536	PC 38:6 | PC 16:0_22:6	618.62	871.26	107.47	58.10	343.83	594.67	208.20	25.73

Mass *m/z*	Metabolite name	CTR		SCH		NF		PNF	
		mean	*dev*	*mean*	*dev*	*mean*	*dev*	*mean*	*dev*
806.56964	PC 38:6 ǀ PC 18:2_20:4	45329.30	23340.88	40689.70	27542.55	80704.83	38627.67	27950.56	13899.03
804.55768	PC 38:7	504.24	322.08	605.33	608.56	936.08	695.22	246.07	126.31
804.56024	PC 38:7 ǀ PC 16:1_22:6	725.34	472.47	524.76	363.69	937.20	534.46	290.31	138.21
804.5531	PC 38:7 ǀ PC 18:2_20:5	176.70	89.40	444.84	694.42	367.63	212.53	144.12	143.36
822.60577	PC 39:5	480.15	582.72	76.76	60.35	434.44	550.01	46.85	12.87
820.59052	PC 39:6	112.09	129.79	21.50	9.28	74.42	58.37	22.79	4.88
818.56165	PC 39:7	37.99	23.68	34.80	22.26	115.34	87.83	34.24	7.88
814.53583	PC 39:9 ǀ PC 20:4_19:5	38.97	17.55	17.75	10.40	92.11	74.61	15.55	5.82
832.5766	PC 40:7	100.47	121.09	29.98	21.13	91.46	126.44	49.41	12.54
832.58551	PC 40:7 ǀ PC 18:2_22:5	215.96	101.80	199.91	166.06	459.64	283.89	144.35	71.04
832.58356	PC 40:7 ǀ PC 20:3_20:4	1685.33	845.13	1376.02	1211.65	3223.12	1670.58	586.58	188.73
830.55743	PC 40:8	1082.27	1520.77	162.83	88.64	281.87	185.99	174.08	73.98
830.56812	PC 40:8 ǀ PC 18:2_22:6	6281.15	3887.12	6466.18	5568.49	12190.59	7519.45	4150.67	2189.21
830.57166	PC 40:8 ǀ PC 20:4_20:4	15645.71	11595.01	13032.53	8786.01	22441.88	11167.35	7927.21	4027.34
830.55096	PC 40:8 ǀ PC 22:3_18:5	416.25	619.36	44.69	27.23	88.09	89.81	75.89	24.80
828.55389	PC 40:9 ǀ PC 20:4_20:5	80.02	72.85	100.87	150.24	107.86	59.63	32.91	8.61
850.63599	PC 41:5	31.51	9.64	22.39	6.19	103.30	118.07	25.04	12.36
848.62872	PC 41:6 ǀ PC 17:3_24:3	251.48	277.97	30.30	25.07	203.51	299.64	9.51	5.38
846.60706	PC 41:7	109.53	115.66	30.81	24.48	119.93	116.76	18.27	2.38
852.55475	PC 42:11	18.51	15.68	14.26	16.68	22.37	13.62	8.85	4.96
858.61761	PC 42:8	190.45	239.43	37.13	24.96	105.24	56.72	48.00	26.61
858.58362	PC 42:8 ǀ PC 21:4_21:4	251.12	336.54	41.26	29.61	103.49	62.60	66.20	24.28
856.58289	PC 42:9 ǀ PC 20:4_22:5	257.33	130.41	111.71	130.28	288.20	209.61	85.23	42.93
870.6059	PC 43:9	475.41	605.67	69.06	55.46	425.35	636.03	30.86	5.73
882.59766	PC 44:10 ǀ PC 22:4_22:6	78.48	37.23	39.50	28.73	182.96	157.45	42.05	26.26
880.58551	PC 44:11 ǀ PC 22:5_22:6	121.79	52.16	91.91	91.61	169.38	118.54	55.12	34.21
878.57495	PC 44:12 ǀ PC 22:6_22:6	92.42	45.79	69.37	75.42	128.73	109.95	40.53	29.37

**Mass** *m/z*	**Metabolite name**	**CTR**		**SCH**		**NF**		**PNF**	
		mean	*dev*	*mean*	*dev*	*mean*	*dev*	*mean*	*dev*
884.60669	PC 44:9	320.16	513.83	23.75	18.08	420.83	512.56	36.97	35.79
894.60706	PC 45:11	132.61	197.22	21.36	19.66	58.64	72.68	6.75	2.79
924.74841	PC 46:3	8.40	7.23	14.25	18.81	10.38	9.20	14.71	9.91
916.68274	PC 46:7	5.85	2.69	6.72	4.84	22.06	28.34	9.14	9.99
678.54431	PC O-29:0	80.51	90.88	27.95	13.45	148.11	127.65	60.38	55.32
676.52856	PC O-29:1	60.25	37.82	24.90	18.27	74.37	61.90	29.53	18.46
692.56409	PC O-30:0	248.71	226.81	253.07	317.97	412.51	297.97	134.13	71.59
690.54309	PC O-30:1	135.72	162.81	51.59	27.67	220.41	337.41	108.94	70.64
688.52808	PC O-30:2	115.60	109.51	86.54	97.24	150.69	120.85	123.01	100.01
686.51166	PC O-30:3	36.21	23.65	19.05	10.28	77.60	104.73	35.30	31.47
678.46918	PC O-30:7	438.52	281.20	162.03	138.58	233.21	208.41	171.75	75.51
702.54431	PC O-31:2	34.81	33.93	48.12	67.92	65.73	61.99	30.76	23.17
694.46637	PC O-31:6	72.70	56.65	34.72	32.04	38.56	26.54	29.89	10.88
690.43719	PC O-31:8	168.83	223.96	25.84	11.83	53.16	33.86	38.65	10.40
718.56635	PC O-32:1	3439.39	4829.68	845.54	714.59	5921.49	7034.76	2533.34	2947.54
714.54639	PC O-32:3	75.91	43.65	75.28	61.30	239.74	185.45	198.84	247.39
712.53009	PC O-32:4	73.69	74.28	23.67	22.68	273.71	676.22	18.68	7.06
710.51318	PC O-32:5	13.14	21.46	7.64	6.00	88.73	250.54	4.16	0.92
708.48212	PC O-32:6	163.24	137.17	59.42	87.32	375.57	638.60	45.26	39.18
706.46802	PC O-32:7	118.35	136.80	54.84	79.55	269.59	448.33	34.76	31.10
704.45258	PC O-32:8	74.86	47.00	21.39	25.42	65.39	73.22	17.49	15.47
734.60089	PC O-33:0	61.21	11.13	66.90	43.95	288.85	291.81	65.31	19.25
730.56635	PC O-33:2	3133.76	2245.46	1984.99	1700.60	4376.76	1983.43	1848.94	600.64
728.56171	PC O-33:3	94.83	46.69	225.10	290.48	147.11	72.12	61.41	18.03
724.53229	PC O-33:5	66.98	52.81	1023.00	2561.64	145.12	258.45	28.27	10.79

**Mass** *m/z*	**Metabolite name**	**CTR**		**SCH**		**NF**		**PNF**	
		mean	*dev*	*mean*	*dev*	*mean*	*dev*	*mean*	*dev*
722.53271	PC O-33:6	45.89	36.66	24.54	15.98	474.27	1306.45	47.94	17.71
720.48364	PC O-33:7	57.67	34.40	15.34	16.41	56.62	60.82	17.93	13.12
744.5824	PC O-34:2	1083.49	712.57	857.61	596.54	7251.05	9374.66	2142.33	2174.51
742.56506	PC O-34:3	6214.95	3030.59	4645.95	3302.05	68882.95	118272.62	8828.76	5950.93
740.55457	PC O-34:4	178.24	94.47	111.28	64.95	628.24	781.37	153.25	110.49
738.54285	PC O-34:5	110.39	136.68	24.61	26.67	88.76	93.32	8.62	6.54
736.54657	PC O-34:6	161.21	141.06	380.28	1071.23	75.02	103.02	78.06	25.86
730.46783	PC O-34:9	125.46	156.29	20.77	14.18	36.85	37.99	23.23	5.02
754.56049	PC O-35:4	25.16	16.03	56.24	67.84	79.04	117.35	22.48	9.51
752.55579	PC O-35:5	161.57	190.33	52.06	35.46	177.66	182.17	37.33	9.74
750.56281	PC O-35:6	29.19	19.10	51.54	49.14	32.69	20.39	32.44	12.16
748.53906	PC O-35:7	44.86	33.72	23.27	13.70	480.86	1306.16	50.71	22.20
766.57227	PC O-36:5	295.53	251.66	153.61	83.77	627.41	530.12	160.62	79.54
764.56232	PC O-36:6	752.47	904.73	279.21	175.81	1765.27	1844.28	361.11	218.09
760.53278	PC O-36:8	521.97	921.53	48.96	25.11	149.71	127.32	51.84	27.82
758.50134	PC O-36:9	67.10	98.05	8.59	3.83	17.62	14.05	13.46	2.36
788.65344	PC O-37:1	3476.58	913.67	1070.49	622.37	10870.28	15171.29	1041.16	292.17
776.57922	PC O-37:7	69.55	53.83	44.17	55.48	80.15	56.87	40.47	24.91
774.5658	PC O-37:8	193.44	130.88	125.26	95.13	1887.08	5214.15	207.87	98.45
772.55371	PC O-37:9	177.63	168.65	26.11	17.18	95.70	75.51	111.37	187.16
792.59137	PC O-38:6	324.29	296.90	177.56	111.12	651.84	549.49	142.89	32.64
790.5744	PC O-38:7	138.01	175.60	54.13	26.12	124.48	60.79	44.38	13.55
788.5567	PC O-38:8	251.34	275.43	74.60	45.65	192.41	149.18	57.66	17.38
798.56134	PC O-39:10	101.17	62.64	57.93	42.19	165.65	109.53	129.53	69.24
804.59296	PC O-39:7	55.58	39.30	28.28	17.60	170.23	195.87	43.55	21.76
800.573	PC O-39:9	48.95	29.86	34.19	21.70	100.43	104.72	58.03	8.94
826.67401	PC O-40:3	79.16	45.18	86.27	48.80	234.49	210.70	67.06	2.57

Mass *m/z*	Metabolite name	CTR		SCH		NF		PNF	
		mean	*dev*	*mean*	*dev*	*mean*	*dev*	*mean*	*dev*
818.60309	PC O-40:7	126.73	125.73	18.49	22.19	74.12	86.96	56.44	93.12
816.5921	PC O-40:8	90.32	95.69	60.68	59.33	155.51	120.01	36.08	7.78
814.60052	PC O-40:9	89.29	65.61	38.80	58.28	58.56	56.72	45.50	41.41
826.5802	PC O-41:10	172.87	176.24	48.90	52.76	116.04	79.88	41.14	18.68
834.64093	PC O-41:6	837.23	526.26	172.99	113.78	1085.43	1198.09	208.50	119.79
858.73193	PC O-42:1	29.38	12.65	13.50	7.58	34.84	24.97	43.03	53.13
840.59558	PC O-42:10	137.11	193.29	33.75	20.02	93.78	78.05	25.90	7.05
838.57361	PC O-42:11	66.43	84.06	13.80	6.80	35.98	23.99	14.13	3.95
846.6355	PC O-42:7	11.91	3.61	16.96	10.81	68.76	58.97	22.97	6.31
844.61328	PC O-42:8	377.19	467.94	110.05	88.72	276.96	176.91	58.58	25.60
842.59674	PC O-42:9	81.62	105.08	17.06	6.85	42.33	23.47	25.71	9.76
874.66992	PC O-44:7	23.64	11.51	40.93	35.76	108.09	94.25	41.58	14.74
538.35077	PE 21:0	120.89	204.44	9.31	12.76	17.30	10.09	40.51	40.80
536.33514	PE 21:1	2578.37	4820.83	41.44	22.36	162.29	147.00	721.59	803.21
530.28503	PE 21:4	590.72	883.85	51.79	59.23	131.65	111.68	248.79	236.62
558.31787	PE 23:4 | PE 7:0_16:4	4139.06	4393.48	324.16	291.14	762.80	1010.80	458.59	264.74
592.41016	PE 25:1	63.90	97.73	5.76	5.09	19.20	34.97	11.78	9.22
586.34882	PE 25:4	204.00	331.36	15.18	12.28	31.44	25.92	31.54	26.30
664.48615	PE 30:0	18.08	11.89	15.78	12.16	40.21	24.13	21.91	11.93
662.47296	PE 30:1 | PE 14:0_16:1	44.93	42.13	28.45	16.90	144.94	236.69	44.66	25.25
688.49725	PE 32:2	73.60	71.94	72.82	98.72	167.17	294.01	68.70	34.85
688.49445	PE 32:2 | PE 16:1_16:1	126.61	154.99	160.62	283.78	138.72	82.56	449.01	680.87
686.47589	PE 32:3	20.82	12.01	20.21	13.99	32.72	20.50	12.55	7.18
714.51398	PE 34:3 | PE 16:1_18:2	66.91	51.41	60.92	32.46	212.71	167.83	116.55	80.09
712.49066	PE 34:4	195.38	177.08	92.45	74.65	230.75	118.27	155.67	114.89
742.55444	PE 36:3 | PE 18:1_18:2	4700.25	4301.88	2473.54	1757.92	16993.02	15859.27	4274.01	3134.80
740.5274	PE 36:4	121.56	117.75	88.58	60.29	327.64	424.61	150.20	76.34

Mass	Metabolite name	CTR		SCH		NF		PNF	
m/z		*mean*	*dev*	*mean*	*dev*	*mean*	*dev*	*mean*	*dev*
738.51001	PE 36:5 | PE 16:1_20:4	190.50	153.53	126.21	90.66	283.71	159.09	242.37	199.43
736.51904	PE 36:6	57.61	39.05	226.50	361.88	69.56	38.19	28.46	13.85
760.5824	PE 37:1	296.12	189.38	75.12	86.66	127.44	84.56	49.04	21.54
756.54004	PE 37:3	161.60	38.30	35.27	36.98	350.84	440.21	54.17	57.22
766.5376	PE 38:5	154.46	105.20	48.57	34.54	149.97	42.40	51.04	25.38
764.53302	PE 38:6 | PE 18:2_20:4	479.83	252.88	304.61	211.84	804.81	427.61	240.84	111.97
762.50769	PE 38:7 | PE 16:1_22:6	244.43	116.39	303.25	228.30	622.42	497.92	236.98	186.34
778.54822	PE 39:6	68.07	32.55	29.27	15.76	185.84	214.74	27.22	10.42
796.58594	PE 40:4 | PE 18:0_22:4	2056.45	1254.16	749.14	553.88	5298.69	7429.46	733.50	440.75
788.51794	PE 40:8 | PE 18:2_22:6	225.43	129.93	158.47	131.90	385.12	266.80	114.55	52.81
812.52948	PE 42:10 | PE 20:4_22:6	250.89	159.44	64.93	38.75	201.56	121.22	78.34	61.32
852.54858	PE 45:11	128.29	84.54	119.92	91.47	270.89	181.32	92.53	61.73
572.3681	PE O-25:4	298.16	306.77	61.47	42.48	86.02	55.77	64.17	49.77
668.46887	PE O-32:5	13.61	10.88	11.03	9.10	131.02	371.17	7.87	3.04
724.52234	PE O-36:5	48.66	18.58	41.97	28.19	436.79	534.77	71.08	34.54
746.51782	PE O-38:8	38.16	25.91	280.61	683.08	37.13	28.40	16.08	7.86
772.53009	PE O-40:9	347.28	254.85	132.66	201.43	281.60	228.85	148.75	111.15
782.50043	PE O-41:11	100.91	78.03	52.77	26.71	108.77	62.76	52.68	29.78
620.46198	PE P-28:0 | PE P-16:0_12:0	36.12	48.21	10.90	5.59	61.39	84.42	43.26	52.02
674.51123	PE P-32:1 | PE P-16:0_16:1	447.05	716.36	95.31	113.66	912.16	877.92	964.73	1262.15
682.48859	PE P-33:4 | PE P-13:0_20:4	56.66	43.07	46.04	25.18	156.04	195.23	46.95	22.02
696.50067	PE P-34:4 | PE P-14:0_20:4	76.69	48.24	31.84	20.75	125.51	116.08	30.73	12.97
730.56763	PE P-36:1 | PE P-18:0_18:1	18.62	32.70	1.03	0.59	2.94	4.03	15.21	25.99
722.52112	PE P-36:5 | PE P-16:0_20:5	50.35	49.88	62.81	139.68	63.36	121.46	19.65	5.67
722.50879	PE P-36:5 | PE P-16:1_20:4	81.93	75.17	65.74	47.54	215.17	178.31	70.30	40.88
748.53204	PE P-38:6 | PE P-18:2_20:4	276.33	244.56	211.55	110.01	454.36	247.39	196.63	81.87
742.49261	PE P-38:9 | PE P-16:3_22:6	46.12	26.60	41.62	36.20	208.38	311.45	22.38	9.77

**Mass** *m/z*	**Metabolite name**	**CTR**		**SCH**		**NF**		**PNF**	
		mean	*dev*	*mean*	*dev*	*mean*	*dev*	*mean*	*dev*
766.57428	PE P-39:4 I PE P-17:0_22:4	195.74	137.34	163.88	126.19	771.30	922.16	115.70	77.26
776.55682	PE P-40:6 I PE P-18:0_22:6	80.51	59.61	39.85	33.41	79.63	57.63	25.41	4.56
830.61584	PE P-44:7 I PE P-22:1_22:6	14.05	3.56	29.73	22.89	102.63	100.17	23.69	11.40
788.54163	PG 36:4 I PG 18:2_18:2	45.77	44.68	28.66	25.07	53.61	42.94	19.60	6.71
814.56177	PG 38:5 I PG 18:1_20:4	171.12	204.04	42.99	21.07	87.66	41.46	38.49	15.26
595.38434	SM 27:6;2O	186.06	219.99	31.29	26.95	64.98	45.65	43.02	27.06
619.48303	SM 28:1;2O	47.91	44.62	52.16	54.72	47.88	24.50	40.34	21.46
647.48468	SM 29:2;3O	146.26	106.03	186.62	297.38	154.82	97.90	81.18	36.05
649.52686	SM 30:0;2O	37.30	34.50	31.38	39.56	59.76	44.51	90.54	92.31
647.51318	SM 30:1;2O I SM 16:1;2O/14:0	6964.25	6554.18	10847.10	26420.01	3808.68	2281.18	4384.14	3810.27
645.50098	SM 30:2;2O I SM 15:0;2O/15:2	40.17	37.04	27.79	14.54	51.05	21.42	48.31	33.28
661.49225	SM 30:2;3O	103.63	112.87	15.89	9.61	42.87	31.84	31.69	9.47
661.52844	SM 31:1;2O I SM 18:1;2O/13:0	1429.87	1454.26	1059.25	1223.02	1732.22	1830.24	1322.05	1098.09
677.52136	SM 31:1;3O	10.01	8.56	6.36	6.06	149.16	423.38	8.53	3.38
677.56329	SM 32:0;2O	3638.40	3063.05	3746.37	4671.72	5524.75	4253.32	8770.90	9300.49
675.54639	SM 32:1;2O	130.72	119.93	107.82	132.13	185.66	121.20	106.59	52.56
675.54419	SM 32:1;2O I SM 16:1;2O/16:0	160449.52	168108.32	87707.81	88752.82	143301.42	88339.93	84722.92	55135.07
691.53912	SM 32:1;3O	195.26	252.03	75.20	97.72	1377.95	3254.85	119.58	77.08
673.53015	SM 32:2;2O I SM 18:2;2O/14:0	5309.25	5093.67	3080.83	2781.59	3517.74	1707.73	4583.73	3672.42
689.52795	SM 32:2;3O	55.48	44.72	48.98	90.36	28.00	16.21	19.92	9.54
667.47504	SM 32:5;2O	103.41	62.53	49.29	69.34	85.54	55.56	34.22	31.69
679.44794	SM 32:7;3O	59.47	47.69	160.10	383.72	34.38	21.26	24.05	2.72
691.57806	SM 33:0;2O	2049.32	2010.19	2221.98	3330.46	2239.55	1568.06	965.98	581.91
707.57294	SM 33:0;3O	6076.15	3130.14	2245.87	1296.99	6745.20	5402.77	3183.85	1590.45
689.5603	SM 33:1;2O I SM 17:1;2O/16:0	42664.17	41073.71	52119.65	71675.76	52115.02	39073.30	24603.67	15404.48

Mass *m/z*	Metabolite name	CTR		SCH		NF		PNF	
		mean	*dev*	*mean*	*dev*	*mean*	*dev*	*mean*	*dev*
689.56494	SM 33:1;2O|SM 18:1;2O/15:0	444.84	593.69	198.08	216.94	385.37	279.83	135.93	47.73
689.55975	SM 33:1;2O|SM 21:1;2O/12:0	402.84	529.74	204.57	227.79	337.10	207.32	129.67	48.28
705.5553	SM 33:1;3O	20.09	17.99	13.78	14.41	194.05	520.53	27.15	20.69
687.54419	SM 33:2;2O|SM 17:2;2O/16:0	368.79	356.35	298.47	371.01	318.10	178.85	187.02	139.62
703.53656	SM 33:2;3O	757.33	676.61	276.79	491.70	191.13	174.18	254.42	220.93
721.58521	SM 34:0;3O	40.49	40.20	28.32	36.99	195.97	347.67	189.23	228.99
703.57611	SM 34:1;2O	10815.19	13553.15	4663.83	5472.89	5899.19	2871.25	3320.63	1853.57
703.57336	SM 34:1;2O|SM 18:1;2O/16:0	1496.98	981.94	2378.29	3545.14	2483.01	1692.63	2079.86	1854.83
719.57214	SM 34:1;3O	53472.16	68091.30	13584.47	13718.21	87717.66	90745.66	25615.87	9404.19
701.55981	SM 34:2;2O|SM 18:1;2O/16:1	746.10	665.22	1139.36	2063.05	1233.79	1142.76	289.22	96.22
701.56012	SM 34:2;2O|SM 18:2;2O/16:0	188625.85	234383.05	94385.03	103433.49	173115.56	142220.35	69231.60	41424.79
717.55682	SM 34:2;3O	365.53	517.87	125.39	122.10	472.33	625.18	314.53	257.65
719.60522	SM 35:0;2O	1085.72	651.56	1131.73	1916.05	5092.51	7617.22	981.40	827.94
733.58398	SM 35:1;3O	24.90	30.70	18.14	30.22	449.70	1341.40	14.26	7.21
715.57959	SM 35:2;2O|SM 17:1;2O/18:1	11562.52	11838.60	9639.60	15372.40	6799.29	4025.35	4070.05	2577.84
731.56702	SM 35:2;3O	1803.85	1317.38	1436.38	1222.53	3436.94	1699.81	1757.97	763.06
711.54138	SM 35:4;2O	572.40	491.08	1696.71	2396.94	1790.77	2148.90	448.48	340.04
723.50562	SM 35:6;3O	56.09	46.54	57.29	52.64	55.48	39.94	31.84	16.94
747.60461	SM 36:1;3O	1131.20	1274.47	849.86	1422.01	5363.04	8886.49	744.80	401.37
745.58667	SM 36:2;3O	343.35	210.81	243.63	161.94	1610.51	1706.17	544.49	544.60
727.5777	SM 36:3;2O	98.79	62.74	313.70	528.34	130.31	78.81	92.82	45.28
727.5752	SM 36:3;2O|SM 18:2;2O/18:1	493.66	273.99	1723.95	2835.62	698.65	419.41	280.54	125.20
743.5672	SM 36:3;3O	94.57	97.69	70.31	92.55	147.03	146.14	45.01	13.10
737.51453	SM 36:6;3O	15.70	8.17	20.21	10.31	48.00	29.01	19.66	6.81
719.5365	SM 36:7;2O	201.58	197.07	437.24	1175.98	57.60	50.33	60.92	34.49
755.60516	SM 38:3;2O|SM 18:2;2O/20:1	100.29	50.53	118.17	108.58	383.83	321.92	115.08	60.72
751.5592	SM 38:5;2O	253.45	332.68	67.10	115.88	30.58	66.93	151.75	192.93
749.5592	SM 38:6;2O	55.50	38.01	91.46	135.10	96.34	96.84	38.49	12.52

Mass m/z	Metabolite name	CTR		SCH		NF		PNF	
		mean	dev	mean	dev	mean	dev	mean	dev
765.54901	SM 38:6;3O	239.83	213.09	116.04	69.58	465.50	373.09	109.90	54.89
789.64514	SM 39:1;3O	16.54	7.10	8.53	3.30	27.36	15.39	12.76	3.29
757.54529	SM 39:9;2O	64.47	60.64	41.11	27.94	65.54	38.74	34.66	15.86
801.65289	SM 40:2;3O	34.78	48.70	11.15	13.02	15.55	8.57	14.55	10.52
777.57251	SM 40:6;2O I SM 18:2;2O/22:4	125.29	84.43	145.88	203.62	67.08	60.24	74.94	31.21
793.59698	SM 40:6;3O	140.82	108.86	86.27	53.04	342.32	285.22	76.72	20.54
791.58466	SM 40:7;3O	328.53	492.07	92.44	53.73	365.50	348.80	88.33	27.23
813.68695	SM 42:2;2O	73.21	49.99	82.11	125.88	43.52	16.44	55.12	57.50
813.67969	SM 42:2;2O I SM 18:1;2O/24:1	423251.77	143565.90	97005.29	101907.71	275881.67	327906.39	69820.41	34874.17
829.67944	SM 42:2;3O	104.82	74.35	50.45	60.68	91.76	49.17	60.93	20.11
827.66516	SM 42:3;3O	52.12	39.45	25.50	38.96	28.72	12.36	24.50	13.49
799.5741	SM 42:9;2O	73.64	62.11	31.10	30.29	57.98	34.19	54.21	50.78
827.60028	SM 44:9;2O	86.13	67.96	25.90	29.93	61.53	26.24	42.81	28.59
859.6474	SM 45:8;3O	21.72	23.14	13.97	12.49	18.47	13.18	18.12	5.22
859.63837	SM 45:8;3O I SM 31:6;2O(FA 14:1)	52.34	32.46	24.69	13.72	73.09	59.53	22.20	9.89
861.67188	SM 46:6;2O I SM 18:1;2O/28:5	149.14	92.11	88.69	95.63	107.11	112.26	135.08	45.91
385.3472	ST 27:2;O	4467.27	6642.39	1666.91	2478.60	2941.50	3868.77	1940.33	2511.61
423.34756	ST 28:1;O	1303.54	1804.06	67.66	62.89	1987.63	5516.27	1052.49	2077.93
421.35089	ST 28:2;O	36.52	24.00	38.41	44.09	240.64	220.40	23.40	8.65
419.31726	ST 28:3;O	1484.73	1498.73	1486.62	2944.32	2140.10	2006.81	523.52	91.94
377.32697	ST 28:4;O	76.44	45.51	108.12	154.12	803.96	792.94	57.10	25.17
437.38055	ST 29:1;O	28.36	21.11	27.73	37.95	99.26	111.41	14.01	4.27
435.34729	ST 29:2;O	3481.98	2537.06	141.28	134.31	3752.13	10401.52	1296.57	2529.56
488.3967	TG 24:0 I TG 8:0_8:0_8:0	11795.93	10102.69	668.33	983.73	4527.50	6043.32	1271.40	2241.32
516.42743	TG 26:0 I TG 8:0_8:0_10:0	20062.79	14467.99	1631.29	2100.05	8873.93	9128.93	2250.81	3732.90
535.39825	TG 27:0 I TG 9:0_9:0_9:0	40.21	48.08	48.35	85.14	32.78	25.54	136.65	159.70
626.53607	TG 34:1 I TG 8:0_8:0_18:1	195.06	186.15	25.82	11.89	280.69	470.38	8332.98	16629.32

Mass *m/z*	Metabolite name	CTR		SCH		NF		PNF	
		mean	*dev*	*mean*	*dev*	*mean*	*dev*	*mean*	*dev*
661.53308	TG 36:0ǀTG 12:0_12:0_12:0	169.89	189.94	73.16	66.22	213.70	145.51	62.65	10.62
652.55145	TG 36:2ǀTG 8:0_10:0_18:2	54.08	40.38	8.58	3.73	77.97	118.44	4392.42	8775.57
726.57635	TG 39:2;2OǀTG 8:0_16:1_15:1;2O	8.73	4.56	3.98	4.59	7.38	6.54	60.40	116.63
740.67133	TG 42:0ǀTG 12:0_14:0_16:0	1592.11	1553.59	108.48	79.04	3816.13	8555.81	3088.92	6083.90
752.63708	TG 42:2;1OǀTG 16:1_18:1_8:0;1O	39.38	32.09	3.80	2.36	17.10	20.01	279.62	549.91
750.62512	TG 42:3;1OǀTG 9:0_16:0_17:3;1O	88.46	37.87	50.51	37.12	150.93	135.14	148.55	200.46
755.62231	TG 43:2ǀTG 11:0_16:1_16:1	23.85	16.35	177.09	409.51	24.56	48.97	11.16	8.13
750.65656	TG 43:2ǀTG 14:0_14:1_15:1	462.90	471.25	7.07	3.16	510.74	965.32	1833.37	3657.65
760.64856	TG 44:4ǀTG 8:0_16:0_20:4	196.56	188.29	6.74	3.85	84.75	134.04	1304.88	2602.11
760.64337	TG 44:4ǀTG 8:0_18:2_18:2	808.36	760.36	10.19	8.78	151.12	283.50	2983.52	5954.91
778.6886	TG 45:2ǀTG 16:0_13:1_16:1	837.63	776.48	19.85	8.07	1038.06	2201.44	3220.81	6414.83
776.66754	TG 45:3ǀTG 11:0_16:1_18:2	139.67	123.56	2.71	2.05	90.98	144.42	818.45	1633.68
813.68085	TG 47:1ǀTG 13:0_15:0_19:1	98.97	99.80	17.79	18.41	42.88	103.03	100.15	186.75
806.72394	TG 47:2ǀTG 15:0_14:1_18:1	2097.70	2259.58	107.50	46.60	3294.58	5757.33	7932.22	15743.47
804.70288	TG 47:3ǀTG 14:1_16:1_17:1	603.86	515.00	7.45	3.09	397.23	722.77	2979.38	5949.77
838.73944	TG 48:1;1OǀTG 16:0_16:1_16:0;1O	68.82	77.20	1.60	1.62	47.80	97.01	1296.79	2592.16
822.75879	TG 48:1ǀTG 14:0_16:0_18:1	25123.99	15737.36	5440.23	4821.43	32380.35	58433.51	18047.24	32992.02
816.71704	TG 48:4ǀTG 14:1_16:1_18:2	1956.12	1512.03	20.57	25.44	480.60	922.90	3049.34	6086.18
814.68445	TG 48:5ǀTG 12:0_18:2_18:3	483.34	542.59	9.34	6.50	515.87	1283.73	2183.68	4355.85
812.68842	TG 48:6ǀTG 14:1_14:1_20:4	129.48	106.84	6.26	8.67	39.69	81.34	398.28	792.64
834.74188	TG 49:2ǀTG 14:0_16:1_19:1	157.36	153.11	3.98	5.56	34.23	62.20	270.48	528.81
832.73578	TG 49:3ǀTG 15:0_16:1_18:2	2001.33	1512.39	32.20	17.45	2290.71	4877.64	6963.03	13895.99
830.71576	TG 49:4ǀTG 14:1_17:1_18:2	228.88	195.81	5.16	4.97	146.55	344.62	958.59	1912.06
857.75043	TG 50:0ǀTG 16:0_16:0_18:0	458.65	398.74	2.60	2.86	135.79	275.31	4301.13	8596.07
866.77942	TG 50:1;1OǀTG 16:0_16:1_18:0;1O	412.50	505.13	1.81	1.97	130.50	267.08	4484.67	8966.60
864.76349	TG 50:2;1OǀTG 16:0_16:1_18:1;1O	576.65	696.12	2.09	1.41	244.66	511.83	9623.51	19244.54

**Mass** *m/z*	**Metabolite name**	**CTR**		**SCH**		**NF**		**PNF**	
		mean	*dev*	*mean*	*dev*	*mean*	*dev*	*mean*	*dev*
862.75073	TG 50:3;1O丨TG 16:1_18:1_16:1;1O	267.58	253.63	3.31	3.49	143.76	294.54	4574.51	9144.01
844.74396	TG 50:4丨TG 16:1_16:1_18:2	5024.62	3905.01	131.56	191.26	2413.01	4848.23	7732.11	15441.06
842.71893	TG 50:5丨TG 14:1_18:2_18:2	3249.95	2623.52	22.59	20.53	1391.03	2908.61	9126.67	18241.97
869.7431	TG 51:1丨TG 16:0_16:0_19:1	65.79	52.13	1.91	1.51	39.75	72.39	231.53	460.82
864.79407	TG 51:1丨TG 16:0_17:0_18:1	6048.39	4630.96	239.87	262.86	13591.05	39210.81	8189.90	16265.78
862.78003	TG 51:2丨TG 16:0_17:1_18:1	15118.41	11702.68	707.79	635.95	22494.00	60003.28	13168.05	26067.14
858.75281	TG 51:4丨TG 16:1_17:1_18:2	1286.18	1214.15	21.60	15.80	1326.03	3387.62	2622.30	5229.88
894.8255	TG 52:1;1O丨TG 16:0_18:1_18:0;1O	346.35	334.33	1.27	1.05	72.13	151.96	1750.82	3499.81
883.77643	TG 52:1丨TG 16:0_18:0_18:1	815.85	858.91	3.81	2.96	58.80	108.15	924.05	1842.88
892.7854	TG 52:2;1O丨TG 16:0_18:1_18:1;1O	600.78	678.36	3.40	2.66	220.65	510.20	10539.20	21076.38
888.7572	TG 52:4;1O丨TG 16:0_18:2_18:2;1O	273.92	243.32	2.55	2.00	205.73	547.75	3260.78	6518.85
870.75421	TG 52:5丨TG 16:1_18:2_18:2	18550.22	13528.59	543.43	481.45	15871.22	40081.56	25399.28	50588.13
868.73438	TG 52:6丨TG 16:1_16:1_20:4	2414.39	1873.26	38.34	33.83	1328.60	2982.11	5746.82	11478.07
873.69867	TG 52:6丨TG 18:0_16:1_18:5	70.75	46.19	3.60	2.63	50.43	105.79	26.23	50.75
866.72363	TG 52:7丨TG 16:0_14:1_22:6	1187.75	864.79	8.62	8.56	216.34	524.08	2245.55	4487.22
890.80963	TG 53:2丨TG 16:0_18:1_19:1	16104.35	16190.09	238.03	243.17	27068.34	80235.09	13816.10	27537.12
890.82971	TG 53:2丨TG 17:0_18:1_18:1	19345.77	17364.54	305.56	349.33	18715.02	53983.08	6235.82	12367.86
888.7948	TG 53:3丨TG 17:1_18:1_18:1	11301.77	10841.31	258.69	263.65	14427.79	40568.97	8976.74	17853.97
918.77185	TG 53:4;2O丨TG 18:1_18:1_17:2;2O	5219.50	7319.07	16.97	21.67	147.43	367.07	712.12	1418.47
882.75775	TG 53:6丨TG 15:0_18:2_20:4	122.29	85.02	6.81	6.89	57.81	134.81	375.47	748.34
906.84167	TG 54:1丨TG 16:0_20:0_18:1	3343.51	3175.34	236.30	225.41	16641.25	51204.41	25702.82	51283.75
920.83008	TG 54:2;1O丨TG 16:0_18:1_20:1;1O	95.17	97.80	0.95	1.03	33.28	79.78	612.80	1224.11
918.82343	TG 54:3;1O丨TG 18:1_18:1_18:1;1O	79.17	66.95	1.64	1.14	53.58	135.47	640.94	1279.80
898.78577	TG 54:5丨TG 18:1_18:2_18:2	15972.43	10422.95	3974.27	7690.30	8475.04	18187.12	8406.02	16557.85
896.77228	TG 54:6丨TG 18:2_18:2_18:2	10441.12	7074.91	779.94	724.81	12291.02	32820.40	17165.49	34161.40
894.75238	TG 54:7丨TG 18:2_18:2_18:3	6629.82	4487.28	122.61	145.74	1799.88	4200.89	9767.05	19507.36
948.81818	TG 55:3;O2丨TG 14:1_14:1_15:0;O(FA 12:0)	59.40	66.46	4.10	4.27	8.76	17.18	10.43	18.12

Mass m/z	Metabolite name	CTR		SCH		NF		PNF	
		mean	dev	mean	dev	mean	dev	mean	dev
916.8241	TG 55:3 | TG 18:1_18:1_19:1	3791.06	3395.53	34.82	45.06	4663.23	13601.11	4361.98	8708.49
914.81293	TG 55:4 | TG 18:1_19:1_18:2	1069.57	1038.52	34.58	60.19	1079.94	3149.45	801.02	1594.62
912.80182	TG 55:5 | TG 18:1_18:2_19:2	126.37	127.70	16.28	25.31	84.15	208.45	86.79	167.88
932.85559	TG 56:2 | TG 20:0_18:1_18:1	2147.73	2081.32	180.76	236.20	12140.13	37529.40	22418.68	44771.48
928.83429	TG 56:4 | TG 18:1_18:1_20:2	8215.41	8537.96	1252.08	2380.58	3659.86	10137.91	685.88	1231.03
926.81354	TG 56:5 | TG 16:0_18:1_22:4	19633.06	13855.80	2225.34	4557.87	10657.43	27845.67	12760.49	25426.30
926.82214	TG 56:5 | TG 18:1_18:1_20:3	13844.06	9918.14	1513.94	3115.38	9965.45	26538.29	9261.72	18427.24
924.80444	TG 56:6 | TG 18:1_18:1_20:4	16301.75	12615.72	2688.56	5334.67	9497.76	23451.09	8564.79	16937.85
920.76868	TG 56:8 | TG 16:0_18:2_22:6	8836.12	8065.60	338.35	618.58	3349.70	9298.93	5879.47	11719.38
942.83746	TG 57:4 | TG 18:1_19:1_20:2	63.29	43.84	8.05	15.18	53.64	152.57	102.43	202.89
944.77417	TG 58:10 | TG 18:2_18:2_22:6	243.66	252.63	24.59	36.56	75.52	197.11	41.00	72.98
942.76617	TG 58:11 | TG 18:2_18:3_22:6	36.62	29.50	4.35	3.71	6.45	11.07	40.83	77.07
958.8725	TG 58:3 | TG 22:0_18:1_18:2	456.25	385.83	77.64	134.70	1510.21	4597.48	5142.97	10267.39
954.8493	TG 58:5 | TG 18:1_18:1_22:3	1747.55	1233.50	382.62	783.04	1136.75	3177.95	1336.81	2650.11
952.8277	TG 58:6 | TG 18:1_18:1_22:4	4228.60	3222.95	605.49	1309.57	2541.58	7126.49	2381.66	4737.29
946.79028	TG 58:9 | TG 18:1_18:2_22:6	2154.53	2315.95	184.92	378.40	1133.02	3250.58	1159.81	2301.59
951.75085	TG 58:9 | TG 18:1_20:4_20:4	59.89	55.66	5.46	9.59	23.67	59.63	9.51	17.98
970.87897	TG 59:4 | TG 18:1_23:1_18:2	2.71	1.69	2.32	3.01	8.27	24.24	40.00	78.91
972.80347	TG 60:10 | TG 18:1_20:3_22:6	97.09	75.88	47.01	109.04	23.94	47.50	19.87	33.35
972.80652	TG 60:10 | TG 18:1_20:4_22:5	75.60	68.11	23.78	52.07	26.55	59.69	46.78	89.46
982.87463	TG 60:5 | TG 18:1_18:2_24:2	26.03	19.45	43.05	83.13	38.31	71.65	164.37	324.00
980.86499	TG 60:6 | TG 18:1_20:1_22:4	158.28	106.34	84.74	173.15	105.27	230.44	164.61	319.43
976.83142	TG 60:8 | TG 18:1_20:3_22:4	30.17	20.97	73.57	171.10	13.23	20.76	4.97	3.30
1000.83606	TG 62:10 | TG 18:0_22:4_22:6	26.82	8.10	42.13	99.98	7.28	9.34	5.99	8.10
1126.99402	TG 68:5;O2 | TG 16:1_18:1_18:2;O(FA 16:0)	0.41	0.22	0.06	0.05	1.13	3.41	37.27	74.54
479.37262	TG O-24:0 | TG O-8:0_8:0_8:0	837.49	649.16	120.43	239.81	492.68	904.26	150.63	261.33

Mass m/z	Metabolite name	CTR		SCH		NF		PNF	
		mean	*dev*	*mean*	*dev*	*mean*	*dev*	*mean*	*dev*
488.42084	TG O-25:0 I TG O-9:0_8:0_8:0	742.99	374.85	67.65	69.27	388.25	235.46	98.73	141.21
521.41876	TG O-27:0 I TG O-10:0_8:0_9:0	32.03	10.37	33.39	28.04	185.88	151.35	109.54	98.45
521.42017	TG O-27:0 I TG O-9:0_9:0_9:0	32.15	14.82	39.97	35.57	194.80	158.07	150.78	145.57
577.48102	TG O-31:0 I TG O-9:0_11:0_11:0	17.10	8.45	21.39	18.36	104.75	88.13	49.14	16.51
619.52576	TG O-34:0 I TG O-16:0_9:0_9:0	234.72	191.38	108.28	99.80	378.39	467.20	171.06	151.39
709.58667	TG O-41:4 I TG O-19:4_11:0_11:0	45.76	62.09	101.94	187.84	47.66	41.47	28.02	19.35
724.66956	TG O-42:1 I TG O-16:1_12:0_14:0	527.76	510.65	31.06	76.71	185.17	336.82	349.19	685.49
723.61383	TG O-42:4 I TG O-20:4_11:0_11:0	149.83	297.98	90.86	215.49	1.69	4.79	0.67	0.78
736.66693	TG O-43:2 I TG O-17:1_8:0_18:1	4249.58	3745.41	22.39	18.27	1504.51	3342.91	5278.41	10540.39
794.75507	TG O-47:1 I TG O-15:0_16:0_16:1	116.12	85.86	6.58	5.00	117.29	237.52	92.60	179.59
829.74561	TG O-49:0 I TG O-17:0_16:0_16:0	517.66	680.64	2.13	2.29	37.23	69.62	598.29	1190.90
816.74634	TG O-49:4 I TG O-15:1_16:1_18:2	373.05	278.90	8.95	7.12	169.53	369.51	212.71	416.91
839.74768	TG O-50:2 I TG O-20:2_15:0_15:0	44.37	47.35	1.05	1.30	31.63	60.92	769.02	1537.29
858.79102	TG O-52:4 I TG O-16:0_16:0_20:4	93.67	76.08	30.74	58.54	169.41	488.78	38.40	71.06
884.81085	TG O-54:5 I TG O-16:0_18:1_20:4	57.42	39.01	68.54	146.37	85.19	232.13	17.55	29.06
889.77289	TG O-54:5 I TG O-16:0_19:0_19:5	172.89	148.16	1.88	1.95	133.16	359.43	1447.64	2893.06
922.82062	TG O-57:7 I TG O-21:5_18:1_18:1	928.17	553.06	134.22	206.47	600.79	1373.08	290.40	540.22
970.80145	TG O-61:11 I TG O-19:1_20:4_22:6	292.62	231.20	79.79	193.12	31.12	60.02	75.77	144.44
469.38934	VAE 12:0	59.11	33.62	66.91	69.66	356.75	325.74	233.78	236.01
599.50397	VAE 22:5	53.79	25.75	73.04	87.01	209.01	234.66	62.54	41.01
357.28015	VAE 4:0	24.15	28.20	80.13	161.32	32.25	38.69	3.69	0.74

Table S2. List of all the single lipid species identified using an untargeted lipidomics approach across each histotype. Each lipid was identified with monoisotopic molecular adducts in negative electrospray ionization (*m*/*z*), name, and mass intensities scaled for protein content in each group.

**Average** *m/z*	**Metabolite name**	**CTR**		**SCH**		**NF**		**PNF**	
		mean	***dev***	***mean***	***dev***	***mean***	***dev***	***mean***	***dev***
482.2933	BA 24:1;O2;T	148.85	233.85	58.14	110.68	41.56	60.79	42.84	14.24
498.28717	BA 24:1;O3;T	21.60	12.87	38.01	39.21	84.19	83.46	99.75	55.31
528.27295	BA 24:1;O4;G;S	24539.58	28569.99	2961.82	2850.82	4303.17	5486.04	3927.19	1999.87
514.29041	BA 24:1;O4;T	3.76	3.73	281.49	887.06	3.13	4.72	1.22	0.64
619.42316	CL 56:0|CL 14:0_14:0_14:0_14:0	130.41	208.72	61.18	111.06	28.88	23.74	28.70	19.48
618.41516	CL 56:1|CL 12:0_12:0_16:0_16:1	18.46	34.24	2.91	4.40	1.85	2.27	2.56	2.12
623.40497	CL 57:3|CL 12:0_15:1_15:1_15:1	250.13	255.88	3960.81	11053.51	112.53	184.29	108.91	54.90
628.39093	CL 58:5|CL 14:1_14:1_14:1_16:2	206.57	171.99	113.85	89.71	111.44	124.89	85.60	26.18
647.46063	CL 60:0|CL 12:0_16:0_16:0_16:0	384.94	302.75	286.31	283.15	191.11	156.86	107.85	45.57
644.42407	CL 60:3|CL 12:0_12:0_18:1_18:2	19.09	26.96	11.08	19.28	5.51	5.67	4.23	1.78
643.42462	CL 60:4|CL 14:1_14:1_14:1_18:1	97.83	77.67	64.53	48.59	66.61	88.33	29.02	16.88
657.41956	CL 62:4|CL 14:1_16:1_16:1_16:1	165.90	156.21	56.27	40.82	61.52	112.12	84.10	32.59
667.45496	CL 63:1|CL 16:0_16:0_16:0_15:1	479.23	508.97	361.18	404.10	273.48	275.38	217.51	68.92
675.48639	CL 64:0|CL 16:0_16:0_16:0_16:0	163.57	253.23	70.70	92.34	104.98	217.17	49.72	44.81
673.47058	CL 64:2|CL 12:0_16:0_18:1_18:1	46.17	52.24	15.44	23.19	55.99	135.33	8.25	6.48
673.47229	CL 64:2|CL 16:0_16:0_16:0_16:2	83.55	101.08	16.87	24.27	88.57	221.39	13.56	10.78
669.42389	CL 64:6|CL 16:0_14:1_14:1_20:4	323.26	197.62	157.85	101.11	245.79	130.27	160.73	48.07
680.48224	CL 65:2|CL 16:0_16:0_16:0_17:2	85.88	106.00	37.56	24.17	91.82	196.71	41.05	27.55
677.44824	CL 65:5|CL 16:1_16:1_16:1_17:2	439.97	728.89	1416.49	3319.23	30.34	35.47	30.71	43.40
689.50269	CL 66:0|CL 16:0_16:0_17:0_17:0	184.08	322.46	46.76	24.93	60.25	75.12	45.01	15.68
695.49915	CL 67:1|CL 16:0_16:0_17:0_18:1	16.37	8.79	60.04	100.78	43.47	99.92	13.94	9.34
700.48907	CL 68:3|CL 14:0_16:1_18:1_20:1	36.63	34.59	88.93	184.19	123.66	131.17	110.91	108.70
699.46246	CL 68:4|CL 14:1_18:1_18:1_18:1	170.98	142.98	136.57	179.83	66.31	44.03	35.86	14.66
694.43042	CL 68:9|CL 16:1_16:2_16:2_20:4	492.44	497.34	133.93	75.35	145.97	188.45	229.25	73.84

Average m/z	Metabolite name	CTR		SCH		NF		PNF	
		mean	dev	mean	dev	mean	dev	mean	dev
707.50098	CL 69:3 I CL 24:0_15:1_15:1_15:1	283.00	252.56	828.97	1496.21	160.22	403.78	323.41	284.94
705.48328	CL 69:5 I CL 15:0_16:0_16:0_22:5	9.58	7.58	32.47	68.37	5.21	11.21	6.70	2.89
1434.03735	CL 70:1 I CL 37:0_33:1	64.48	37.59	59.15	60.10	64.53	37.82	74.81	49.62
714.49774	CL 70:3 I CL 16:0_16:0_16:0_22:3	46.07	30.33	114.43	204.39	50.23	85.91	34.45	22.41
714.49335	CL 70:3 I CL 16:0_18:1_18:1_18:1	7.16	5.52	17.00	19.63	9.03	15.79	3.75	1.16
713.48871	CL 70:4 I CL 16:1_18:1_18:1_18:1	6.71	3.91	54.23	61.68	13.16	17.14	13.90	6.66
712.47986	CL 70:5 I CL 16:1_18:1_18:1_18:2	13.20	9.90	102.20	143.13	26.86	32.19	22.15	8.30
711.46515	CL 70:6 I CL 16:0_16:0_16:0_22:6	66.90	40.15	511.41	1300.54	104.03	247.38	31.76	12.63
711.47211	CL 70:6 I CL 16:1_18:1_18:2_18:2	29.05	32.42	80.93	86.98	40.83	35.15	37.57	7.51
1423.95532	CL 70:6 I CL 34:3_36:3	55.62	52.85	122.82	154.58	100.11	147.54	45.15	25.56
721.51434	CL 71:3 I CL 17:0_18:1_18:1_18:1	33.88	26.43	102.30	223.00	22.10	23.67	28.26	22.52
721.50635	CL 71:3 I CL 26:0_15:1_15:1_15:1	13.92	13.12	35.85	59.36	9.08	12.65	13.94	5.25
719.49811	CL 71:5 I CL 16:0_17:0_18:1_20:4	47.92	33.61	197.12	394.46	17.79	20.10	44.81	30.15
717.46948	CL 71:7 I CL 15:0_18:0_16:1_22:6	22.20	32.76	22.18	41.54	3.55	4.11	4.82	2.99
717.49408	CL 71:7 I CL 18:1_17:2_18:2_18:2	56.47	65.44	49.58	68.96	32.76	32.83	52.57	25.07
730.52692	CL 72:1 I CL 16:0_16:0_20:0_20:1	716.14	435.88	662.86	592.71	2839.97	3488.85	537.10	321.34
730.53809	CL 72:1 I CL 18:0_18:0_18:0_18:1	168.16	139.55	102.08	43.30	246.85	200.05	179.51	172.65
1462.06104	CL 72:1 I CL 33:0_39:1	65.23	48.36	53.16	40.96	58.52	64.77	55.23	51.15
721.45844	CL 72:10 I CL 14:1_18:1_20:4_20:4	286.00	216.22	170.31	165.86	138.10	102.38	83.07	33.77
728.51807	CL 72:3 I CL 18:0_18:1_18:1_18:1	2373.35	2280.30	1494.22	2021.19	4740.27	6999.76	2690.49	4052.51
726.50574	CL 72:5 I CL 18:1_18:1_18:1_18:2	191.70	127.67	1652.61	2956.08	639.93	1829.10	462.53	523.00
725.48932	CL 72:6 I CL 18:0_18:2_18:2_18:2	334.01	259.68	179.22	178.20	199.86	123.98	134.63	41.85
725.48816	CL 72:6 I CL 18:1_18:1_18:2_18:2	152.35	148.20	488.11	500.42	179.71	173.87	117.80	81.91
724.48566	CL 72:7 I CL 18:1_18:2_18:2_18:2	177.55	199.11	348.34	324.55	183.98	166.57	189.53	86.25
723.47534	CL 72:8 I CL 18:2_18:2_18:2_18:2	303.15	457.13	180.87	146.12	176.98	111.66	110.55	26.91

Average m/z	Metabolite name	CTR		SCH		NF		PNF	
		mean	dev	mean	dev	mean	dev	mean	dev
722.45929	CL 72:9 I CL 18:0_14:1_20:4_20:4	112.75	109.88	40.27	33.45	63.51	91.08	47.59	18.28
735.52124	CL 73:3 I CL 28:0_15:1_15:1_15:1	87.77	94.68	242.59	405.91	67.09	170.96	115.23	77.01
1470.0249	CL 73:4 I CL 35:1_38:3	74.07	93.06	23.14	16.81	43.24	25.96	26.98	24.87
733.51752	CL 73:5 I CL 16:0_18:0_18:0_21:5	611.24	697.90	1239.53	2037.62	270.32	596.69	745.21	659.84
732.51819	CL 73:6 I CL 16:0_18:1_18:1_21:4	104.64	154.17	99.68	98.63	58.31	62.15	61.94	55.93
731.49146	CL 73:7 I CL 16:0_18:0_17:1_22:6	34.04	29.15	163.00	309.11	18.81	32.97	51.26	39.55
731.50061	CL 73:7 I CL 17:1_18:1_18:1_20:4	171.57	256.97	101.50	118.16	32.08	46.06	92.62	68.79
730.50201	CL 73:8 I CL 16:1_18:1_18:1_21:5	374.49	660.36	101.93	134.11	57.27	76.26	210.58	181.62
745.55255	CL 74:0 I CL 16:0_16:0_16:0_26:0	1842.14	1091.13	729.28	384.70	1626.95	561.65	1016.27	425.61
733.45129	CL 74:12 I CL 14:0_20:4_20:4_20:4	52.98	34.33	273.26	594.23	35.81	47.53	53.28	38.53
1488.10095	CL 74:2 I CL 35:1_39:1	239.69	124.23	154.85	125.92	181.17	186.45	156.92	138.44
1486.07751	CL 74:3 I CL 33:0_41:3	500.16	349.74	291.10	223.86	383.43	477.60	368.90	405.05
739.50909	CL 74:6 I CL 16:0_20:0_18:2_20:4	420.62	254.42	340.35	331.06	782.99	1636.48	411.23	412.79
1480.03027	CL 74:6 I CL 38:1_36:5	117.76	90.83	45.21	35.79	60.94	87.28	49.02	43.69
738.49774	CL 74:7 I CL 18:1_18:1_18:2_20:3	46.47	45.68	118.29	112.41	52.11	52.00	34.65	20.20
1478.01636	CL 74:7 I CL 34:3_40:4	174.68	209.10	27.06	24.09	22.96	12.80	29.73	23.34
1477.99939	CL 74:7 I CL 38:2_36:5	44.21	42.49	97.62	158.98	61.66	66.69	22.70	17.11
737.49054	CL 74:8 I CL 16:0_16:0_22:3_20:5	251.08	224.89	69.10	49.39	142.94	152.91	90.30	41.20
737.48645	CL 74:8 I CL 18:1_18:2_18:2_20:3	82.79	103.71	123.71	132.99	92.24	145.61	53.21	22.82
736.48157	CL 74:9 I CL 18:2_18:2_18:2_20:3	268.73	408.18	128.88	125.42	112.05	116.28	51.42	16.84
739.4505	CL 75:13 I CL 15:1_20:4_20:4_20:4	132.94	203.89	92.41	112.27	15.80	18.06	29.41	35.03
1498.05493	CL 75:4 I CL 41:0_34:4	1555.10	2099.49	482.26	390.76	401.61	270.41	503.55	477.37
1490.04175	CL 75:8 I CL 38:1_37:7	53.96	34.04	22.55	15.92	52.51	62.73	31.47	23.36
759.56702	CL 76:0 I CL 16:0_16:0_16:0_28:0	133.83	213.07	726.83	2008.32	131.09	98.68	136.53	106.54
749.4873	CL 76:10 I CL 18:2_18:2_20:3_20:3	62.07	94.31	22.87	21.55	38.30	82.94	11.00	0.57

**Average** *m/z*	**Metabolite name**	**CTR**		**SCH**		**NF**		**PNF**	
		mean	***dev***	***mean***	***dev***	***mean***	***dev***	***mean***	***dev***
756.55988	CL 76:3 | CL 18:0_18:0_18:0_22:3	1040.24	1753.45	272.36	178.59	1134.90	1383.46	1614.27	2344.36
756.56244	CL 76:3 | CL 28:0_16:1_16:1_16:1	820.02	922.62	847.94	1339.50	194.09	237.34	237.42	171.25
754.53497	CL 76:5 | CL 18:0_18:0_18:1_22:4	706.51	624.34	1559.71	3146.92	2225.02	2999.47	1381.73	2056.49
752.52112	CL 76:7 | CL 18:1_18:1_18:2_22:3	264.15	329.46	187.25	160.72	265.14	397.51	152.34	130.07
751.50574	CL 76:8 | CL 18:0_18:2_20:2_20:4	9.95	6.95	18.66	18.64	9.49	9.19	7.29	1.98
765.57227	CL 77:1 | CL 16:0_16:0_27:0_18:1	3161.55	2480.66	2249.42	1294.29	2997.04	1564.66	2092.47	1752.31
763.55145	CL 77:3 | CL 28:0_16:1_16:1_17:1	2677.90	3632.24	3880.16	9083.29	4155.97	8974.31	3115.55	3149.43
759.53723	CL 77:7 | CL 16:0_24:0_18:1_19:6	323.96	499.44	82.15	56.65	122.35	135.41	112.69	106.56
773.58514	CL 78:0 | CL 18:0_18:0_18:0_24:0	535.39	376.59	275.49	131.50	561.51	219.47	327.40	139.23
770.57428	CL 78:3 | CL 24:0_18:1_18:1_18:1	2011.67	2844.58	14155.44	30819.09	1245.18	1235.74	3439.89	4751.81
769.54675	CL 78:4 | CL 16:0_16:0_26:0_20:4	34.08	22.24	53.60	98.32	55.17	123.14	14.63	4.95
769.55518	CL 78:4 | CL 24:0_18:1_18:1_18:2	4497.48	5689.31	1086.73	664.58	2134.09	1711.54	1730.60	1143.13
768.5415	CL 78:5 | CL 24:0_18:1_18:2_18:2	1066.12	976.93	514.40	328.09	814.37	774.06	468.18	293.35
766.53094	CL 78:7 | CL 24:0_16:1_18:2_20:4	696.46	827.35	196.66	164.63	1655.14	4866.86	129.13	86.98
765.53821	CL 78:8 | CL 14:0_24:0_20:4_20:4	388.79	339.26	252.68	127.46	334.06	203.82	256.12	45.93
765.52551	CL 78:8 | CL 18:0_18:1_21:3_21:4	245.43	235.33	111.27	78.12	139.66	272.67	74.81	46.56
764.52106	CL 78:9 | CL 18:0_16:1_21:4_23:4	481.85	413.64	238.13	161.78	282.85	529.74	146.21	83.11
772.53937	CL 79:8 | CL 17:0_18:0_22:4_22:4	418.04	220.37	244.86	242.84	602.00	1210.58	102.92	46.63
777.53833	CL 80:10 | CL 16:0_24:0_20:5_20:5	67.21	61.86	73.23	60.63	42.95	53.86	52.65	38.15
784.58929	CL 80:3 | CL 26:0_18:1_18:1_18:1	40.31	51.63	291.60	642.00	29.60	25.59	57.06	74.05
782.55408	CL 80:5 | CL 26:0_18:1_18:2_18:2	66.49	60.59	141.43	242.65	58.69	56.44	59.61	53.75
781.55133	CL 80:6 | CL 26:0_18:2_18:2_18:2	105.03	107.41	416.93	783.32	160.40	181.44	206.01	213.66
779.55243	CL 80:8 | CL 18:0_18:0_22:4_22:4	399.53	338.71	378.39	480.79	241.69	205.76	166.94	53.18
782.52466	CL 81:12 | CL 15:1_22:3_22:4_22:4	147.16	126.10	65.32	47.47	304.72	270.45	104.22	31.89
801.60242	CL 82:0 | CL 18:0_18:0_18:0_28:0	3185.17	2100.43	7103.00	15978.67	2643.63	956.88	1889.58	749.60

Average *m/z*	Metabolite name	CTR		SCH		NF		PNF	
		mean	*dev*	*mean*	*dev*	*mean*	*dev*	*mean*	*dev*
784.49524	CL 82:17|CL 16:2_22:5_22:5_22:5	149.60	72.77	85.93	68.57	278.42	222.95	136.74	113.53
799.60724	CL 82:2|CL 18:0_18:0_28:0_18:2	174.32	225.52	2172.29	5092.30	163.83	152.29	296.27	381.69
797.59174	CL 82:4|CL 18:0_18:0_26:0_20:4	4208.61	3706.03	2671.22	4946.50	4461.15	4000.73	1295.43	509.06
793.54401	CL 82:8|CL 16:0_26:0_20:4_20:4	6982.97	7573.31	1771.64	1187.82	2138.78	2104.89	1413.27	538.18
793.55518	CL 82:8|CL 18:0_24:0_20:4_20:4	4246.16	5850.74	2001.17	1275.63	2757.35	2104.47	1581.98	319.99
792.55054	CL 82:9|CL 24:0_18:2_18:2_22:5	661.69	492.89	326.04	170.83	423.23	190.12	280.56	124.19
795.53607	CL 83:13|CL 17:1_22:4_22:4_22:4	426.61	281.55	206.67	120.34	306.52	144.61	136.78	24.47
791.48492	CL 83:17|CL 17:2_22:5_22:5_22:5	116.74	55.76	94.45	80.02	244.01	140.89	74.17	38.67
803.53864	CL 84:12|CL 24:0_20:4_20:4_20:4	112.79	131.56	43.94	25.47	62.84	50.88	44.74	39.17
802.54779	CL 84:13|CL 18:1_22:4_22:4_22:4	532.77	262.50	791.86	1540.71	738.58	989.82	387.92	274.08
800.5235	CL 84:15|CL 18:0_22:3_22:6_22:6	3124.17	5367.33	428.38	215.76	977.33	923.14	464.39	271.05
807.54779	CL 84:8|CL 28:0_18:2_18:2_20:4	662.43	867.91	1044.78	1997.37	925.08	1497.94	858.30	882.89
817.56415	CL 86:12|CL 26:0_20:4_20:4_20:4	789.66	548.62	971.16	1006.26	1134.27	936.17	727.79	524.55
814.54871	CL 86:15|CL 24:0_18:3_22:6_22:6	393.76	389.54	393.69	418.33	745.14	1541.46	247.89	94.33
814.54694	CL 86:15|CL 26:0_20:5_20:5_20:5	122.28	67.65	1155.01	2533.66	247.93	294.25	92.18	42.11
821.60577	CL 86:8|CL 18:0_24:0_22:4_22:4	222.26	148.93	159.20	98.52	330.97	264.42	171.88	59.69
820.56128	CL 86:9|CL 28:0_18:1_20:4_20:4	521.50	366.95	283.01	174.53	3474.69	9050.42	634.60	411.26
822.51794	CL 88:21|CL 22:5_22:5_22:5_22:6	252.88	197.30	93.76	155.18	64.82	48.27	71.26	69.65
821.4917	CL 88:22|CL 22:5_22:5_22:6_22:6	1189.18	2290.07	28.09	21.29	22.37	17.04	10.40	4.07
835.59692	CL 88:8|CL 18:0_26:0_20:4_24:4	114.56	135.36	89.11	106.60	235.98	145.36	175.98	75.01
846.58289	CL 90:11|CL 18:0_28:0_22:5_22:6	172.17	158.76	330.59	698.33	91.51	94.95	62.69	25.56
850.61945	CL 90:7|CL 24:0_24:0_22:3_20:4	196.06	271.39	85.78	88.75	203.80	520.84	62.42	62.40
884.71338	CL 94:1|CL 26:0_26:0_26:0_16:1	1845.27	2427.93	1266.91	1522.26	2247.22	2976.19	3511.62	4192.30
227.20383	FA 14:0	1449.82	986.82	1397.34	1762.56	1119.17	1753.40	562.09	123.08
243.19939	FA 14:0;(2OH)	23.72	20.36	114.19	256.39	29.22	31.21	12.43	4.78

Average *m/z*	Metabolite name	CTR		SCH		NF		PNF	
		mean	*dev*	*mean*	*dev*	*mean*	*dev*	*mean*	*dev*
243.19717	FA 14:0;O	23.64	20.44	110.21	242.50	29.30	31.27	12.36	4.79
225.18591	FA 14:1	148.31	206.70	76.13	168.39	63.44	124.94	38.06	60.32
257.17511	FA 14:1;2O	44.71	29.89	97.67	218.10	45.63	22.22	44.06	36.74
241.21684	FA 15:0	274.09	217.99	191.89	165.27	797.32	2150.86	103.79	18.71
257.21307	FA 15:0;(2OH)	9.76	5.19	442.86	1307.84	5.23	4.39	3.90	1.97
239.20146	FA 15:1	59.10	54.14	39.56	46.53	148.91	424.18	18.81	3.09
255.23366	FA 16:0	98809.33	81237.26	87439.34	121522.81	66548.53	54669.24	46934.56	15033.06
271.22446	FA 16:0;(2OH)	169.27	164.41	132.94	127.30	151.11	136.08	92.64	25.97
287.22485	FA 16:0;2O	49.40	50.20	159.70	332.87	30.02	23.95	32.88	25.40
271.22638	FA 16:0;O	346.06	274.95	1110.78	2013.24	2160.70	5593.59	472.82	181.71
253.21693	FA 16:1	7993.12	9277.98	9133.61	14912.25	1840.57	1704.65	2017.82	2459.26
285.20767	FA 16:1;2O	779.31	806.96	205.83	92.76	233.58	348.50	282.86	84.99
269.21347	FA 16:1;O	381.50	342.14	451.44	996.24	189.64	159.89	154.16	58.25
251.20088	FA 16:2	64.39	46.12	1571.58	4414.84	33.98	16.45	16.07	6.02
263.16788	FA 16:4;(2OH)	42.69	37.00	98.75	243.29	17.73	17.42	15.36	4.99
269.24887	FA 17:0	398.86	448.88	504.19	754.53	617.84	1406.50	150.09	33.50
285.24301	FA 17:0;(2OH)	30.88	39.06	98.48	244.12	496.64	1520.12	20.37	6.29
267.23251	FA 17:1	374.50	297.96	943.02	1775.63	378.05	848.67	102.57	26.72
265.21408	FA 17:2	9.61	7.59	17.96	22.68	37.46	107.82	2.74	0.95
281.2113	FA 17:2;O	91.62	129.67	204.25	388.53	59.48	79.15	8.42	10.44
279.19519	FA 17:3;O	404.77	487.82	1039.52	2024.60	245.10	318.07	15.64	11.38
261.18723	FA 17:4	6.03	6.70	22.24	65.04	2.34	2.66	1.97	1.05
277.18069	FA 17:4;O	20.61	24.26	8451.61	25034.18	9.79	15.25	14.89	7.08
283.2627	FA 18:0	119945.46	124985.50	100917.70	126684.59	74685.44	65882.59	56912.30	18138.25
299.25711	FA 18:0;(2OH)	223.89	227.20	689.95	1599.18	1051.98	2838.89	182.77	33.82
315.25397	FA 18:0;2O	478.98	511.28	683.77	1180.53	285.28	334.69	286.55	219.35

**Average** *m/z*	**Metabolite name**	**CTR**		**SCH**		**NF**		**PNF**	
		mean	*dev*	*mean*	*dev*	*mean*	*dev*	*mean*	*dev*
299.25775	FA 18:0;O	1090.26	1052.62	1490.79	3481.27	406.01	422.41	416.70	173.26
281.24841	FA 18:1	220841.33	198187.87	382603.00	730466.07	81322.79	61281.78	44347.97	18758.28
297.24338	FA 18:1;(2OH)	212.68	180.29	317.53	674.62	156.82	195.10	101.23	20.16
313.23868	FA 18:1;2O	131.52	113.88	148.11	238.71	83.57	77.39	75.39	69.23
297.24347	FA 18:1;O	449.33	237.11	450.00	387.97	347.50	486.60	511.64	636.76
279.23242	FA 18:2	59145.50	64852.65	59589.15	87988.06	29706.30	38608.37	12651.03	7070.09
295.2294	FA 18:2;(2OH)	178.03	212.56	38.26	43.92	44.79	51.10	24.34	32.15
311.22501	FA 18:2;2O	120.71	70.67	91.26	81.12	156.63	90.86	228.72	208.24
295.2261	FA 18:2;O	562.06	593.59	627.99	967.46	3558.68	10707.00	115.79	158.82
277.21744	FA 18:3	943.29	868.57	1383.10	1939.17	506.20	333.56	202.53	160.95
341.18744	FA 18:3;4O	44.79	41.17	3820.71	11538.11	25.69	27.52	28.24	8.25
293.21036	FA 18:3;O	27.44	23.18	30.00	57.83	61.13	148.11	9.58	8.65
275.21124	FA 18:4	42.03	63.07	242.84	696.87	6.24	10.90	5.53	2.22
273.18881	FA 18:5	375.48	362.16	122.90	111.77	87.89	133.85	121.41	29.60
297.28049	FA 19:0	88.78	102.86	84.97	109.55	161.91	325.52	42.74	13.67
295.26346	FA 19:1	197.47	185.77	703.85	1562.80	169.89	325.27	57.10	28.99
293.24838	FA 19:2	38.81	41.37	146.24	271.98	38.71	87.08	8.93	3.49
325.2359	FA 19:2;2O	318.88	335.42	352.94	571.50	159.86	193.78	71.24	37.50
291.22974	FA 19:3	26.77	26.88	425.01	1226.92	57.44	148.47	7.64	5.31
307.2251	FA 19:3;O	41.54	49.10	88.43	160.97	28.09	42.15	2.14	1.65
289.21368	FA 19:4	8.81	6.53	46.14	88.25	7.31	12.02	3.37	1.19
305.20972	FA 19:4;O	3.43	4.05	59.46	189.40	0.91	0.71	0.74	0.17
311.29428	FA 20:0	1246.41	1265.21	1003.50	1218.97	1016.59	1682.39	637.17	180.92
309.28021	FA 20:1	3608.07	3442.20	24654.13	56483.32	1237.12	1025.04	950.75	677.16
307.2626	FA 20:2	3686.77	4475.36	15029.19	33901.86	1216.08	1065.50	1211.42	966.60
355.24554	FA 20:2;3O	46.26	66.20	43.21	78.87	67.20	184.28	4.53	2.23
323.25766	FA 20:2;O	30.79	30.52	60.19	83.52	98.39	244.06	4.50	3.62

**Average** *m/z*	**Metabolite name**	**CTR**		**SCH**		**NF**		**PNF**	
		mean	***dev***	***mean***	***dev***	***mean***	***dev***	***mean***	***dev***
305.24948	FA 20:3	37313.15	59042.91	55503.46	115255.91	5909.01	5412.00	3719.14	3199.90
337.23737	FA 20:3;2O	162.52	124.76	91.55	83.94	109.84	77.04	77.26	22.61
353.22278	FA 20:3;3O	209.94	289.06	1784.55	5753.67	64.72	118.27	64.76	46.83
369.22638	FA 20:3;4O	1555.47	2390.90	1034.72	1290.32	482.06	599.36	61.87	26.25
321.24069	FA 20:3;O	202.66	323.90	115.88	201.33	506.59	1408.62	7.13	6.82
303.23169	FA 20:4	791140.07	1180482.19	1010215.82	1980468.52	132260.21	96783.12	84969.78	52870.38
319.22595	FA 20:4;(2OH)	38.03	60.56	57.78	96.61	27.20	66.37	3.57	0.42
335.22202	FA 20:4;2O	234.85	349.79	113.73	242.67	104.64	225.35	11.06	5.41
351.215	FA 20:4;3O	408.39	469.30	401.06	423.22	926.72	2527.39	26.82	19.41
319.22705	FA 20:4;O	3898.16	6068.66	958.81	1666.45	4749.45	10336.35	95.91	128.30
301.2168	FA 20:5	4306.48	5795.44	8428.03	13726.01	2111.90	3653.73	1003.78	1135.89
317.20972	FA 20:5;(2OH)	63.82	95.40	40.98	81.94	99.98	174.09	3.76	3.60
333.2038	FA 20:5;2O	115.42	99.11	132.99	136.36	318.35	734.57	14.50	8.51
349.20151	FA 20:5;3O	110.62	169.01	105.19	267.26	38.07	62.91	9.31	5.22
317.21173	FA 20:5;O	79.93	100.01	114.46	153.16	28.08	31.25	4.65	4.55
325.30826	FA 21:0	191.54	212.70	363.21	762.34	340.05	766.09	124.40	124.71
341.30179	FA 21:0;(2OH)	1.23	1.29	2.16	4.89	39.31	119.56	0.75	0.26
341.30365	FA 21:0;O	166.52	126.45	168.77	466.71	82.18	191.32	67.59	117.13
323.29147	FA 21:1	10.09	8.14	67.66	146.68	13.94	32.40	4.62	2.74
321.2774	FA 21:2	4.94	4.65	41.56	91.18	8.26	20.32	1.57	0.85
319.26181	FA 21:3	26.57	24.16	457.30	1283.26	18.07	37.65	5.34	2.39
317.2467	FA 21:4	9.40	8.82	378.22	887.34	3.56	5.00	2.46	1.35
315.24515	FA 21:5	49.59	69.45	318.16	921.81	10.64	15.67	9.47	2.65
339.32288	FA 22:0	2775.33	2502.18	1571.29	2022.92	4555.66	11719.99	1076.55	304.61
403.30548	FA 22:0;4O	165.81	196.84	686.82	1509.73	120.89	89.40	43.19	5.13
337.30853	FA 22:1	415.12	463.00	1639.20	3590.98	251.44	318.69	145.85	113.84
335.29318	FA 22:2	231.48	201.31	1261.64	2588.94	110.27	129.38	86.49	56.13

Average *m/z*	Metabolite name	CTR		SCH		NF		PNF	
		mean	***dev***	***mean***	***dev***	***mean***	***dev***	***mean***	***dev***
333.27701	FA 22:3	586.48	551.54	5100.40	13317.30	163.08	204.08	75.75	32.16
365.26904	FA 22:3;2O	24.48	7.36	20.62	25.28	63.06	39.37	77.59	73.66
381.26105	FA 22:3;3O	14.92	12.65	58.62	89.94	26.57	57.21	2.54	1.75
349.27142	FA 22:3;O	21.19	23.00	87.32	223.60	8.78	13.51	7.53	2.13
331.26373	FA 22:4	46646.25	63984.10	114747.30	250912.23	6303.99	5703.72	3782.98	2338.08
347.25723	FA 22:4;O	67.14	90.10	45.48	58.33	70.72	104.81	4.83	6.39
329.24817	FA 22:5	26227.59	37314.44	63357.40	130178.59	2464.54	2308.91	1778.83	881.98
409.22495	FA 22:5;4O	685.98	1093.20	240.37	287.78	258.71	386.82	137.29	70.63
345.24054	FA 22:5;O	110.03	160.00	63.82	103.80	97.20	210.38	5.44	5.80
327.23157	FA 22:6	161453.26	240087.69	366129.54	929409.60	19579.06	12346.85	15860.58	13711.81
343.22485	FA 22:6;(2OH)	344.21	526.06	141.39	275.99	390.05	853.75	13.27	10.74
391.20468	FA 22:6;4O	1752.18	1012.02	849.84	545.72	1444.55	754.16	861.08	266.20
353.33838	FA 23:0	155.85	113.18	109.96	140.13	1176.14	3160.64	92.76	35.43
369.33295	FA 23:0;(2OH)	6.66	6.72	23.78	36.14	338.17	1048.54	6.68	4.00
351.32489	FA 23:1	48.17	42.30	1137.70	2798.75	52.14	96.76	45.73	52.81
367.35345	FA 24:0	876.23	582.76	696.76	749.17	9695.80	28626.30	556.01	186.06
383.34821	FA 24:0;(2OH)	156.55	158.93	301.13	356.24	15470.39	48350.88	156.45	84.14
365.33923	FA 24:1	1718.19	1674.13	4483.43	8215.95	1359.44	1889.02	639.80	353.02
381.33487	FA 24:1;O	3.88	2.91	36.47	88.27	3.29	1.55	1.91	0.50
363.32513	FA 24:2	71.58	88.92	312.03	563.73	52.44	62.32	32.93	23.33
361.31097	FA 24:3	90.53	112.13	737.59	1814.85	16.42	17.09	8.96	5.45
359.293	FA 24:4	1131.41	1168.17	4340.11	10212.57	621.95	594.37	391.07	293.76
357.2774	FA 24:5	575.74	621.71	2608.48	5282.93	153.62	123.75	126.13	96.33
355.26157	FA 24:6	485.04	630.60	1746.68	4443.59	128.31	87.09	143.09	159.03
381.36877	FA 25:0	97.30	62.21	56.39	37.19	2755.69	8371.70	97.93	92.23
397.36542	FA 25:0;O	28.06	21.97	32.03	36.02	2820.47	8809.00	22.97	3.98

**Average** *m/z*	**Metabolite name**	**CTR**		**SCH**		**NF**		**PNF**	
		mean	***dev***	***mean***	***dev***	***mean***	***dev***	***mean***	***dev***
379.35379	FA 25:1	14.96	10.41	53.46	89.66	33.65	79.08	11.16	3.34
395.38599	FA 26:0	1088.20	810.22	690.38	387.88	11860.48	35718.63	637.05	168.70
411.37933	FA 26:0;(2OH)	10.06	4.18	16.03	21.61	3653.02	11447.86	14.47	10.03
411.38211	FA 26:0;O	32.29	24.22	62.30	49.48	74.99	40.02	92.32	50.70
393.36938	FA 26:1	109.68	77.29	404.01	641.69	165.02	316.63	65.52	53.79
409.36203	FA 26:1;(2OH)	7.54	6.12	18.00	22.07	399.66	1247.39	5.54	2.60
391.35165	FA 26:2	39.45	35.47	188.23	274.97	31.82	36.77	24.17	22.26
389.33917	FA 26:3	87.10	102.09	662.99	1311.55	32.49	38.89	21.05	11.74
387.32379	FA 26:4	113.76	127.70	415.20	808.71	73.88	73.97	45.30	33.05
385.30923	FA 26:5	210.05	197.56	521.16	712.40	69.88	59.36	56.78	45.33
383.2916	FA 26:6	63.62	82.25	163.24	304.90	25.73	21.59	28.75	31.53
381.27887	FA 26:7	7698.02	6501.54	317.70	326.03	817.06	1006.07	838.85	1270.11
397.27167	FA 26:7;O	287.41	201.04	27.52	42.30	41.71	57.14	147.39	292.01
409.39737	FA 27:0	174.18	200.46	50.85	21.52	2688.23	8272.00	63.64	59.54
425.39191	FA 27:0;(2OH)	1.33	1.18	2.20	2.97	358.11	1117.73	2.11	1.93
423.41602	FA 28:0	63.87	35.47	110.42	277.29	148.14	168.02	44.90	37.14
439.41037	FA 28:0;O	18.49	20.59	44.13	108.19	310.26	951.36	17.41	4.98
421.39917	FA 28:1	16.65	10.49	49.74	53.80	137.78	404.87	9.96	3.93
415.35641	FA 28:4	10.44	10.24	59.52	84.62	11.00	14.31	7.24	4.54
413.34021	FA 28:5	27.79	27.29	65.95	70.68	14.78	21.79	12.72	10.34
427.31778	FA 28:6;O	15.15	15.12	45.46	99.94	33.34	64.62	6.97	3.11
409.30814	FA 28:7	87280.68	120224.08	201512.78	543645.52	22270.46	37453.15	12810.27	3694.72
425.30124	FA 28:7;O	373.30	331.48	5271.66	15346.77	143.68	221.11	176.14	83.61
437.4299	FA 29:0	24.61	18.99	11.72	6.73	532.04	1606.22	15.09	6.78
435.41272	FA 29:1	10.68	12.92	7.15	8.45	43.03	126.03	5.02	4.45
451.44427	FA 30:0	836.80	704.81	488.58	305.70	1590.80	3646.93	550.42	284.88

Average m/z	Metabolite name	CTR		SCH		NF		PNF	
		mean	dev	mean	dev	mean	dev	mean	dev
435.31827	FA 30:8	116.23	178.11	503.26	1446.51	12.26	18.29	16.13	10.77
465.46078	FA 31:0	157.55	137.19	103.38	176.00	192.60	159.15	81.02	47.66
463.44858	FA 31:1	21.70	32.26	23.62	60.47	4.10	4.61	5.87	3.27
479.48108	FA 32:0	709.70	672.95	525.93	419.69	536.58	541.06	485.57	238.02
471.42886	FA 32:4	2.30	1.66	14.56	40.00	1.24	1.62	2.08	0.80
469.41086	FA 32:5	6.45	5.41	29.35	82.59	2.10	3.42	2.36	0.53
463.36765	FA 32:8	6.12	4.69	7.09	9.23	90.90	272.74	6.21	7.69
493.49066	FA 33:0	148.33	119.95	93.77	157.09	138.65	105.31	65.41	26.32
507.51108	FA 34:0	475.90	333.14	487.59	245.36	412.97	245.62	464.94	47.34
505.48941	FA 34:1	14.25	10.10	25.07	39.16	285.62	850.29	12.95	4.27
521.5224	FA 35:0	21.95	15.61	57.13	87.05	16.95	16.79	26.13	7.57
519.51025	FA 35:1	4.35	4.63	24.79	59.52	18.42	49.62	3.65	1.26
535.53571	FA 36:0	318.81	282.88	261.94	177.66	197.00	108.02	191.39	74.57
533.52338	FA 36:1	10.05	6.51	27.09	41.69	198.50	592.85	13.39	4.43
531.50867	FA 36:2	3.93	3.30	19.76	44.33	59.94	179.50	3.56	0.20
527.48785	FA 36:4	8.81	7.94	42.06	109.82	7.63	11.29	9.25	1.45
525.47083	FA 36:5	16.57	13.69	98.90	272.13	6.11	8.63	9.01	2.62
521.42834	FA 36:7	31.34	49.75	46.77	120.67	4.82	5.11	3.55	2.52
519.43182	FA 36:8	28.53	28.40	14.13	11.97	42.51	99.05	41.70	63.47
549.55194	FA 37:0	145.45	143.88	117.91	227.44	197.43	175.34	79.56	43.65
563.56586	FA 38:0	165.30	148.48	134.51	123.18	108.60	67.02	88.42	45.11
561.55359	FA 38:1	7.92	5.09	22.65	41.69	68.54	200.61	6.18	3.43
543.43054	FA 38:10	5.86	4.43	41.79	120.37	7.43	6.77	2.66	1.97
555.51733	FA 38:4	2.86	2.30	23.66	63.46	1.34	1.70	2.34	1.54
553.48663	FA 38:5	73.49	91.21	99.44	234.07	47.33	30.66	23.76	5.87
547.4613	FA 38:8	33.19	36.78	17.79	20.57	23.64	29.69	38.26	55.21

Average m/z	Metabolite name	CTR		SCH		NF		PNF	
		mean	dev	mean	dev	mean	dev	mean	dev
577.58313	FA 39:0	94.36	96.80	103.58	200.34	105.09	80.40	52.38	21.30
591.59869	FA 40:0	104.69	103.84	131.88	272.24	137.67	115.31	62.33	32.57
589.58582	FA 40:1	2.80	2.86	25.27	65.38	1.31	2.02	1.59	0.64
571.46643	FA 40:10	241.22	175.62	238.10	322.90	391.93	262.02	112.27	42.34
569.44904	FA 40:11	58.45	39.09	125.64	143.08	99.98	53.02	65.86	28.06
575.49237	FA 40:8	29.31	30.10	9.72	14.75	20.29	36.24	17.55	25.52
619.63153	FA 42:0	85.78	85.42	122.60	235.68	88.56	60.49	58.56	31.52
599.49359	FA 42:10	849.86	431.68	2178.78	4096.90	1729.09	1287.56	632.76	220.25
597.48163	FA 42:11	183.69	106.97	282.78	446.38	248.49	82.69	94.49	46.56
609.56262	FA 42:5	8.22	7.93	47.42	132.16	3.80	2.80	3.40	1.15
601.50885	FA 42:9	1112.44	694.56	2891.95	4877.67	4096.42	4787.11	3730.54	2149.35
633.64581	FA 43:0	105.03	94.53	138.35	266.96	122.24	83.00	66.56	42.38
647.66309	FA 44:0	21.47	13.80	22.85	18.85	22.29	18.08	15.03	3.00
627.52368	FA 44:10	3246.79	1073.35	5174.13	5046.87	5158.56	2869.01	2004.12	1139.37
625.5127	FA 44:11	1548.15	904.15	7643.16	14641.58	1620.28	1611.55	841.03	532.96
623.48132	FA 44:12	46.97	50.25	37.74	36.46	18.86	14.76	9.07	3.82
631.53973	FA 44:8	40.45	63.26	14.19	20.49	23.90	14.67	9.95	10.74
629.54211	FA 44:9	65.75	50.59	258.24	355.75	518.64	597.70	96.76	58.73
1123.67749	GM3 32:1;2O	37.28	50.53	13.67	10.52	18.97	10.76	14.77	5.31
1153.70605	GM3 34:0;2O	47.22	52.14	22.15	23.38	16.85	12.26	19.40	13.77
1177.71973	GM3 36:2;2O	22.37	25.98	24.43	29.14	11.37	9.18	5.99	1.09
1235.79529	GM3 40:1;2O	200.06	187.50	114.20	72.88	398.12	618.61	246.05	337.98
1249.79797	GM3 41:1;2O	33.08	18.99	67.00	61.92	67.29	83.55	31.45	21.93
1263.82471	GM3 42:1;2O	534.96	554.19	238.93	135.65	411.95	362.57	297.99	359.16
381.19531	LPA 14:0	145.30	235.04	39.94	43.98	40.73	56.44	22.19	10.91

Average m/z	Metabolite name	CTR		SCH		NF		PNF	
		mean	dev	mean	dev	mean	dev	mean	dev
395.22504	LPA 15:0	121.57	86.58	100.84	127.70	64.09	38.97	37.08	13.33
423.24292	LPA 17:0	56.99	89.62	12.86	8.32	24.27	36.91	12.23	4.73
435.24927	LPA 18:1	14.68	13.88	67.46	108.71	8.36	17.67	3.76	1.48
451.27609	LPA 19:0	1.80	1.01	336.65	803.65	1.39	1.20	0.98	0.55
485.26373	LPA 22:4	298.15	214.51	312.63	567.81	177.09	79.21	132.18	82.02
543.33759	LPA 26:3	3.81	2.76	43.14	108.83	4.72	3.53	2.33	0.87
537.29407	LPA 26:6	38.22	49.67	72.02	149.83	8.94	8.92	12.56	17.02
483.27036	LPG 16:0	934.87	1612.42	44.37	35.34	43.84	52.09	36.36	13.78
511.30399	LPG 18:0	1345.40	2449.57	43.52	37.46	55.05	77.14	30.10	12.56
509.28607	LPG 18:1	486.67	431.64	615.78	896.95	422.70	410.73	353.59	140.39
507.27023	LPG 18:2	554.67	823.45	441.14	509.67	197.93	224.71	163.48	98.19
537.31616	LPG 20:1	32.40	33.63	25.19	26.56	29.59	48.91	13.24	14.95
535.31476	LPG 20:2	11.88	13.07	34.14	65.07	7.52	5.58	6.91	4.17
531.26971	LPG 20:4	219.11	248.83	141.32	161.52	88.85	66.91	67.11	33.77
559.30023	LPG 22:4	47.38	34.34	54.07	59.58	29.90	23.85	19.05	6.19
557.28754	LPG 22:5	182.27	123.56	191.67	235.71	101.36	94.40	97.11	57.39
555.26715	LPG 22:6	600.37	837.60	337.21	356.16	291.17	181.60	286.33	212.61
441.24963	LPG O-14:0	159.36	105.46	95.88	86.74	121.25	61.80	82.57	39.47
479.27875	LPG O-17:2	19.24	8.83	25.75	24.28	21.46	8.43	16.61	5.76
495.30905	LPG O-18:1	31.04	40.64	1141.58	3150.58	9.56	12.03	6.05	2.99
521.33014	LPG O-20:2	9.32	9.05	86.98	220.75	2.56	2.42	2.34	0.53
533.33282	LPG O-21:3	12.15	10.94	117.18	303.43	4.23	3.29	4.59	2.78
595.34259	LPG O-26:7	3.45	2.10	30.73	95.27	3.09	3.83	2.09	0.89
599.31476	LPI 18:0	468.59	470.84	96.10	66.88	493.61	1202.36	55.38	29.61
597.30237	LPI 18:1	144.83	197.00	28.66	28.92	60.88	104.87	13.53	8.46

Average *m/z*	Metabolite name	CTR		SCH		NF		PNF	
		mean	*dev*	*mean*	*dev*	*mean*	*dev*	*mean*	*dev*
595.28253	LPI 18:2	33.47	29.38	22.00	25.22	16.90	21.84	7.85	2.53
621.29913	LPI 20:3	13.92	11.76	19.66	27.88	9.12	10.18	4.07	1.58
619.28381	LPI 20:4	347.01	517.82	218.89	475.80	60.64	57.56	32.68	21.03
496.26459	LPS 16:0	32.32	43.42	6.31	11.53	3.48	5.50	1.27	0.75
524.2981	LPS 18:0	540.86	632.24	22.73	30.96	64.02	164.20	7.88	6.39
522.28406	LPS 18:1	684.79	1178.06	178.87	319.70	97.76	257.71	11.00	9.56
520.26837	LPS 18:2	21.08	24.00	16.08	23.31	7.56	11.74	2.23	1.82
536.31342	LPS 19:1	13.05	17.65	153.16	463.64	4.86	3.87	12.06	12.01
546.28253	LPS 20:3	32.27	37.43	59.06	102.53	5.30	5.80	2.08	1.15
544.26465	LPS 20:4	69.52	95.52	26.08	33.54	11.60	21.43	2.69	1.35
572.29834	LPS 22:4	43.94	66.87	33.51	58.04	4.37	3.51	2.43	0.99
570.28259	LPS 22:5	73.35	110.67	21.89	17.55	11.62	10.11	9.42	7.48
568.2677	LPS 22:6	91.50	144.10	41.73	58.52	12.88	19.33	2.98	1.14
721.50323	PG 32:0 | PG 16:0_16:0	4488.20	7892.24	508.94	352.46	869.74	1032.09	566.34	259.57
719.47827	PG 32:1 | PG 16:0_16:1	707.29	910.42	569.28	448.94	357.46	634.93	361.78	197.55
747.50879	PG 34:1 | PG 16:0_18:1	12117.37	14594.41	6984.09	5086.92	7053.54	6109.59	4571.80	2426.02
745.50269	PG 34:2 | PG 16:0_18:2	545.27	680.23	289.84	240.98	293.34	319.25	177.37	50.25
745.49408	PG 34:2 | PG 16:1_18:1	474.00	420.42	1176.92	1002.16	317.12	351.52	493.41	388.26
743.4826	PG 34:3 | PG 16:1_18:2	47.86	37.85	61.58	46.74	48.03	42.43	47.57	14.04
759.52777	PG 35:2 | PG 17:1_18:1	548.51	452.67	2558.64	3862.74	358.58	428.36	355.98	350.77
757.49121	PG 35:3 | PG 17:1_18:2	4.68	3.61	10.98	17.05	8.52	20.16	4.23	3.73
777.55511	PG 36:0 | PG 18:0_18:0	11574.53	11025.33	4089.04	2168.21	5100.82	2355.88	3285.47	1602.92
775.54706	PG 36:1 | PG 18:0_18:1	9452.38	11180.37	3075.03	1542.83	4293.68	3459.91	2508.44	735.89
773.52252	PG 36:2 | PG 18:1_18:1	18097.27	15599.71	23063.83	28370.36	15091.87	23392.87	13261.01	12974.53
771.51971	PG 36:3 | PG 18:1_18:2	14531.05	12185.34	28285.94	33002.10	13515.11	14906.75	13184.39	8724.19
769.49884	PG 36:4 | PG 16:0_20:4	868.45	1189.40	347.90	253.27	214.87	102.71	199.70	66.89
769.49884	PG 36:4 | PG 18:2_18:2	1079.34	1235.38	831.28	927.84	735.29	1072.32	455.69	266.33

**Average** *m/z*	**Metabolite name**	**CTR**		**SCH**		**NF**		**PNF**	
		mean	*dev*	*mean*	*dev*	*mean*	*dev*	*mean*	*dev*
815.47205	PG 36:5;3O|PG 18:3_18:2;3O	32.78	57.53	29.39	64.70	12.44	17.74	7.28	4.19
767.4751	PG 36:5|PG 16:1_20:4	8.92	6.36	15.51	13.29	6.39	4.61	8.23	2.26
767.47516	PG 36:5|PG 18:2_18:3	7.97	8.54	17.94	35.76	8.10	14.56	3.93	2.77
785.51788	PG 37:3|PG 19:1_18:2	449.85	794.47	102.42	87.29	105.78	117.55	53.11	20.16
803.57458	PG 38:1|PG 18:0_20:1	602.53	447.13	339.24	240.59	614.53	677.21	316.59	132.72
801.55103	PG 38:2|PG 18:1_20:1	1062.52	1471.09	743.36	895.51	649.77	818.46	362.85	286.72
799.53961	PG 38:3|PG 18:0_20:3	321.97	392.17	75.70	41.27	142.82	86.93	64.83	10.12
799.53693	PG 38:3|PG 18:1_20:2	701.15	393.61	1177.68	2219.05	526.76	659.87	411.23	232.91
797.53217	PG 38:4|PG 18:0_20:4	741.68	481.16	1228.48	1740.81	727.54	728.63	554.89	82.34
797.53027	PG 38:4|PG 18:1_20:3	771.83	497.81	1170.19	1521.96	681.35	588.83	501.36	79.49
797.52179	PG 38:4|PG 18:2_20:2	2116.90	2203.58	6001.34	11543.07	2031.90	2238.14	2728.40	2724.05
795.51361	PG 38:5|PG 18:1_20:4	4317.76	3352.31	6066.60	7322.11	2853.78	2998.68	2244.98	799.31
795.50635	PG 38:5|PG 18:2_20:3	72.45	74.15	122.51	109.80	52.45	45.23	41.27	29.64
793.49219	PG 38:6|PG 16:0_22:6	936.08	1557.19	250.98	191.14	141.50	148.15	121.04	67.64
793.49121	PG 38:6|PG 18:2_20:4	280.07	344.61	284.25	354.37	120.30	162.81	108.73	126.64
791.47974	PG 38:7|PG 16:1_22:6	14.40	14.85	25.17	41.63	11.46	10.41	13.67	11.94
791.47772	PG 38:7|PG 18:2_20:5	11.75	14.64	15.96	27.44	6.94	9.62	3.94	3.34
809.52997	PG 39:5|PG 18:1_21:4	117.37	101.03	128.70	103.14	59.13	72.94	60.33	51.18
829.60022	PG 40:2|PG 20:1_20:1	90.23	52.16	114.57	118.76	154.38	76.35	130.38	62.69
825.55438	PG 40:4|PG 18:1_22:3	1402.57	2459.11	278.09	278.73	293.55	181.53	195.13	137.11
823.54755	PG 40:5|PG 18:1_22:4	3735.32	2936.07	4774.70	6373.23	2093.17	2897.04	1699.15	1671.94
823.53241	PG 40:5|PG 20:2_20:3	279.18	242.83	468.08	396.02	181.68	184.85	168.81	104.45
821.53198	PG 40:6|PG 18:1_22:5	1890.58	1933.27	2885.26	2899.96	1392.06	1661.37	925.62	475.69
821.52002	PG 40:6|PG 18:2_22:4	92.30	89.56	218.46	344.15	67.11	61.91	56.45	44.35
819.50562	PG 40:7|PG 18:1_22:6	1426.90	2094.21	1194.09	2017.30	558.12	653.37	468.58	495.01
819.50592	PG 40:7|PG 18:2_22:5	198.77	187.19	255.19	252.99	159.04	102.39	147.20	67.27

**Average** *m/z*	**Metabolite name**	**CTR**		**SCH**		**NF**		**PNF**	
		mean	*dev*	*mean*	*dev*	*mean*	*dev*	*mean*	*dev*
819.51538	PG 40:7 | PG 20:3_20:4	59.26	68.52	55.90	66.57	21.94	17.47	15.29	14.39
817.50214	PG 40:8 | PG 18:2_22:6	641.62	1032.77	543.12	1011.21	258.81	320.60	286.27	376.15
817.49103	PG 40:8 | PG 20:4_20:4	332.34	451.07	268.11	242.64	158.00	131.86	124.23	68.43
841.49011	PG 42:10 | PG 20:4_22:6	475.45	729.62	216.27	265.67	82.44	77.08	89.26	114.77
839.47192	PG 42:11 | PG 20:5_22:6	94.30	117.66	57.73	54.28	35.88	23.48	23.99	13.15
851.58032	PG 42:5 | PG 20:1_22:4	1113.29	1808.72	271.26	215.17	308.27	221.51	265.00	211.32
849.55072	PG 42:6 | PG 20:2_22:4	143.64	106.37	206.74	200.41	91.69	44.70	82.45	51.48
847.53363	PG 42:7 | PG 20:2_22:5	412.95	556.99	894.63	1357.33	182.60	162.23	244.56	216.51
845.53027	PG 42:8 | PG 20:3_22:5	531.54	518.44	1027.76	979.40	432.85	381.62	468.13	252.46
845.51965	PG 42:8 | PG 20:4_22:4	77.57	74.37	95.37	116.30	33.09	22.67	36.74	38.10
843.50403	PG 42:9 | PG 20:3_22:6	110.29	164.83	76.61	95.46	26.87	24.02	24.13	27.86
843.51337	PG 42:9 | PG 20:4_22:5	194.27	223.46	123.38	124.33	38.79	25.45	55.02	55.19
869.53082	PG 44:10 | PG 22:4_22:6	137.23	163.02	71.55	70.58	44.48	33.62	38.15	25.29
869.5329	PG 44:10 | PG 22:5_22:5	1056.05	1000.35	983.67	1497.96	446.79	641.42	620.30	502.33
867.5213	PG 44:11 | PG 22:5_22:6	602.63	934.78	179.37	200.64	67.07	69.60	120.83	173.79
865.49097	PG 44:12 | PG 22:6_22:6	906.52	1606.65	426.81	724.68	136.01	164.44	198.29	302.75
873.55927	PG 44:8 | PG 22:4_22:4	372.05	300.99	427.75	718.16	134.63	98.93	122.87	117.87
871.54376	PG 44:9 | PG 22:4_22:5	440.19	463.40	725.97	712.84	162.94	159.09	347.32	446.49
937.6557	PG 48:4 | PG 24:2_24:2	80.05	40.17	122.23	132.54	80.73	42.12	66.39	15.87
705.50897	PG O-32:1 | PG O-16:1_16:0	22.44	16.06	65.34	76.78	32.81	37.65	19.23	4.64
703.48602	PG O-32:2 | PG O-16:1_16:1	8.51	4.30	49.96	59.66	12.19	17.10	8.68	5.67
731.52307	PG O-34:2 | PG O-16:1_18:1	80.54	47.54	223.33	246.38	133.60	103.80	92.45	55.24
731.53796	PG O-34:2 | PG O-18:2_16:0	377.37	296.65	319.08	249.67	624.59	1104.19	220.18	60.10
729.50604	PG O-34:3 | PG O-16:2_18:1	21.53	12.63	86.35	104.57	34.12	39.64	22.90	13.47
729.52643	PG O-34:3 | PG O-17:2_17:1	152.29	71.66	390.64	575.95	460.93	1161.38	168.54	123.66
727.48419	PG O-34:4 | PG O-16:1_18:3	23.27	20.00	45.38	77.17	17.74	16.04	21.01	11.15
727.50153	PG O-34:4 | PG O-16:2_18:2	125.86	144.78	74.51	109.56	140.13	225.45	59.46	43.53

Average *m/z*	Metabolite name	CTR		SCH		NF		PNF	
		mean	*dev*	*mean*	*dev*	*mean*	*dev*	*mean*	*dev*
761.57007	PG O-36:1 | PG O-18:1_18:0	1619.51	2674.40	414.59	537.01	837.46	865.07	1108.11	1349.40
759.54865	PG O-36:2 | PG O-18:1_18:1	101.44	53.04	204.67	147.19	96.95	58.89	132.08	76.76
757.53613	PG O-36:3 | PG O-18:1_18:2	56.61	32.13	146.11	96.54	129.05	80.15	92.17	82.54
755.52313	PG O-36:4 | PG O-18:2_18:2	37.85	33.62	78.35	64.55	36.04	33.26	36.33	32.58
753.50592	PG O-36:5 | PG O-18:3_18:2	85.87	75.11	98.42	117.66	60.08	102.93	38.05	12.49
753.51892	PG O-36:5 | PG O-20:4_16:1	154.79	166.42	147.47	253.81	142.46	243.13	105.06	74.83
789.60071	PG O-38:1 | PG O-20:1_18:0	296.71	249.16	64.41	53.73	145.15	147.68	212.64	194.19
781.5354	PG O-38:5 | PG O-18:1_20:4	260.71	308.12	227.58	219.46	145.50	121.23	97.55	51.66
817.6311	PG O-40:1 | PG O-20:1_20:0	62.16	90.01	9.90	9.04	42.91	45.33	61.18	61.98
805.52405	PG O-40:7 | PG O-18:1_22:6	208.16	175.27	102.02	89.38	110.76	77.43	64.03	48.05
807.49591	PI 32:1 | PI 16:0_16:1	125.72	202.84	28.88	41.95	25.28	28.35	31.46	30.71
835.53607	PI 34:1 | PI 16:0_18:1	7281.89	10845.24	1182.38	1758.87	1263.62	1116.21	1001.89	762.71
833.5144	PI 34:2 | PI 16:0_18:2	2646.64	3316.50	1533.74	2408.83	1173.23	1785.17	634.67	508.66
833.50214	PI 34:2 | PI 16:1_18:1	94.00	126.29	56.94	79.18	36.19	44.83	43.62	54.90
863.56042	PI 36:1 | PI 18:0_18:1	1197.84	1005.21	393.67	441.46	988.65	1208.37	339.27	161.17
861.5426	PI 36:2 | PI 18:1_18:1	6246.73	7392.34	1032.32	1048.63	2358.71	2449.64	1319.89	933.20
859.53107	PI 36:3 | PI 16:0_20:3	472.57	534.83	398.30	718.10	375.79	461.46	172.34	111.94
857.50452	PI 36:4	1039.51	923.98	869.69	1091.13	734.67	1314.39	438.33	317.81
857.51831	PI 36:4 | PI 16:0_20:4	2263.88	2143.63	1457.76	1863.75	2494.80	4530.83	1216.64	1381.80
857.51538	PI 36:4 | PI 18:2_18:2	45.05	39.27	37.98	39.58	38.25	24.41	23.28	16.18
855.50928	PI 36:5 | PI 16:0_20:5	411.80	346.10	245.11	217.83	261.88	146.68	213.25	99.95
855.49011	PI 36:5 | PI 16:1_20:4	11.28	13.46	10.45	12.55	23.39	41.14	12.61	17.02
871.53137	PI 37:4 | PI 17:0_20:4	860.29	767.91	540.74	783.48	575.16	628.20	217.59	78.82
887.56409	PI 38:3 | PI 18:0_20:3	2681.81	1644.60	2040.29	2673.96	2473.13	3250.80	612.22	238.18
885.53772	PI 38:4 | PI 18:0_20:4	70145.83	65204.75	21227.63	19904.48	48717.22	60798.04	14534.37	6530.27
883.53455	PI 38:5	27.87	27.39	14.74	20.27	14.59	9.93	9.27	7.32

**Average** *m/z*	**Metabolite name**	**CTR**		**SCH**		**NF**		**PNF**	
		mean	*dev*	*mean*	*dev*	*mean*	*dev*	*mean*	*dev*
883.52045	PI 38:5 I PI 18:1_20:4	86871.00	110337.70	63803.77	92371.97	41596.77	68430.54	28321.74	31569.60
881.51562	PI 38:6 I PI 16:0_22:6	207.45	209.50	101.41	99.19	78.04	68.93	48.83	20.11
881.51892	PI 38:6 I PI 18:2_20:4	60.96	69.36	52.44	70.39	63.29	84.17	22.96	24.21
913.56744	PI 40:4 I PI 18:0_22:4	830.10	592.83	282.41	246.37	690.80	909.39	179.48	94.89
911.56702	PI 40:5	209.53	139.09	177.81	279.24	75.17	45.17	45.07	13.28
909.53754	PI 40:6	135.14	107.59	149.45	187.04	253.51	475.56	75.36	53.38
909.53937	PI 40:6 I PI 18:1_22:5	139.22	151.86	187.95	332.76	55.50	51.04	45.24	39.55
907.5332	PI 40:7	88.07	85.95	44.69	45.31	52.92	65.05	18.25	12.39
905.51538	PI 40:8 I PI 20:4_20:4	75.94	67.58	47.53	57.09	51.28	37.24	23.80	21.25
933.52954	PI 42:8	154.24	221.76	52.23	67.64	36.31	34.75	28.60	22.54
953.52753	PI 44:12	195.88	85.53	46.35	45.82	268.04	286.23	43.35	43.59
679.38019	PI O-24:2	407.31	289.88	206.92	133.61	316.58	216.81	207.45	61.20
678.39642	PS 27:1;O I PS 10:0_17:1;O	101.92	158.58	9.48	6.94	21.35	48.45	5.52	3.82
734.49023	PS 32:0 I PS 16:0_16:0	121.42	95.69	31.28	23.65	57.40	105.53	19.37	7.75
732.48413	PS 32:1 I PS 16:0_16:1	48.23	90.23	3.65	5.13	5.53	8.82	28.99	54.37
760.50464	PS 34:1 I PS 16:0_18:1	4976.32	9066.96	344.95	525.76	883.93	2241.50	241.00	180.45
806.48798	PS 34:2;3O I PS 16:0_18:2;3O	213.95	220.26	53.26	94.69	59.83	132.18	23.84	22.65
758.49323	PS 34:2 I PS 16:0_18:2	462.18	852.37	114.92	149.71	59.68	80.02	47.97	42.70
786.49847	PS 35:3;O I PS 18:1_17:2;O	27.28	8.46	40.26	34.61	111.52	93.39	26.02	21.73
788.54254	PS 36:1	65.48	42.82	32.98	24.16	58.51	73.98	28.78	27.57
788.53534	PS 36:1 I PS 18:0_18:1	33972.86	55831.91	4963.17	6613.48	26174.44	70236.02	2747.31	2161.42
818.52655	PS 36:2;2O I PS 18:2_18:0;2O	117.08	62.40	143.95	168.10	73.14	28.01	33.81	7.30
834.51562	PS 36:2;3O I PS 18:0_18:2;3O	1811.59	1354.95	480.34	325.49	1015.59	539.58	537.28	373.21
786.52252	PS 36:2 I PS 18:1_18:1	23371.38	45932.58	1204.97	2360.90	4147.24	11286.49	717.10	873.65
784.50079	PS 36:3 I PS 18:1_18:2	1345.90	2590.37	101.64	194.92	94.69	142.92	38.47	30.82
782.48871	PS 36:4	128.64	54.64	66.51	37.64	231.15	179.64	124.70	53.27

Average *m/z*	Metabolite name	CTR		SCH		NF		PNF	
		mean	*dev*	*mean*	*dev*	*mean*	*dev*	*mean*	*dev*
782.49744	PS 36:4 | PS 16:0_20:4	420.36	627.86	241.77	355.12	68.24	98.25	52.06	21.69
816.56519	PS 38:1 | PS 20:0_18:1	659.00	1005.02	113.54	94.59	1200.62	3336.40	114.27	71.66
814.54565	PS 38:2	16.52	22.38	4.90	5.82	15.44	33.15	3.76	2.91
814.54688	PS 38:2 | PS 18:1_20:1	1680.92	1860.15	843.49	852.42	4955.31	13268.04	521.98	388.05
812.54968	PS 38:3	499.58	203.77	5432.82	11854.71	1087.30	805.38	734.33	507.18
812.57819	PS 38:3 | PS 18:0_20:3	12536.17	8551.81	4611.67	2759.36	7931.18	3914.16	5253.25	2069.28
810.53784	PS 38:4 | PS 16:0_22:4	460.53	616.19	348.95	561.11	111.78	64.34	104.35	44.61
810.52173	PS 38:4 | PS 18:0_20:4	6941.32	9056.02	1633.20	3476.27	4341.59	11057.19	459.89	458.86
810.52594	PS 38:4 | PS 18:1_20:3	987.30	1445.87	129.95	147.63	207.94	271.19	97.85	56.26
808.50568	PS 38:5 | PS 18:0_20:5	344.89	335.88	153.07	155.42	118.01	91.15	58.39	35.75
808.50879	PS 38:5 | PS 18:1_20:4	876.72	1475.55	102.33	146.04	207.95	433.44	55.74	37.86
840.53601	PS 39:4;O | PS 22:3_17:1;O	118.95	108.45	80.04	53.34	218.54	149.14	107.51	79.17
842.57965	PS 40:2 | PS 18:1_22:1	627.94	981.53	223.91	373.62	3433.59	10394.35	74.06	40.87
836.5412	PS 40:5 | PS 18:0_22:5	1876.55	1819.39	729.21	1464.13	640.17	1269.88	112.00	54.64
834.52576	PS 40:6	177.39	179.76	61.01	64.86	78.64	65.29	35.41	19.33
834.52277	PS 40:6 | PS 18:0_22:6	4053.30	4077.80	1317.43	2447.46	1794.97	4266.49	176.44	100.27
832.50366	PS 40:7 | PS 18:1_22:6	597.53	983.66	379.86	690.80	114.44	205.47	61.34	60.07
870.60657	PS 42:2	24.82	43.03	3.92	4.88	10.15	23.88	1.93	1.72
870.60779	PS 42:2 | PS 18:1_24:1	106.29	133.55	32.32	29.69	318.79	920.44	14.81	8.37
868.60162	PS 42:3	1114.61	848.10	1395.30	2564.96	2624.96	3543.73	804.27	597.84
860.5307	PS 42:7 | PS 20:1_22:6	110.29	115.68	59.94	95.44	50.47	98.95	9.68	4.10
938.59052	PS 48:10	39.35	59.91	5.82	6.39	6.91	6.45	2.26	0.82
744.508	PS O-34:2 | PS O-16:1_18:1	2551.37	4769.94	261.13	331.26	1118.61	3163.16	125.38	140.57
770.52448	PS O-36:3 | PS O-18:2_18:1	1079.71	1676.89	230.64	175.57	627.87	1287.91	268.96	248.90
786.56055	PS O-37:2 | PS O-19:1_18:1	3607.67	2744.38	1741.56	1463.91	6034.21	9952.56	2478.63	934.59
750.48242	SHexCer 32:1;2O	114.10	188.60	329.83	553.96	49.89	32.41	77.26	51.10

Average *m/z*	Metabolite name	CTR		SCH		NF		PNF	
		mean	*dev*	*mean*	*dev*	*mean*	*dev*	*mean*	*dev*
778.51416	SHexCer 34:1;2O	2049.45	2688.75	11702.14	19146.34	1343.05	1053.69	1811.46	1176.11
794.50824	SHexCer 34:1;3O	459.26	504.94	555.94	634.44	369.52	185.79	295.07	158.14
776.4978	SHexCer 34:2;2O	379.32	575.60	1897.40	3485.05	284.65	282.85	262.66	175.68
810.54816	SHexCer 35:0;3O	218.73	192.25	1220.42	2191.04	233.57	303.02	186.11	186.92
792.52344	SHexCer 35:1;2O	1140.30	2104.18	5609.23	13765.46	271.06	327.38	891.70	796.82
824.53296	SHexCer 36:0;3O	4780.16	4397.58	3844.65	3106.04	2288.29	1664.28	3431.01	3166.92
806.55243	SHexCer 36:1;2O	361.44	561.47	202.19	326.11	311.41	796.07	166.54	218.46
804.5318	SHexCer 36:2;2O	3387.18	6017.68	11000.93	18160.44	1270.54	1083.01	2777.11	2253.87
838.59424	SHexCer 37:0;3O	13351.69	14059.79	3834.74	2642.62	7250.58	4561.03	4641.13	1944.46
820.55835	SHexCer 37:1;2O	18.26	9.77	27.67	29.29	21.09	16.76	35.76	37.21
834.5448	SHexCer 37:2;3O	1263.61	1237.41	620.81	974.28	300.49	265.59	150.28	31.61
814.50732	SHexCer 37:4;2O	143.12	89.84	118.38	65.93	406.03	1058.53	86.47	41.47
830.53125	SHexCer 37:4;3O	1716.70	3331.97	103.80	95.24	336.58	491.47	225.19	379.58
836.58868	SHexCer 38:0;2O	238.22	245.66	127.42	129.78	255.72	255.96	181.61	105.84
846.54028	SHexCer 38:3;3O	1479.23	893.69	731.75	488.90	1754.23	812.88	822.97	209.79
828.54407	SHexCer 38:4;2O	645.18	509.85	659.61	735.53	1083.75	2287.02	397.13	193.12
842.5246	SHexCer 38:5;3O	589.73	654.90	125.29	75.37	404.16	283.82	117.00	40.31
866.59418	SHexCer 39:0;3O	380.37	326.47	1109.22	2237.19	488.09	478.96	704.24	890.78
858.53534	SHexCer 39:4;3O	126.56	53.54	94.14	65.33	204.78	159.51	103.08	27.81
840.52625	SHexCer 39:5;2O	37.63	26.79	27.03	40.22	39.02	29.16	22.83	9.71
864.61444	SHexCer 40:0;2O	667.04	683.23	314.42	359.77	1491.23	2196.87	1597.29	1770.34
880.60327	SHexCer 40:0;3O	6337.04	5143.10	2501.68	1804.47	3837.49	1274.56	2367.01	1197.12
878.58984	SHexCer 40:1;3O	46397.49	50619.21	36701.73	75500.97	10175.35	18553.51	15770.93	12272.38
874.56366	SHexCer 40:3;3O	184.49	182.02	131.70	76.37	224.05	106.83	88.67	54.35
852.55322	SHexCer 40:6;2O	241.78	254.97	110.98	90.21	59.12	64.02	107.70	85.52
850.52319	SHexCer 40:7;2O	49.11	54.64	19.85	12.87	39.06	34.17	24.74	7.68
894.62292	SHexCer 41:0;3O	1467.17	1175.42	1455.18	2071.23	1727.05	1908.93	1037.43	1034.02

Average m/z	Metabolite name	CTR		SCH		NF		PNF	
		mean	*dev*	*mean*	*dev*	*mean*	*dev*	*mean*	*dev*
886.58148	SHexCer 41:4;3O	77.05	42.29	133.60	234.22	91.67	56.29	49.84	8.06
862.50891	SHexCer 41:8;2O	219.86	147.71	87.03	65.01	166.95	95.46	99.57	26.69
878.50726	SHexCer 41:8;3O	36.99	23.54	22.61	31.38	65.39	70.87	34.94	53.31
908.63239	SHexCer 42:0;3O	169.87	59.04	164.98	89.79	292.75	199.94	214.17	154.38
888.62335	SHexCer 42:2;2O	79020.20	124460.89	100670.50	170923.46	52057.53	49039.94	137522.10	125543.53
886.60669	SHexCer 42:3;2O	11121.68	20452.75	23081.49	52548.51	4418.17	3476.05	5533.79	5133.23
884.59918	SHexCer 42:4;2O	898.00	1050.46	437.61	685.05	1355.73	1953.45	416.26	366.99
878.53094	SHexCer 42:7;2O	226.92	248.65	104.82	93.98	213.52	139.94	102.47	52.93
892.51617	SHexCer 42:8;3O	268.03	170.64	87.26	55.04	115.05	44.90	97.82	78.04
874.53125	SHexCer 42:9;2O	145.34	73.43	180.61	227.17	134.67	149.65	47.47	5.85
922.65369	SHexCer 43:0;3O	26215.84	20214.71	21809.23	23342.58	22691.07	19741.28	18177.87	16608.62
920.64142	SHexCer 43:1;3O	4483.24	2354.07	5644.46	8460.65	3850.50	4006.98	2764.47	2539.31
900.61847	SHexCer 43:3;2O	1792.89	3164.77	1820.95	3756.83	280.97	366.59	556.89	349.40
912.63434	SHexCer 44:4;2O	10558.32	12017.76	3257.78	3182.03	14711.23	21022.63	6387.96	6206.13
936.60498	SHexCer 45:7;3O	48.26	58.36	76.44	163.54	10.95	6.93	74.54	64.54
934.59589	SHexCer 46:7;2O	46.16	74.20	73.35	160.69	16.37	14.23	89.14	89.77
972.73004	SHexCer 48:2;2O	590.55	645.47	371.49	293.97	278.83	362.55	118.65	96.92
966.6886	SHexCer 48:5;2O	440.26	631.56	250.59	328.92	195.23	239.32	255.36	247.28
526.3222	SL 28:4;2O	30.06	13.77	336.61	952.05	12.13	19.32	18.69	13.87
784.66229	SL 46:1;2O|SL 16:0;O/30:1;O	33.96	28.21	47.62	78.68	236.33	277.60	174.51	208.79
856.68323	SL 53:6;O	2051.06	2928.43	3221.13	5665.91	6810.80	8558.26	11451.92	16626.85
391.28131	ST 24:1;O4	29.54	22.69	71.44	124.54	74.66	51.81	64.15	40.60
389.27322	ST 24:2;O4	160.98	210.80	137.14	159.54	234.30	158.36	136.51	123.61
467.32681	ST 27:0;O;S	65.63	72.53	487.37	1413.66	139.60	404.00	17.63	4.88
465.29974	ST 27:1;O;S	2842.35	3329.33	2670.15	4385.75	31376.89	94321.26	448.61	219.91
479.31601	ST 28:1;O;S	1396.57	2105.77	2710.79	5006.62	360.36	320.33	135.89	68.72

**Average** *m/z*	**Metabolite name**	**CTR**		**SCH**		**NF**		**PNF**	
		mean	***dev***	***mean***	***dev***	***mean***	***dev***	***mean***	***dev***
493.3354 5	ST 29:1;O;S	1104.29	1533.48	1126.82	2036.93	419.52	208.73	199.98	91.28
491.3139 6	ST 29:2;O;S	144.40	207.72	180.70	303.87	50.21	37.33	24.32	10.87

Table S3. List of all the sphingolipids species identified using a targeted approach across each histotype. Each sphingolipid was identified with multiple reaction monitoring mass transition (MRM) in positive electrospray ionization, name, and concentration (pmol/mg protein) in each group. The MRM transition were recorded using an optimized declutering potential (DP) and collision energy (CE) for each sphingolipids sublasses: Cer (DP 40 eV, CE 30-40 eV); DHCer (DP 40 eV, CE 35-40 eV); SM (DP 60 eV, CE 40 eV); HexCer (DP 40 eV, CE 50 eV); LacCer (DP 60 eV, CE 60 eV) and GM3 (DP 70 eV, CE 60 eV).

**MRM** *m/z*	**Metabolite name**	**CTR**		**SCH**		**NF**		**PNF**	
		mean	*dev*	*mean*	*dev*	*mean*	*dev*	*mean*	*dev*
510.7 > 264.4	Cer 14	14.85	28.51	12.99	10.30	68.59	85.90	23.69	14.65
538.8 > 264.4	Cer 16	1159.30	1356.06	821.18	525.67	3335.88	3089.40	1110.10	627.66
564.8 > 264.4	Cer 18	0.78	1.46	2.73	5.01	3.00	3.67	1.93	2.35
566.8 > 264.4	Cer 18:1	424.69	507.21	472.74	364.87	702.88	611.04	348.80	245.85
594.8 > 264.4	Cer 20	84.68	106.06	73.75	64.13	170.82	187.02	77.70	51.78
622.9 > 264.4	Cer 22	127.15	174.37	108.02	89.72	330.01	337.85	137.03	80.31
648.9 > 264.4	Cer 24	738.81	871.42	527.62	399.37	1548.31	1350.01	609.17	378.02
650.9 > 264.4	Cer 24:1	790.48	906.77	606.92	505.58	1548.24	2010.38	576.26	287.85
540.4 > 266.4	DHCer 16	45.31	68.24	16.42	22.31	221.63	246.67	83.73	87.06
566.5 > 266.4	DHCer 18	21.51	23.51	7.63	7.24	24.07	25.63	8.93	12.75
568.5 > 266.4	DHCer 18:1	11.56	25.49	6.49	13.35	35.80	61.60	9.33	7.58
650.5 > 266.4	DHCer 24	41.41	70.51	31.03	34.51	112.46	127.36	37.69	31.07
652.5 > 266.4	DHCer 24:1	73.00	187.49	12.23	25.91	28.87	43.25	12.50	11.66
703.5 > 184.1	SM 16	26928.39	30334.78	9937.04	8115.30	39566.16	51380.63	11736.11	8280.05
731.6 > 184.1	SM 18	16216.76	16587.74	7631.38	7561.23	16541.48	16789.10	7330.94	6442.53
729.6 > 184.1	SM 18:1	2796.15	2762.65	2313.87	2237.97	3001.76	2303.99	1294.79	771.89
815.7 > 184.1	SM 24	14091.36	14349.18	5901.97	7122.16	16578.41	22613.53	6037.50	5626.91
813.7 > 184.1	SM 24:1	24283.34	27408.60	8311.89	8704.72	30529.50	39376.65	11044.21	11541.02
700.6 > 264.3	HexCer 16	439.75	586.38	311.09	370.70	404.63	738.48	160.39	103.17
728.6 > 264.3	HexCer 18	1533.50	1563.84	916.71	1301.55	493.68	513.05	1196.79	1443.86
726.6 > 264.3	HexCer 18:1	8.40	12.72	12.43	16.34	0.02	0.06	21.21	17.57

MRM m/z	Metabolite name	CTR		SCH		NF		PNF	
		mean	dev	mean	dev	mean	dev	mean	dev
756.6 > 264.3	HexCer 20	411.65	399.88	229.61	508.61	323.18	465.55	323.39	398.67
784.7 > 264.3	HexCer 22	2669.25	3032.77	1288.63	2899.31	1224.13	1277.87	1710.23	2360.91
812.7 > 264.3	HexCer 24	9667.29	11957.61	4400.21	9674.15	3783.70	3704.41	5361.49	7051.04
810.7 > 264.3	HexCer 24:1	8613.90	9619.57	4472.07	8901.46	4062.86	3453.80	5441.51	6429.39
862.6 > 264.3	LacCer 16	772.25	1187.62	175.08	89.63	655.47	948.18	213.14	114.43
890.7 > 264.3	LacCer 18	48.48	79.25	50.56	51.48	35.43	47.55	17.11	9.38
888.7 > 264.3	LacCer 18:1	0.00	0.00	0.03	0.14	0.00	0.00	0.00	0.00
918.7 > 264.3	LacCer 20	24.09	49.25	12.49	23.62	14.23	24.66	5.67	6.43
946.7 > 264.3	LacCer 22	186.06	308.85	85.65	75.72	181.48	287.62	32.61	22.99
974.8 > 264.3	LacCer 24	309.16	384.12	269.82	293.26	511.69	929.61	62.03	27.17
972.7 > 264.3	LacCer 24:1	984.55	1309.74	395.95	245.79	1005.67	1565.99	203.74	93.50
1153.7 > 264.3	GM3 16	564.44	898.74	221.45	115.71	518.41	561.00	286.29	150.00
1181.7 > 264.3	GM3 18	177.70	370.99	93.41	66.06	132.62	145.67	70.93	54.89
1179.8 > 264.3	GM3 18:1	0.00	0.00	0.53	1.43	0.00	0.00	0.00	0.00
1209.8 > 264.3	GM3 20	80.19	175.44	30.43	27.98	55.43	66.78	34.49	29.45
1237.8 > 264.3	GM3 22	435.71	650.27	227.59	161.14	322.10	265.08	195.76	146.05
1265.8 > 264.3	GM3 24	786.41	1017.83	572.17	518.81	581.60	593.26	306.10	204.12
1263.8 > 264.3	GM 24:1	991.20	1402.63	608.68	371.69	982.32	763.39	562.40	380.55
